# The Slothful Claw: Osteology and Taphonomy of *Nothronychus mckinleyi* and *N*. *graffami* (Dinosauria: Theropoda) and Anatomical Considerations for Derived Therizinosaurids

**DOI:** 10.1371/journal.pone.0129449

**Published:** 2015-06-10

**Authors:** Brandon P. Hedrick, Lindsay E. Zanno, Douglas G. Wolfe, Peter Dodson

**Affiliations:** 1 Department of Earth and Environmental Science, University of Pennsylvania, Philadelphia, PA, United States of America; 2 North Carolina Museum of Natural Sciences, Raleigh, NC, United States of America; 3 Department of Biological Sciences, North Carolina State University, Raleigh, NC, United States of America; 4 White Mountain Dinosaur Exploration Center, Springerville, AZ, United States of America; 5 Department of Animal Biology, University of Pennsylvania, Philadelphia, PA, United States of America; NYIT College of Osteopathic Medicine, UNITED STATES

## Abstract

*Nothronychus* was the first definitive therizinosaurian discovered in North America and currently represents the most specialized North American therizinosaurian genus. It is known from two species, *No*. *mckinleyi* from the Moreno Hill Formation (middle Turonian) in west-central New Mexico, and *No*. *graffami* from the Tropic Shale (early Turonian) in south-central Utah. Both species are represented by partial to nearly complete skeletons that have helped elucidate evolutionary trends in Therizinosauria. In spite of the biogeographical and evolutionary importance of these two taxa, neither has received a detailed description. Here, we present comprehensive descriptions of *No*. *mckinleyi* and *No*. *graffami*, the latter of which represents the most complete therizinosaurid skeleton known to date. We amend previous preliminary descriptions of *No*. *mckinleyi* and *No*. *graffami* based on these new data and modify previous character states based on an in-depth morphological analysis. Additionally, we review the depositional history of both specimens of *Nothronychus* and compare their taphonomic modes. We demonstrate that the species were not only separated geographically, but also temporally. Based on ammonoid biozones, the species appear to have been separated by at least 1.5 million years and up to 3 million years. We then discuss the impacts of diagenetic deformation on morphology and reevaluate potentially diagnostic characters in light of these new data. For example, the ulna of *No*. *mckinleyi* is curved whereas the ulna of *No*. *graffami* was considered straight, a character originally separating the two species. However, here we present the difference as much more likely related to diagenetic compression in *No*. *graffami* rather than as a true biologic difference. Finally, we include copies of three-dimensional surface scans of all major bones for both taxa for reference.

## Introduction

Therizinosaurians are enigmatic theropods known from a substantial number of poorly preserved or only preliminarily described taxa. Half of the currently known species have been described since 2000 and yet few detailed analyses of individual therizinosaurian taxa have been performed. Prior to 2005, there was little consensus regarding the evolutionary relationships of Therizinosauria, and although the increasing number of new taxa discovered in Asia and North America in the past decade permitted the first data-rich phylogenetic analyses of the clade [1, 2], monographic descriptions are still lacking. Such descriptions of therizinosaurian taxa are necessary in order to help resolve some of the remaining ambiguities within the clade.

The early discoveries and analyses of therizinosaurians were hampered by a lack of comparative material and then a lack of well-described material. The first therizinosaurian discovered, *Therizinosaurus*, was originally considered a giant sea turtle because of its gigantic and unique manual unguals [3]. Barsbold [4] and Perle [5] corrected this misidentification on the basis of more complete materials including a referred hindlimb and forelimb. They identified *Therizinosaurus* as a member of Theropoda, linking it with *Segnosaurus*, which was described in 1979 [6]. The same year, another therizinosaurid, *Nanshiungosaurus brevispinus*, known from a series of vertebrae and an incomplete pelvic girdle, was described, yet originally considered a sauropod [7]. However, the analysis of a second species referred to this genus, *Na*. *bohlini* [8], demonstrated that *Nanshiungosaurus* was most closely related to therizinosaurians. *Erlikosaurus* is the only derived therizinosaurian with a well-preserved, three-dimensional skull and was originally named by Perle [9] and later re-described by Clark et al. [10] and Lautenschlager et al. [11]. These early discoveries were restricted to derived taxa with highly modified bauplans and there was a lack of overlapping skeletal material between taxa. Thus the relationships within the clade and with respect to other theropod clades remained contentious. The analysis of the more complete taxon *Alxasaurus* [12] firmly established the clade as members of Theropoda, and suggested a close relationship with Oviraptorosauria. Although early descriptions and phylogenetic analyses of these taxa and others from Asia improved our knowledge of Therizinosauria [13–15], they did not include robust phylogenetic analyses, largely because most species were still known from fragmentary remains.

The subsequent discovery and description of several North American taxa, including *Nothronychus mckinleyi* [16], *Falcarius utahensis* [1], and *Nothronychus graffami* [2], provided the most complete skeletal material of both unspecialized and specialized therizinosaurians to date. Kirkland et al. [1] and Zanno et al. [2] performed the first robust phylogenetic analyses using this new material, which placed Therizinosauria at the base of Maniraptora. By 2010, twelve therizinosaurian taxa had been named. However, the vast majority of descriptions were brief and no species other than *Falcarius* [17, 18] had been reanalyzed in light of new specimens, making the descriptions, autapomorphies, and synapomorphies of most taxa out of date. Zanno [19] reevaluated the taxonomy and phylogeny of all therizinosaurian taxa, updating diagnoses. However, the necessary breadth of the review format in Zanno’s study [19] precluded a detailed morphological description of any specific taxon.

**Fig 1:**
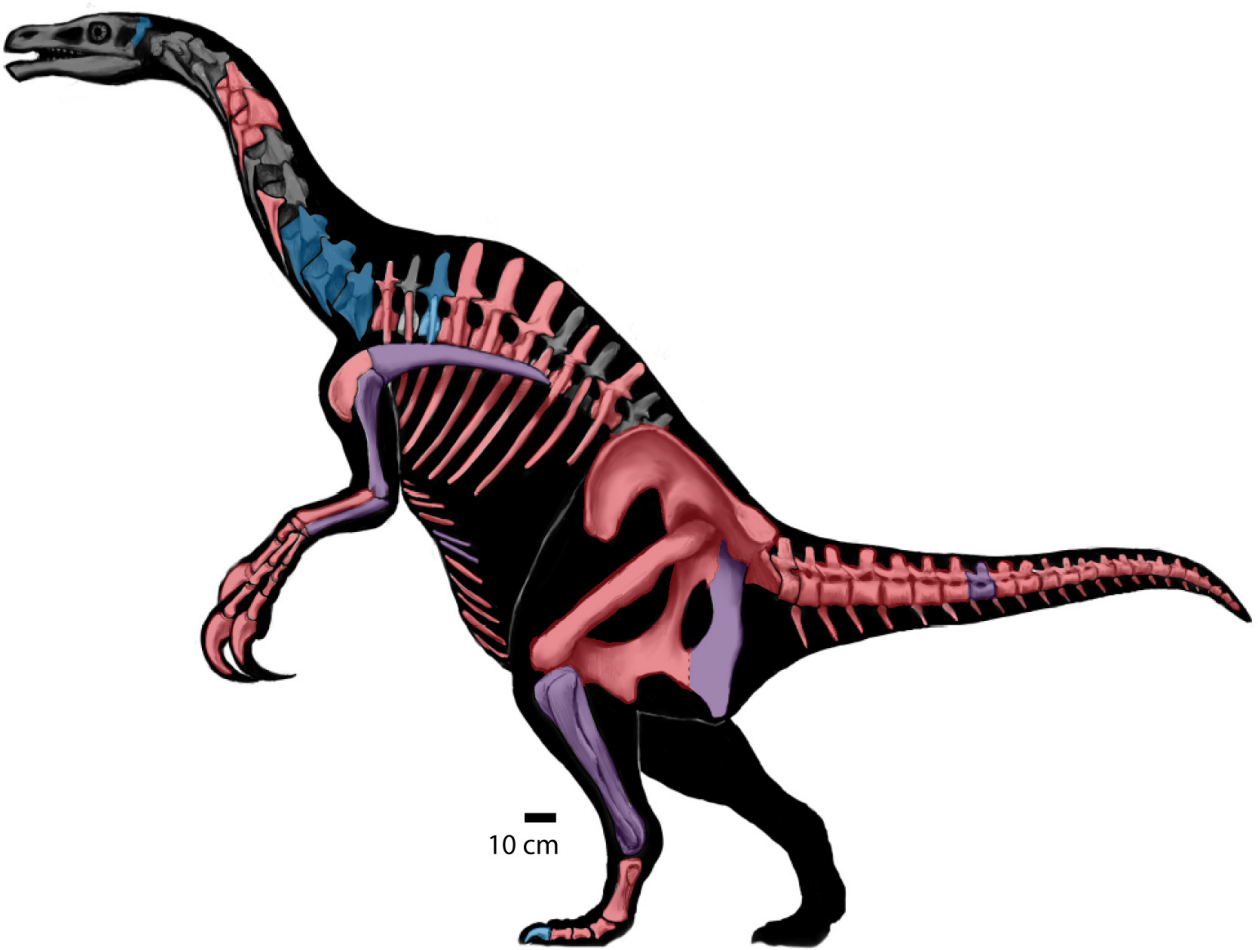
*Nothronychus*. Skeletal reconstruction of *Nothronychus* sp. (based on Zanno et al. [2]). Blue elements indicate presence in MSM P2117 (*No*. *mckinleyi*). Red elements indicate presence in UMNH V16420 (*No*. *graffami*). Purple elements are present in both specimens of *Nothronychus*.

*Nothronychus mckinleyi* was the first definitive therizinosaurian discovered in North America. It was excavated during the early to mid-1990s, was first described in 2001, and has important implications for the biogeography and diversity of therizinosaurians [16]. Since *No*. *mckinleyi* was described, three new therizinosaurians have been named from North America: *Falcarius utahensis* [1] the most basal therizinosaurian known to date, *Nothronychus graffami* [2] the most complete therizinosaurid known to date, and *Martharaptor greenriverensis*, tentatively referred to Therizinosauria based on highly fragmentary materials [20]. Although incredibly important from a phylogenetic and biogeographical perspective, both species of *Nothronychus* have been only preliminarily described [2, 16] with the exception of the braincase of *No*. *mckinleyi* [11, 21, 22]. Considering the importance of these specimens, we present a detailed description and reevaluation of the two species of the North American therizinosaurid, *Nothronychus* herein (Fig 1).

## Materials and Methods

The holotypes of *No*. *mckinleyi* (MSM P2117 [Mesa Southwest Museum, Mesa, AZ, USA]) and *No*. *graffami* (UMNH VP16420 [Natural History Museum of Utah, Salt Lake City, Utah, USA]) were examined, measured, photographed, and surface scanned at the Arizona Museum of Natural History in Mesa, Arizona and the Utah Museum of Natural History in Salt Lake City, Utah, respectively. Both *No*. *mckinleyi* and *No*. *graffami* are known exclusively from holotype materials comprising large percentages of the skeletons. Elements smaller than 300 mm were measured using 300 mm Mitutoyo 500–173 digital calipers. Elements larger than 300 mm and elements with sharp angles were measured using a soft measuring tape. All measurements are listed in the supplemental information (S1 Table).

In addition to photographs and measurements, the majority of elements of both specimens were surface scanned. The scans allow a three-dimensional representation of elements so that it is possible to examine purported characters digitally. Scans were taken using the Polhemus FastSCAN system, which allows for full rotation of each specimen during scanning. The scans were then post-processed using Geomagic and were reduced to 60,000 triangles and smoothed to reduce noise generated during the scanning process. This number was chosen as it produced a relatively small file without sacrificing noticeable 3D information. For MSM P2117, we scanned the antebrachium; caudal vertebra; two gastralia; left scapula; right and left humerus; right ulna; left ischium; right and left tibiae; right fibula; a partial metatarsal element; a manual phalanx; and a pedal claw. For UMNH VP16420, we scanned three caudal vertebrae; left scapula; right humerus; right ulna and radius; left manual ungual II; left ischium and pubis; right femur; and left tibia and fibula. We considered relative completion and availability of comparative materials across skeletons when choosing elements to scan. We avoided scanning presacral vertebrae because of limitations reproducing highly pneumatic bones with accuracy using laser surface scanners. All scans are included in the supplemental information and can easily be visualized using the GNU software, MeshLab (MeshLab, Visual Computing Lab—ISTI—CNR http://meshlab.sourceforge.net/) (S1 Multimedia).

In order to fully evaluate the taphonomic setting of the MSM P2117 bonebed, we compiled angle measurements of all elements taken while *in situ* and broke the data up into groups based on length: width aspect ratios. Low aspect ratios include somewhat rounded bones such as vertebrae and high aspect ratios include elongate bones such as appendicular bones. These data were then plotted in MATLAB using the rose function in order to evaluate angle trends (S1 Table).

## Results

### Systematic Paleontology

Dinosauria [23]

Theropoda [24]

Maniraptora [25]

Therizinosauria [26] sensu [19]

Therizinosauridae [3] sensu [2]

*Nothronychus* [16]

Etymology: *Nothros* (Greek): slothful; *onyx* (Greek): claw (after [16])

Diagnosis: *Nothronychus* can be differentiated from all other therizinosaurians by the following autapomorphies: 1) distinctly subcircular obturator process [2]; 2) ovoid, dorsoventrally elongate obturator foramen (maximum length greater than 200% of the maximum width) [2]; 3) contact between pubis and ischium restricted to proximal half of the obturator process [2]; and 4) presence of a deep notch between the ventral aspect of the obturator process and cranial ischial shaft [2].

*Nothronychus mckinleyi* [16]

Occurrence: Moreno Hill Formation, southern Zuni Basin, Catron County, New Mexico, Late Cretaceous, middle Turonian [27].

Holotype (after [2]):

MSM P2117, partially disarticulated skeleton including isolated teeth, partial braincase, two potential cranial fragments, fragments of seven cervical vertebrae, one cranial dorsal vertebra, one dorsal centrum, one mid-caudal vertebra, cervical and dorsal ribs, fused gastralia, chevron fragment, left scapula, right humerus, complete right ulna, unidentified antebrachial shaft, distal metacarpal II, manual PI-1 and PII-1, both ischia, both tibiae, right fibula, partial MTII, pedal PI-1, PIV-4, PIV-2, and two pedal unguals.

Diagnosis:

*Nothronychus mckinleyi* can be differentiated from all other therizinosaurians by the following autapomorphies: 1) ventral notch between obturator process and iliac shaft craniocaudally narrow [2]; and from *N*. *graffami* by the following combination of features: 1) platycoelous cranial caudal centra [2]; 2) heel on caudoventral surface of caudal centra hypertrophied (approximately 1/3 the dorsoventral length of the caudal centrum) [2]; 3) glenoid lacking caudal buttress [2]; 4) caudal process of ischium poorly defined and elongate, extending to shaft opposite obturator process [2].

*Nothronychus graffami* [2]

Occurrence: Tropic Shale, 65 m above the local top of the Dakota Formation; 5 meters below the lowest occurrence of the *Mammites nodosoides* Ammonoid Biozone [28] (early Turonian), Kaiparowits Basin, Kane County, UT, USA.

Holotype (after [2]):

UMNH VP16420, nearly complete postcranial skeleton consisting of two cervical vertebrae, nine dorsal vertebrae, a complete sacrum, and 20 caudal vertebrae, both scapulae, both partial coracoids, both humeri, ulnae, radii, much of the manus including three metacarpals and numerous phalanges and manual claws, both ilia, both ischia though badly damaged, both pubes, femora, tibiae, and fibulae, as well as a nearly complete pes with all tarsal bones.

Diagnosis:

A therizinosaurid with the following autapomorphies: 1) pubic boot with diminutive caudal process [2]; 2) ventral margin of pubic boot dorsally convex [2]; 3) subtriangular caudal process of ischium diminutive (less than 5% of the total length of ischium) [2]; and 4) caudal process of ischium located proximal to obturator foramen [2]. *Nothronychus graffami* can be differentiated from *Nothronychus mckinleyi* by the following four features: 1) strongly amphicoelus cranial caudal centra [2]; 2) caudoventral aspect of caudal centra poorly developed [2]; 3) glenoid with pronounced caudal buttress [2]; and 4) ventral notch between obturator process and iliac shaft craniocaudally wide [2]. *(Zanno et al. [2] included “ulna straight”, which was shown here to be caused by diagenetic distortion).

### Osteological Description

#### Braincase

A partial braincase is known for MSM P2117 (*No*. *mckinleyi*); it has been intensively studied, yet never photo documented in color. The braincase was first described by Kirkland et al. [21] and was recently redescribed in two separate publications [11, 22]. Lautenschlager et al. [11] used commuted tomography data to correct many previous ambiguities resulting from the incompleteness of the braincase, complete or near-complete obliteration of the sutures, and difficulties distinguishing between pneumatic cavities and canals invading the endocranium.

**Fig 2:**
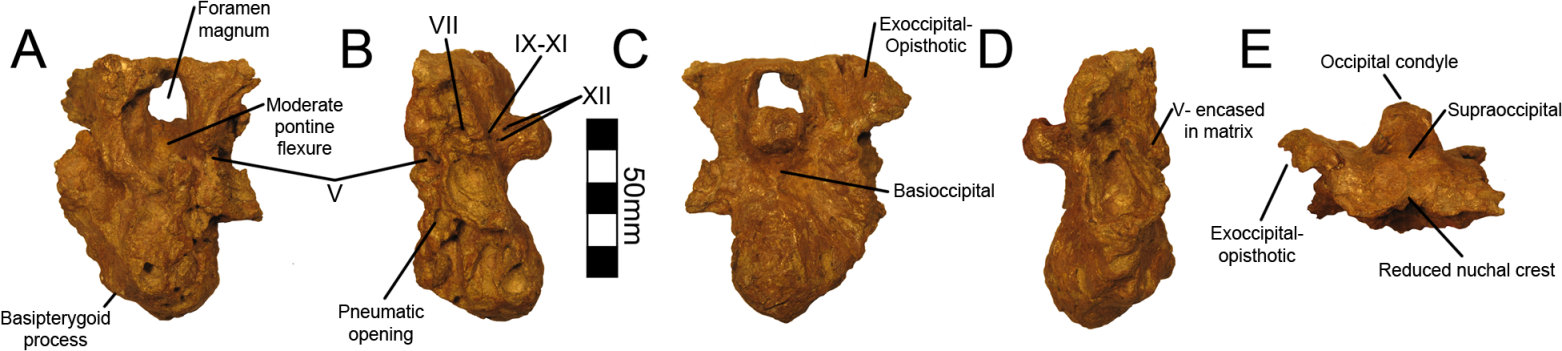
*Nothronychus* (MSM P2117) braincase. The braincase preserved with MSM P2117 in (A) cranial, (B) left lateral, (C) caudal, (D) right lateral, and (E) dorsal views. Scale = 50 mm.

Four therizinosaurian taxa preserve three-dimensional braincases: *No*, *mckinleyi*, *Erlikosaurus andrewsi* [10], *Neimongosaurus yangi* [14], and *Falcarius utahensis* [1, 29]. The braincase of *Neimongosaurus* was not figured or described by Zhang et al. [14] and has not been analyzed as part of this study. Two additional taxa preserve highly compressed braincases—*Beipiaosaurus inexpectus* [13] and *Jianchangosaurus yixianensis* [30]—yet neither has been described. As a result, comparisons to date are restricted to three therizinosaurian taxa. Here we summarize the descriptive work of Lautenschlager et al. [11] and Smith [22], as well as provide figures of the original fossil material based on conclusions that were primarily garnered from CT data [11] (Fig 2).

Lautenschlager et al. [11] provided a complete comparative description of the endocranium of *No*. *mckinleyi* and reconstructed the semicircular canals. As with the other therizinosaurians, MSM P2117 has a highly pneumatic and ventrally expanded braincase [21]. MSM P2117 preserves the supraoccipital, exoccipital, basioccipital, opisthotic, prootic, basisphenoid, and a partial laterosphenoid [22]. Using CT data, Lautenschlager et al. [11] reconstructed cranial nerves V, VI, VII, IX, X, XI, and XII. All three branches of the trigeminal nerve (CN V) were visible [11] and exit through the foramen originally suggested by Kirkland et al. [21]. Smith et al. [29] suggested that the maxillary and mandibular branches of the trigeminal nerve came through the canal originally suggested to house the abducens nerve by Kirkland et al. [21], but CT data revealed that this region is a blind pneumatic space and does not open to the endocranial cavity [11]. Smith [22] further argues that the ophthalmic branch of the trigeminal nerve exits through a foramen separate from the maxillary and mandibular branches; however, this is not supported by CT data [11]. The abducens nerve canal is filled with matrix and cannot be seen on the preserved specimen; it is caudal to the trigeminal canal [11]. The facial nerve also exits caudal to the trigeminal canal [11]. A foramen not originally identified by Kirkland et al. [21] is presented as the exit for the glossopharyngeal, vagus, and accessory nerves based on similarities to *Erlikosaurus* [11]. Finally, there are two small foramina on the caudal aspect of the braincase lateral to the occipital condyle that transmit branches of the hypoglossal nerve [11].

Lautenschlager et al. [11] reconstructed the semicircular canals as rectangular, in comparison with the triangular condition found in other theropods. Absence of a pronounced nuchal crest [21, 22] and a reduced adductor chamber [22] spurred hypotheses of low bite force in *No*. *mckinleyi* [22]. Lautenschlager [31] used three-dimensional lever models to reconstruct low bite forces in *Erlikosaurus andrewsi*, which would support low bite force estimates in MSM P2117 and therizinosaurids generally. However, until more skull material is recovered, it will not be possible to test the reliability of this result specifically for *No*. *mckinleyi*.

#### Teeth

**Fig 3:**
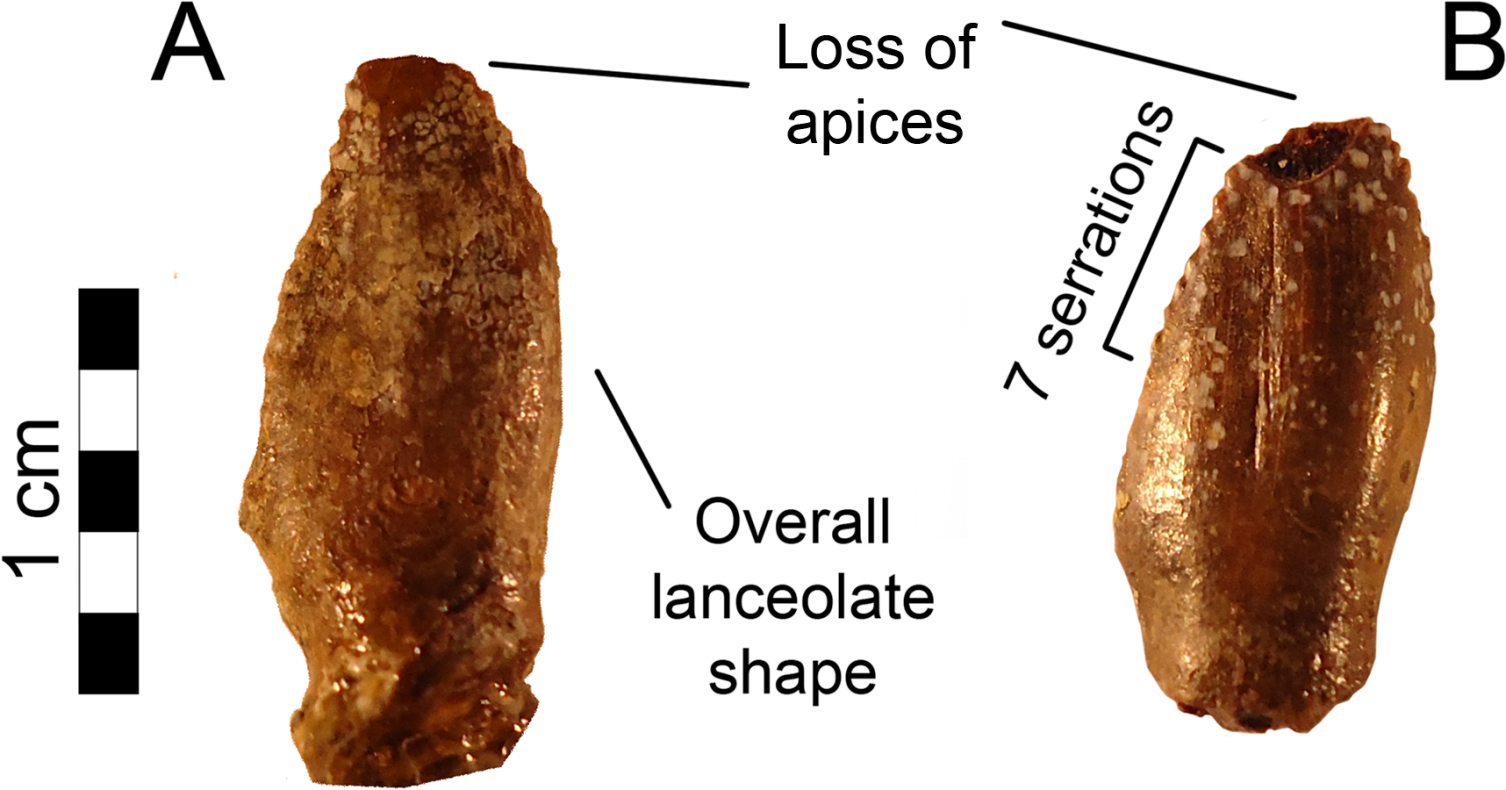
*Nothronychus* (MSM P2117) teeth. (A) Distal tooth preserved with MSM P2117. (B) Distal tooth preserved with MSM P2117. Scale = 5 mm.

Two teeth are preserved in MSM P2117 (*No*. *mckinleyi*). Both are nearly complete bearing a portion of the root, yet missing the apexes (Fig 3). The teeth are lanceolate, serrated, tall, and mesiodistally narrow, as is typical for therizinosaurians such as *Falcarius*, *Eshanosaurus*, and *Erlikosaurus* [1, 10, 32, 33, 34]. The mesial teeth in *Falcarius* and *Erlikosaurus* are conical and the more distal teeth are lanceolate [1, 10]. Although the teeth are not preserved *in situ* within the mandible of *No*. *mckinleyi*, their relative proportions compare most closely to the dentary teeth of *Erlikosaurus*, suggesting that they derive from the dentary.

*Erlikosaurus* has relatively symmetrical teeth, especially in the maxilla, such that the mesial and distal surfaces of the crown are approximately equal in size and angle of deflection (10–15º) [10]. *Beipiaosaurus*, *Jianchangosaurus*, and *Eshanosaurus* preserve teeth in which the mesial surface is slightly convex giving the teeth a slight recurvature. The distal teeth in *Falcarius* have a much stronger recurvature [1]. The convexity of the mesial surface of the crown in MSM P2117 is more pronounced, although less so than in *Falcarius*. The distal surface is slightly concave and the tip of the tooth is slightly recurved. The serrations are larger and coarser than in *Eshanosaurus* and *Falcarius* and most resemble *Erlikosaurus* in their size and number relative to the size of the tooth. The better-preserved tooth preserves eight closely spaced serrations on the mesial surface and seven on the distal surface. Several more serrations were likely present on both aspects along the missing tip. MSM P2117 has teeth that have 2.5 denticles per millimeter mesially and 1.8 denticles per millimeter distally. In contrast, the teeth in *Eshanosaurus* have 3.5 denticles per millimeter mesially and 4.4 denticles per millimeter distally [33] and *Falcarius* has seven denticles per millimeter distally [18]. Each denticle is proportional to those of *Erlikosaurus* and larger than denticles in other maniraptorans [10].

#### Cervical Vertebrae

**Fig 4:**
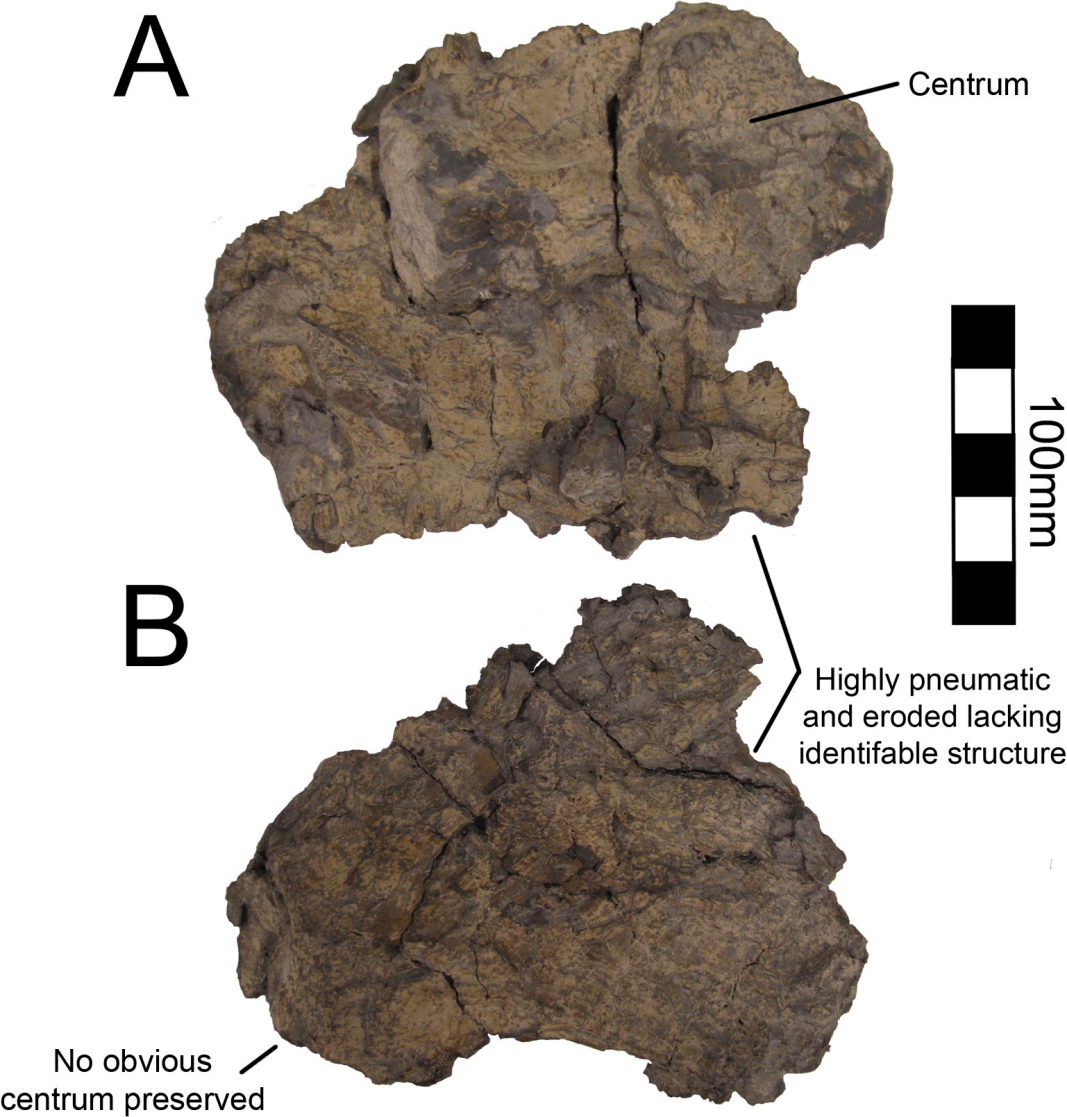
*Nothronychus* (UMNH VP16420) cervical vertebrae. (A) Poorly preserved caudal cervical. (B) Poorly preserved caudal cervical. Scale = 100 mm.

**Fig 5:**
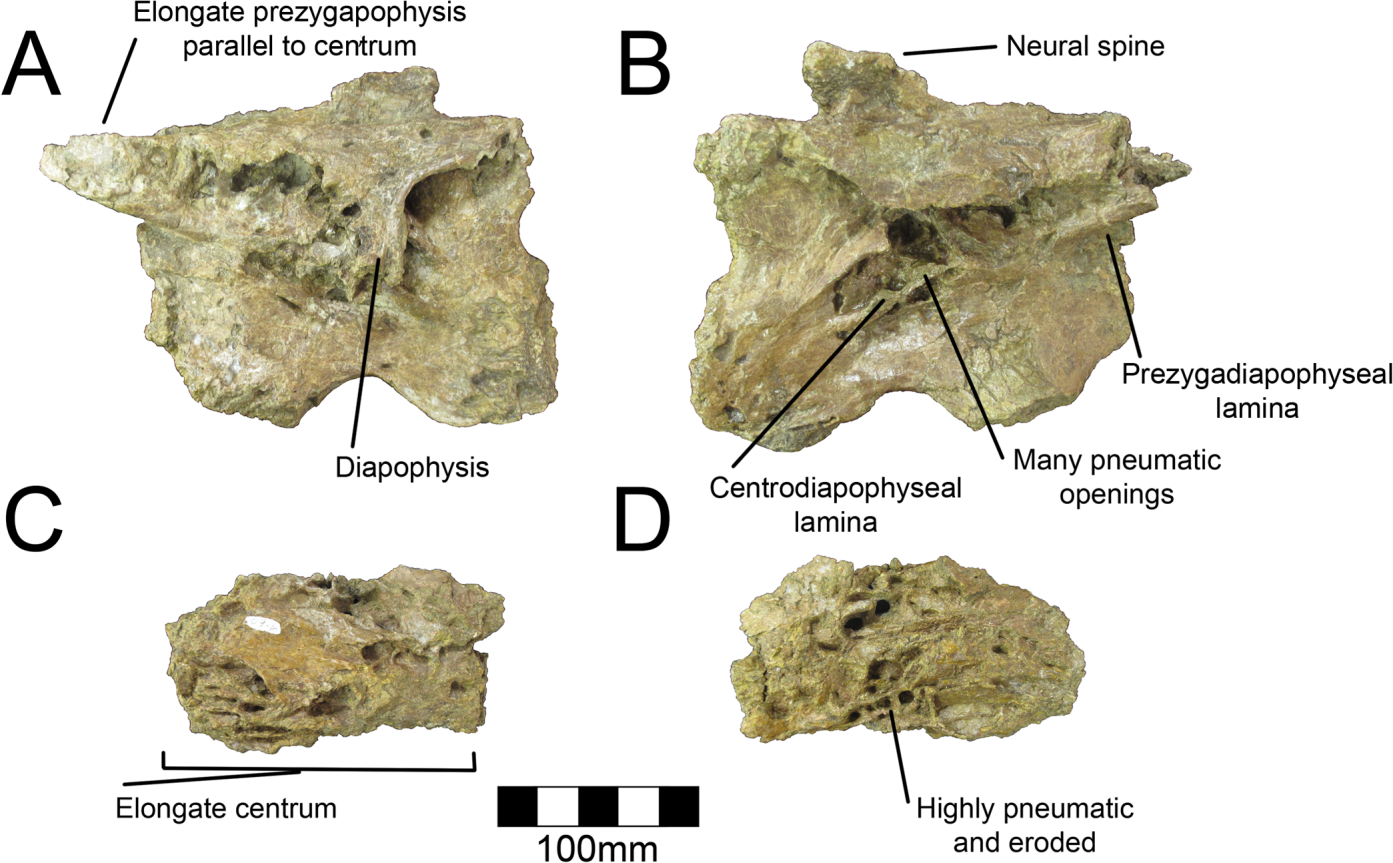
*Nothronychus* (MSM P2117) cervical vertebrae. Large caudal cervical in (A) left lateral view and (B) right lateral view. Small pneumatic cervical vertebra in (C) left and (D) right views. Scale = 100 mm.

There are numerous cervical vertebral elements associated with MSM P2117 (*No*. *mckinleyi*) representing a minimum of seven vertebrae and two partial cervicals preserved with UMNH VP16420 (*No*. *graffami*). However, all of the cervical vertebrae in both taxa are badly diagenetically distorted and fragmentary (Figs 4 and 5). As such, it is not possible to determine the exact position of any vertebra. The length of the cervical column is not well known in therizinosaurians, though many of the known taxa preserve several cervical vertebrae. *Jianchangosaurus* (n = 10), *Beipiaosaurus* (STM31-1 [Shandong Tianyu Museum of Nature, Shandong, China]) (n = 9), *Nanshiungosaurus* (n = 11), and *Neimongosaurus* (n = 13) are the only articulated therizinosauroids known that preserve all of their cervical vertebrae [7, 14, 30, 35]. *Neimongosaurus* and *Nanshiungosaurus* bracket *Nothronychus* phylogenetically [19] providing a possible upper and lower bound for the cervical vertebral count. Numerous cervical vertebrae are known for *Falcarius* [18], but the actual vertebral count is unknown, as all *Falcarius* material is known from a disarticulated bonebed. Five cervicals are known from *Alxasaurus*, but they are not articulated and do not represent a complete column [12].

The cervical vertebrae are too deformed to provide information about their order in either species of *Nothronychus* (as in *Alxasaurus*, [12]). This is exacerbated by the fact that cervical vertebrae in therizinosaurians are generally homogenous in form [8]. The three best-preserved cervicals pertain to MSM P2117 and are here described and labeled 1–3 for descriptive purposes. There are no obvious differences in the morphology of MSM P2117 and UMNH VP16420 cervicals due to distortion. The atlas and axis are not preserved in either MSM P2117 or UMNH VP16420. Based on their large size, the three best-preserved cervicals of MSM P2117 were likely the caudalmost cervicals. Cervical #2 (MSM P2117) is the best-preserved cervical vertebra (Fig 5). Both cervical #1 and #3 (MSM P2117) are comparatively well preserved, but do not preserve the condition of the pleurocoels as well as #2. All three vertebrae are approximately the same size, though cervical #2 is the largest and all are transversely compressed. There are some additional fragments of the ventral neural arch that have been crushed dorsoventrally allowing for a complete picture of a generalized cervical vertebra in MSM P2117. The following description refers to the structures of cervical #2 unless otherwise noted and only applies to *No*. *mckinleyi*. The morphology of the cervicals in UMNH VP16420 is too distorted to allow comment on *No*. *graffami*.

The pre- and postzygapophyses are elongate and robust as in *Na*. *bohlini* [8]. The left prezygapophysis is parallel to the long axis of the vertebra. The postzygapophyses are damaged so their orientation cannot be determined. The left diapophysis is partially preserved; however, the distalmost extent is missing. It is oriented ventrally due to deformation. Both cervical #1 and #2 preserve camellate pneumatization of the centrum. Cervical #3 has relatively less pneumatization. In cervical #2, multiple large pleurocoels pierce the base of the transverse processes; however it is difficult to determine which owe their external expression to cortical bone loss, and which would have originally opened externally (Fig 5A and 5B). Ventral to the largest pleurocoel on the lateral aspect are two smaller pleurocoels separated by the posterior centrodiapophyseal lamina, which extends from the diapophysis to the caudal aspect of the centrum. It is somewhat diagonally directed in MSM P2117, likely due to diagenetic distortion. This feature is preserved on both sides, though it is more evident on the right side. The postzygadiapophyseal and prezygadiapophyseal laminae are present and are especially visible on the right side. Pleurocoels and buttresses are well developed in *Neimongosaurus* [14]. They are incipient in the more basal forms, *Falcarius* and *Beipiaosaurus* [18], but are somewhat developed in *Jianchangosaurus* [30].

The dorsal aspect of the vertebra is flattened and the pre- and postzygapophyses are at the same level in lateral aspect. This appears to be an original feature and not the result of diagenetic deformation. The neural canal is also at the level of the zygapophyses as in *Na*. *bohlini* [8]. The prezygaparapophyseal laminae are thin and this compression continues across the dorsal aspect of the neural arch, roofing the neural canal with only a thin layer of bone. The neural spine is incipient and twice as long axially as it is tall (Fig 5A and 5B). This is also the case in *Alxasaurus*, *Beipiaosaurus*, and *Na*. *bohlini* [8, 12, 13].

The ventral aspect has a deep ventral groove extending from the cranial end to the caudal end. The groove is deepest cranially and lessens in its excavation caudally as in *Na*. *bohlini* and *Falcarius* [8, 18]. The faces of the centra are distorted, but appear to be slightly biconvex and not amphiplatyan (as in [16]). The cervicals are amphiplatyan in *Alxasaurus*, *Na*. *bohlini*, and amphicoelous in *Neimongosaurus* and *Falcarius* [8, 12, 14, 18] documenting variation within Therizinosauria. Furthermore, mid-cervicals of *Falcarius* are amphicoelous, whereas caudal cervicals are platycoelous, demonstrating variation within the cervical series within individual therizinosaurian taxa as well [18].

A single cervical rib is preserved disarticulated from the vertebral body in MSM P2117 (S1 Fig). The caudal ramus is complete, but the cranial end is broken so the cranialmost extent of the rib is not known. Due to the fragmentary nature of the cervical vertebrae, it is not possible to associate the cervical rib with a particular vertebra. The rib has a large poorly preserved articular structure such that the tuberculum and capitulum cannot be distinguished. The caudal ramus as preserved is longer than the cranial ramus and this seems to be a definitive feature as the broken ends are both tapering. The rami form a 90° angle and each have a pronounced buttress extending along their length with the caudal buttress being more pronounced than the cranial buttress. There are three smaller buttresses extending a short distance down the cranial branch of the rib giving the rib a stiffened appearance not likely to have substantial bending capacity.

#### Dorsal Vertebrae

**Fig 6:**
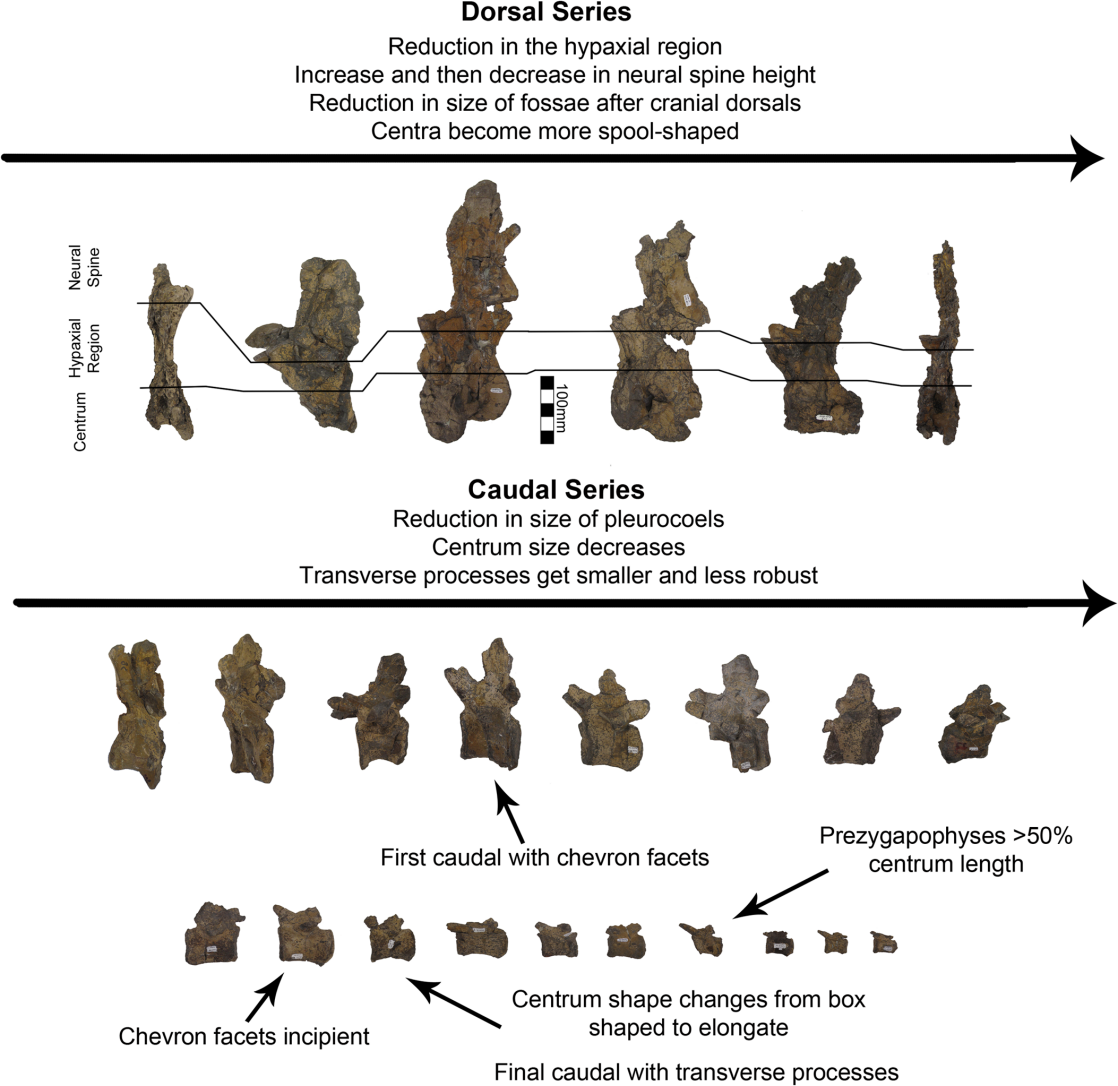
*Nothronychus* (UMNH VP16420) dorsal and caudal series. Six representative dorsal vertebrae and eighteen representative caudal vertebrae demonstrating large scale changes along the vertebral column. Scale = 100 mm.

A single dorsal vertebra with a complete centrum and most of the neural arch is preserved with MSM P2117 (*No*. *mckinleyi*). Nine dorsal vertebrae representing the majority of the dorsal column are preserved with UMNH VP16420 (*No*. *graffami*). However, unlike the dorsal preserved with MSM P2117, all UMNH VP16420 dorsals are diagenetically distorted, primarily along a craniocaudal axis. The best preserved cranial dorsal of UMNH VP16420 is similar to that of MSM P2117 except that it is slightly smaller. It is likely that the MSM P2117 dorsal is a second or third dorsal and the cranial dorsal in UMNH VP16420 is the first dorsal as shown by Zanno et al. [2]. It would appear that UMNH VP16420 does not preserve either dorsal two or three making a direct comparison between the two species on the basis of dorsal morphology difficult. Generally, cranial dorsals are easily distinguishable from both mid-dorsals and caudal dorsals in therizinosaurians. As in *Falcarius*, and presumably most therizinosaurians, the dorsal vertebrae grade between three distinctive morphotypes in UMNH VP16420 [18]. The cranial dorsals possess the deepest infradiapophyseal and infrazygapophyseal fossae, most extreme hypaxial height, and the shortest neural spines. The middle dorsals have more spool-shaped centra, long neural spines, and less heightened hypaxial regions. The caudal dorsals have more incipient neural spines than the middle dorsals and reduced hypaxial regions (Fig 6).

**Fig 7:**
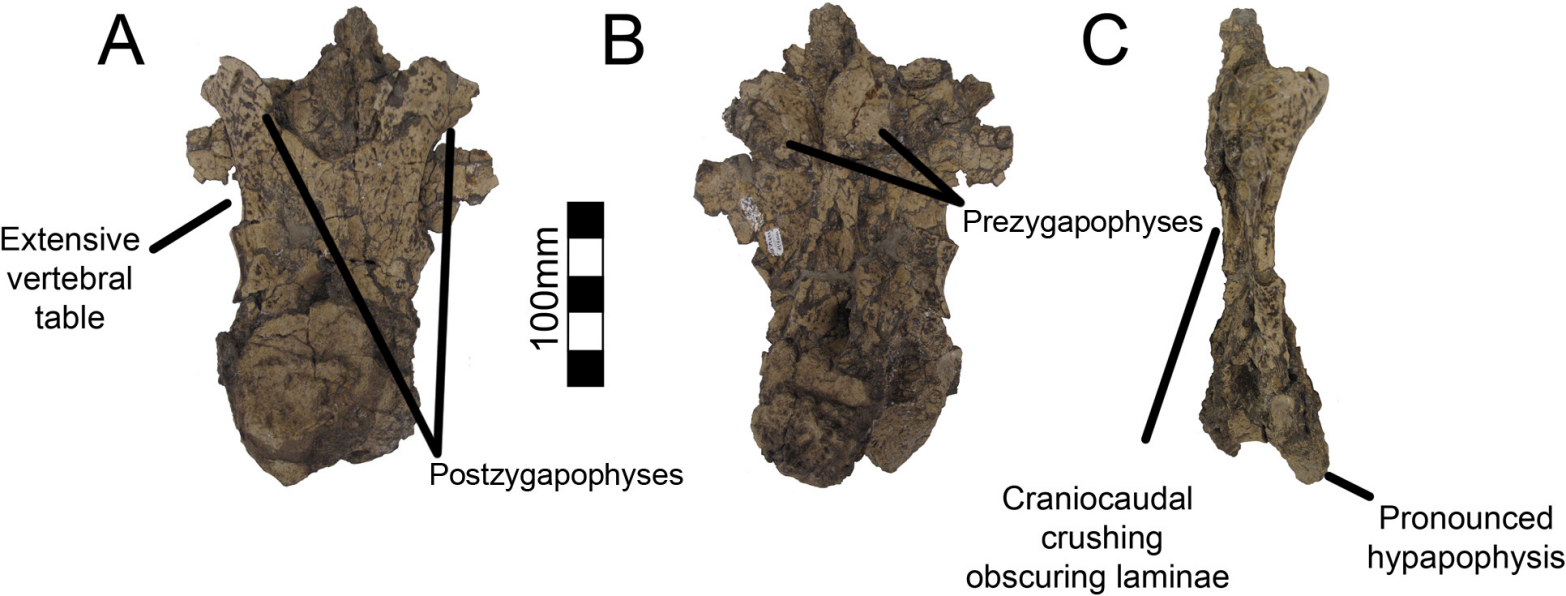
*Nothronychus* (UMNH VP16420) cranial dorsal vertebra. Dorsal vertebra preserved in (A) caudal, (B) cranial, and (C) left lateral view. Figure explanations on figure. Scale = 100 mm.

**Fig 8:**
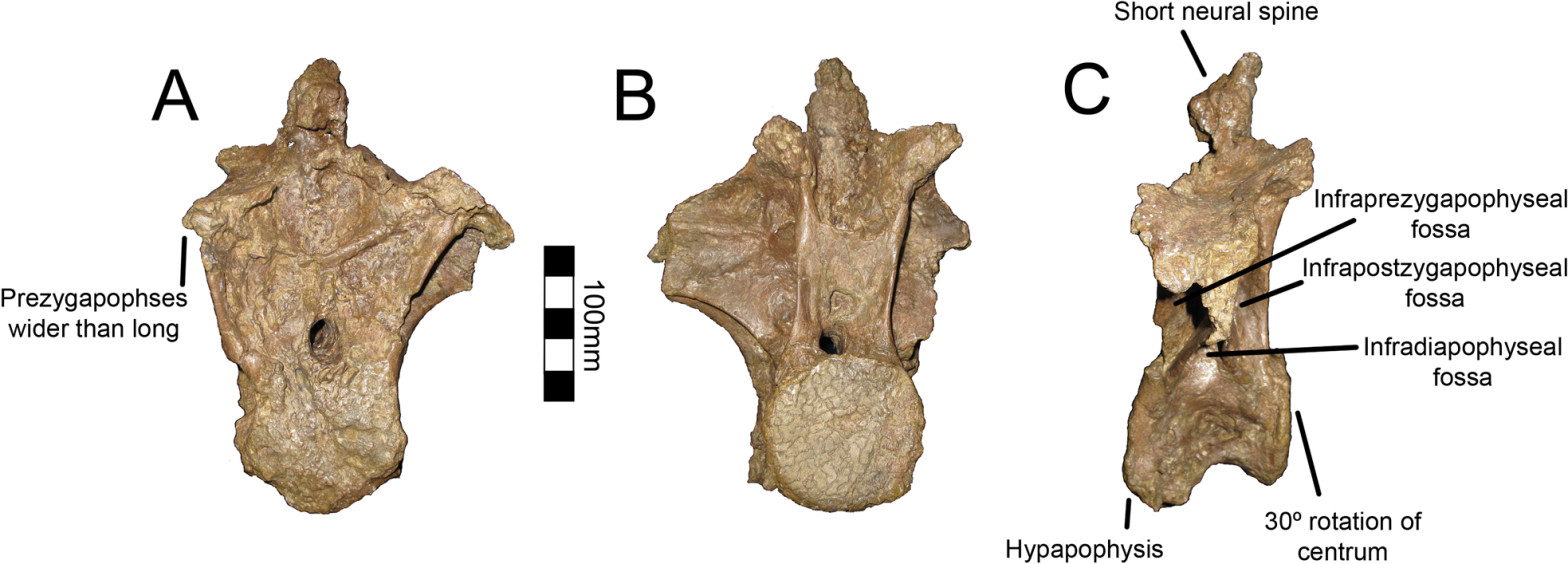
*Nothronychus* (MSM P2117) cranial dorsal vertebra. Single dorsal vertebra preserved in (A) cranial, (B) caudal, and (C) left lateral view. Figure explanations on figure. Scale = 100 mm.

Dorsal one of UMNH VP16420 is axially flattened making it difficult to discern individual laminae, as openings in bone are the most susceptible to deformation [36, 37] (Fig 7). Centrozygapophyseal laminae were likely present in UMNH VP16420 since they are clearly developed in MSM P2117 (Fig 8), *Falcarius*, *Alxasaurus*, *Na*. *brevispinus*, and *Neimongosaurus* [7, 12, 14, 18]; however, the crushing has largely obscured them. It is unknown if pleurocoels were present or absent in these regions in *No*. *graffami*, the latter being the case in *Jianchangosaurus* and *Erliansaurus* [15, 30]. However, it should be noted that *Jianchangosaurus* is preserved as a slab and the lack of pleurocoels may be related to diagenetic deformation. The lateral pneumatopores of MSM P2117 (dorsal two? in the column) are divided into infraprezygapophyseal, infradiapophyseal, and infrapostzygapophyseal fossae as in other therizinosaurians (Fig 6) [7, 12, 18] with well-formed centrozygapophyseal and centrodiapophyseal laminae. The cranial aspect of the vertebral table is very flat, as in *Alxasaurus* [12], with extensive prezygapophyses that are wider transversely than axially long, though this feature could be diagenetic (Fig 8). The postzygapophyses are flattened ventrally. As in other therizinosaurians, they are located above the centrum and had robust centrozygapophyseal laminae. The dorsal centrum itself is extremely shortened in comparison with cervical vertebrae as in other therizinosaurians [18]. As in *Erliansaurus* and *Neimongosaurus*, MSM P2117 and UMNH VP16420 both preserve a 30° angle between the long axis of the centrum and the neural arch and a robust hypapophysis (Figs 7 and 8) [14, 15].

**Fig 9:**
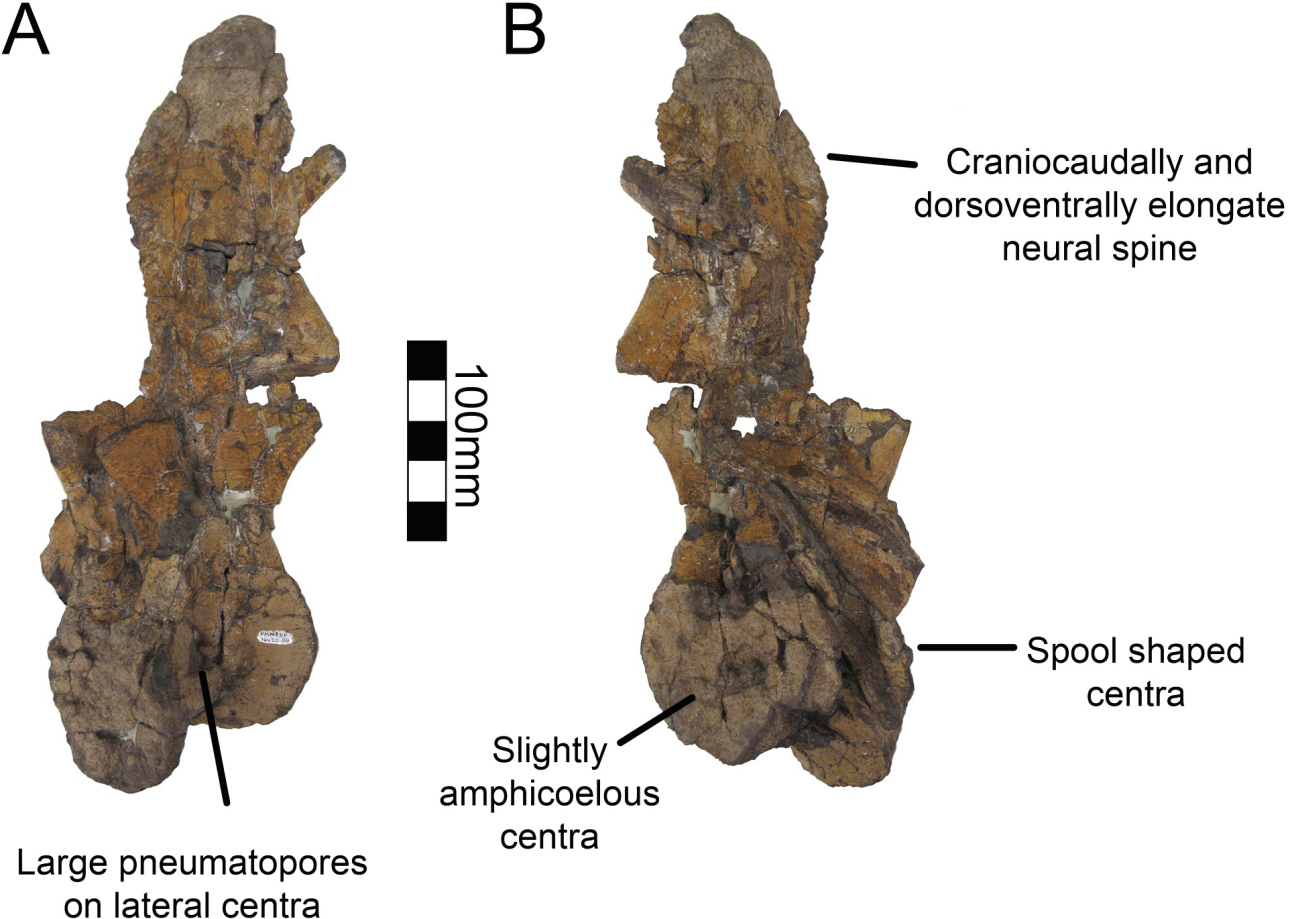
*Nothronychus* (UMNH VP16420) middle dorsal vertebra. Mid-dorsal vertebra preserved in (A) left lateral and (B) right lateral views. The vertebra has been plastically crushed squashing many of the cranial and caudal features to the sides of the vertebra. Figure explanations on figure. Scale = 100 mm.

**Fig 10:**
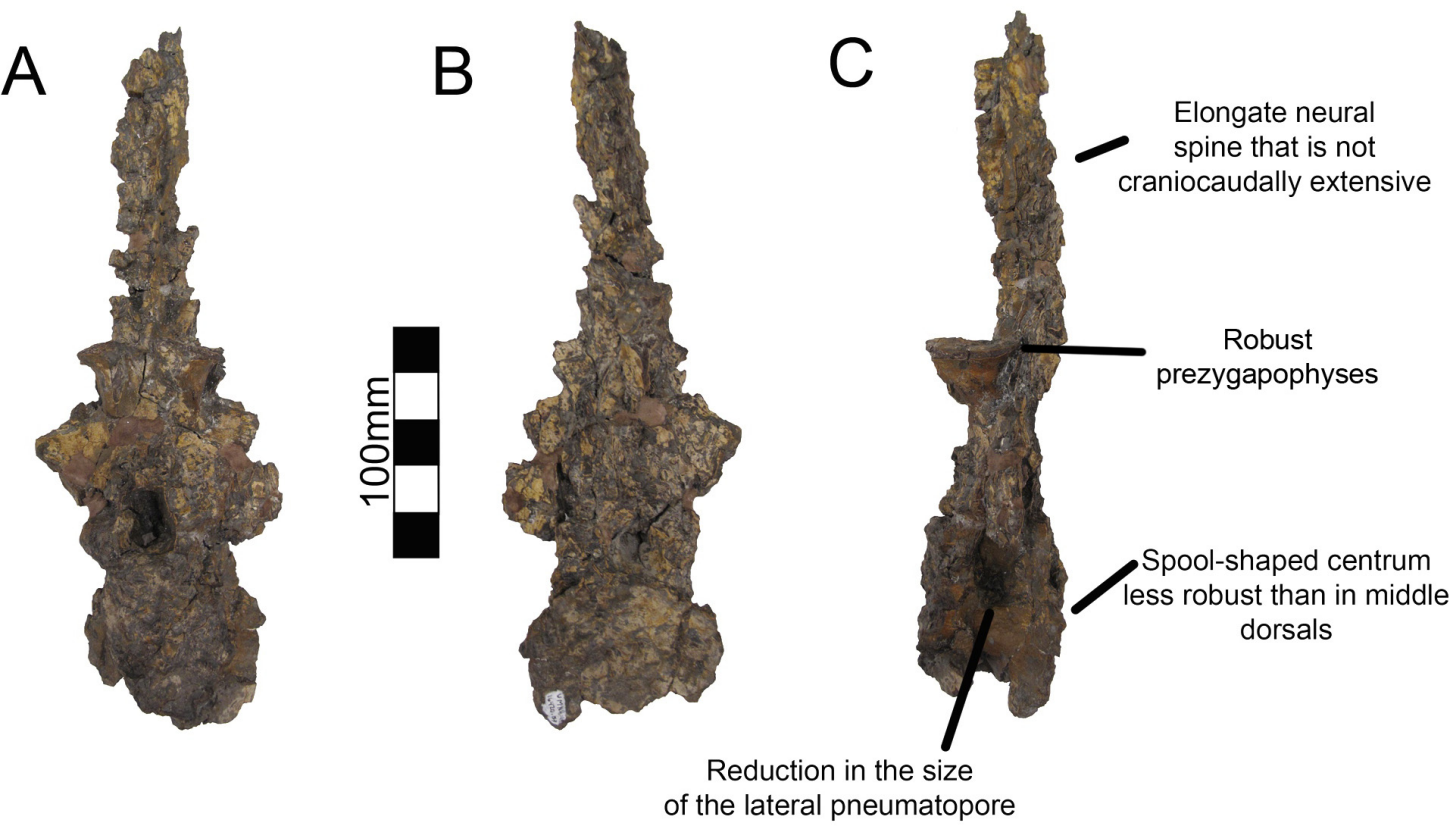
*Nothronychus* (UMNH VP16420) caudal dorsal vertebra. Caudal dorsal vertebra preserved in (A) cranial, (B) caudal, and (C) left lateral views. Figure explanations of figure. Scale = 100 mm.

There are two well-preserved mid-dorsal vertebrae in UMNH VP16420 (Fig 9). The centra are larger than that of dorsal one and are slightly amphicoelous, similar to other therizinosaurians (Fig 9). In the first mid-dorsal, the space between the centrum and the prezygapophyses is smaller than in more cranial dorsals, with the prezygapophyses located just dorsal to the neural canal and smaller centrodiapophyseal and centrozygapophyseal laminae (Fig 6). The second mid-dorsal possesses the same configuration, except that the prezygapophyses are slightly elevated. There are large pneumatopores on the dorsolateral surface of the centra as in dorsal one, larger than in *Falcarius*, *Neimongosaurus*, or *Suzhousaurus* (Fig 9) [14, 18, 38]. The neural spines are well developed attaining approximately the same height as the entirety of dorsal one as in *Neimongosaurus* (Fig 9) [14]. The neural spines are also craniocaudally wide in lateral view (Fig 9). No laminae are clearly preserved on either vertebra. There are three caudal dorsals in UMNH VP16420. They share reduced neural spines, robust centra, and robust pre- and postzygapophyses. The pneumatopore on the dorsolateral aspect of the centra is present on all dorsals, though it reduces in size along the column as in *Na*. *brevispinus* and *Suzhousaurus* (Fig 10) [7, 38]. The transverse processes are angled ventrally, but are broken on their ends so their complete lengths cannot be determined. No parapophyseal or spinal laminae are present on any dorsals, but the diagenetic distortion makes this difficult to discern with certitude, especially in the mid-dorsals. Wilson [39] remarked that spinal laminae are not characteristic of Saurischia, but are variably present. They do not appear to be present in other therizinosaurians.

#### Sacral Vertebrae

**Fig 11:**
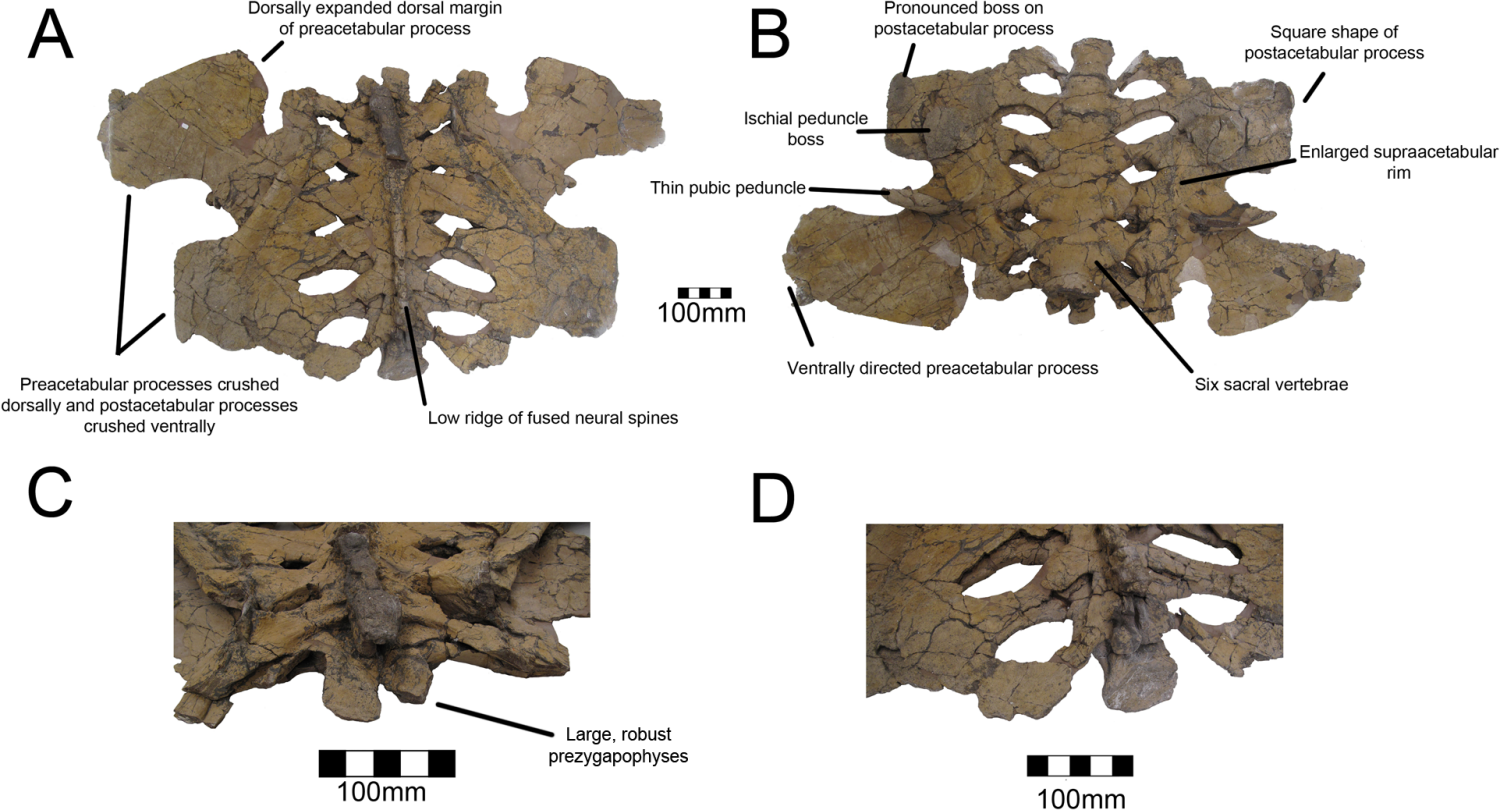
*Nothronychus* (UMNH VP16420) sacrum and ilium. Sacrum and ilia in (A) dorsal and (B) ventral view with ilia squashed laterally. (C) Cranial and (D) caudal views. Figure explanations on figure. Scale = 100 mm.

A complete sacrum is known for UMNH VP16420 (*No*. *graffami*) and no sacrals are known from MSM P2117 so the following description only applies to *No*. *graffami*. The entirety of the sacrum is crushed dorsoventrally making the vertebrae appear substantially wider than long unlike *Segnosaurus* [6]. It is possible that this is a diagenetic feature and additional materials are needed to confirm this as a trait. The six sacral vertebrae are completely fused, with only a trace of suture lines dividing them (Fig 11). Six sacral vertebrae is the general condition in therizinosaurians, though the basal form *Jianchangosaurus* has only five [30]. The number of sacral centra in *Falcarius* is unknown [18]. The centra have a spool shape, though they are not as constricted as the dorsal centra, and are larger than the caudal dorsal or proximal caudal centra, as in other therizinosaurians. The sacral ribs are robust and caudolaterally projecting. The first five are more robust than the final pair, which are smaller at their bases and then expand caudolaterally to connect to the ilia. The spaces between adjacent sacral ribs are also caudolaterally oriented, oblong in shape, and increase in size caudally; however this could be a diagenetic artifact associated with shearing. There is a pronounced continuous dorsal ridge along the sacral column composed of fused neural spines such that individual neural spines cannot be discerned. This is unlike the condition in *Segnosaurus*, in which the neural spines are distinguishable [6]. Both prezygapophyses are preserved on the first sacral vertebra. They are much more expanded and thickened in all aspects than the prezygapophyses on the dorsal or caudal vertebrae, though those on the caudal dorsals are approaching their size and robusticity.

#### Caudal Vertebrae

**Fig 12:**
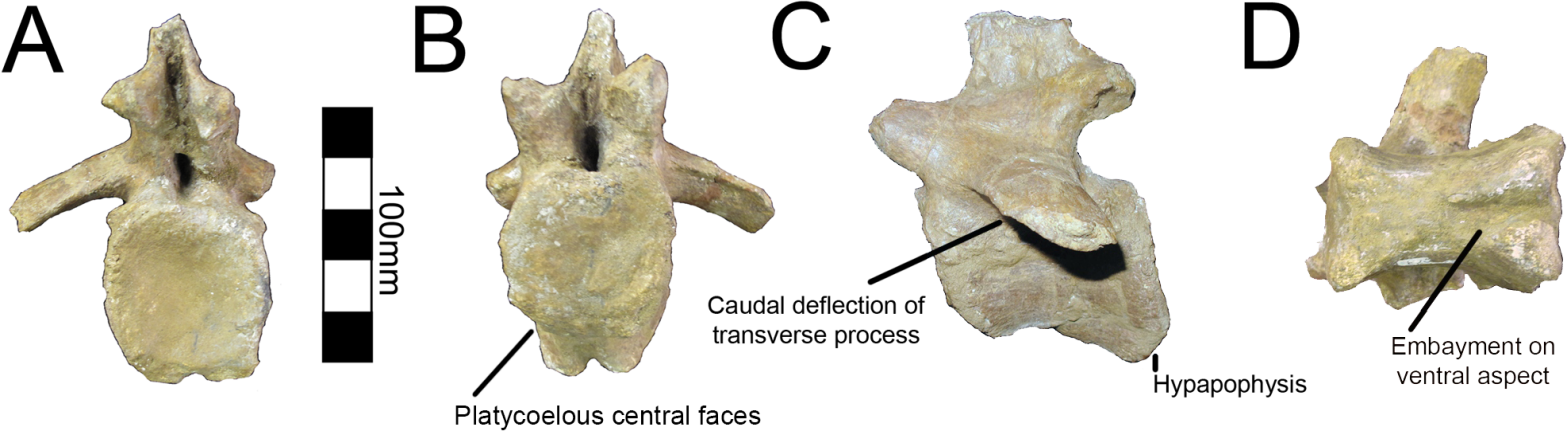
*Nothronychus* (MSM P2117) middle caudal vertebra. Single preserved middle cervical in (A) cranial, (B) caudal, (C) left lateral, and (D) ventral views. Scale = 100 mm.

One caudal vertebra is preserved with MSM P2117 (*No*. *mckinleyi*) and twenty are preserved with UMNH VP16420 (*No*. *graffami*), which appears to constitute a nearly complete tail (Fig 6). Caudal series are completely or nearly completely preserved in a number of therizinosaurian taxa in addition to *No*. *graffami*, including *Jianchangosaurus* (n = 11+), *Beipiaosaurus* (n = 30), *Alxasaurus* (n = 21), and *Neimongosaurus* (n = 22) [12, 14, 30, 40]. As in other elements, the majority of the caudal vertebrae attributed to UMNH VP16420 are craniocaudally or bilaterally compressed, though not to the degree of the dorsal vertebrae, while the caudal with MSM P2117 is well preserved and not obviously distorted (Fig 12). Based on the order found in UMNH VP16420, it would appear the caudal in MSM P2117 is a middle caudal [2] rather than a cranial caudal as suggested by Kirkland and Wolfe [16]. The description follows the series of UMNH VP16420. The vertebral order is not always evident, but the estimated order of the vertebrae presented here is numbered in S1 Table.

**Fig 13:**
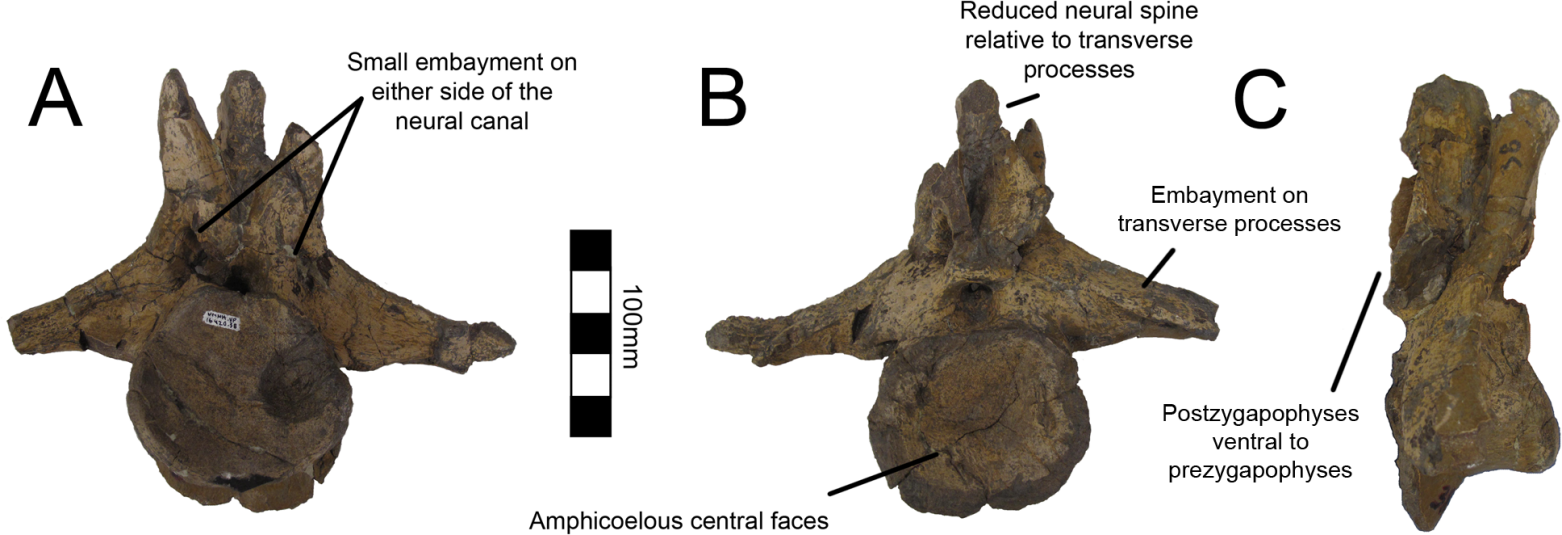
*Nothronychus* (UMNH VP16420) proximal caudal vertebra. Proximal caudal vertebra in (A) cranial, (B) caudal, and (C) right lateral views. Figure explanations on figure. Scale = 100 mm.

All caudal articular faces of the centra for both species are weakly concave as in *Falcarius*, *Neimongosaurus*, and *Erliansaurus* [14, 15, 18]. The two largest caudals of UMNH VP16420 are distinguishable as the proximal caudals, though it is not clear which of the two is the first and which is second. Neither vertebra clearly articulates with the sacrum. In the first two proximal caudals, the dorsal surfaces of the transverse processes have a distinct embayment, whereas the ventral surfaces have a weak buttress. The dorsal base of the transverse processes of caudal one has a small pleurocoel on both the cranial and caudal surfaces (Fig 13). There is a small pleurocoel on the dorsolateral aspect of the centra in the proximal and middle caudals as in *Erliansaurus* [15]. This is unlike *Neimongosaurus*, which does not have pleurocoels after the first few caudals [14]. Additionally, the neural spine is quite low and close to the centrum (Fig 13), as in *Segnosaurus* [6]. The neural spine is shorter than the transverse processes as in *Neimongosaurus* [14]. In caudal two, the zygapophyses are preserved demonstrating that the postzygapophyses are located ventral to the prezygapophyses and are less caudally extensive than the prezygapophyses are cranially. On caudal three, the tops of the transverse processes are flat rather than embayed as in caudals one and two. The pleurocoels located ventral to the transverse processes are less pronounced than in either caudal one or two. Caudal four is similar to caudal three, except that its features are reduced.

**Fig 14:**
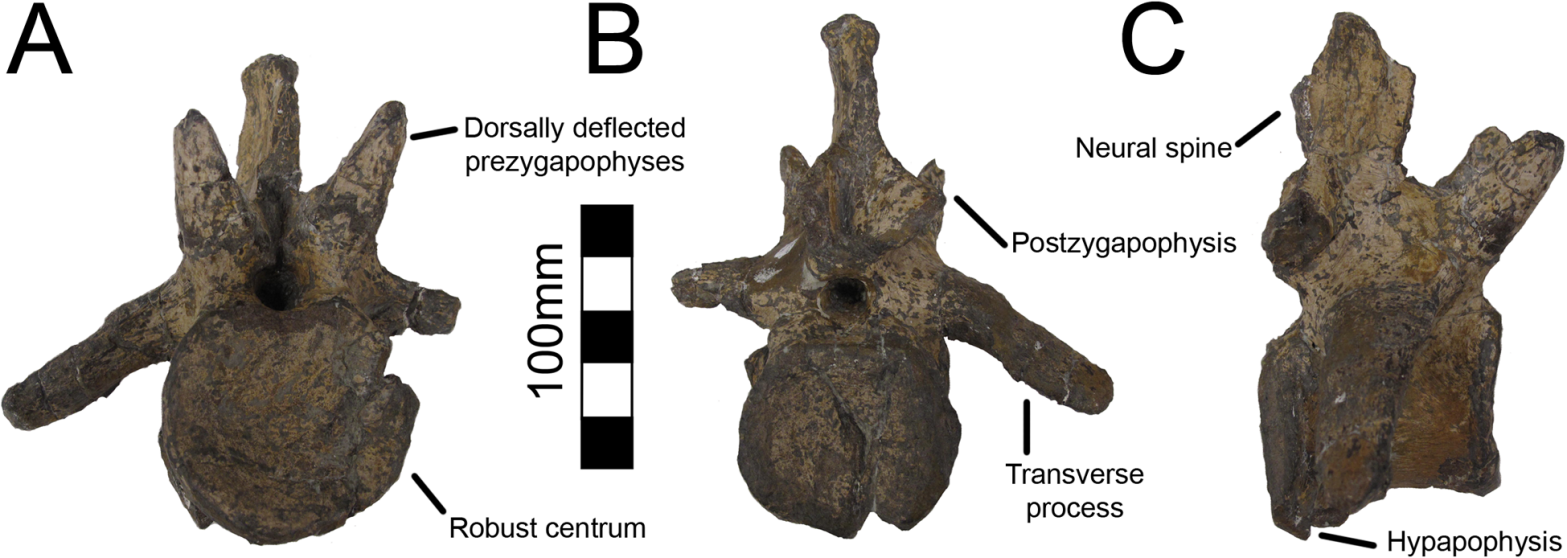
*Nothronychus* (UMNH VP16420) middle caudal vertebra. Middle caudal vertebra in (A) cranial, (B) caudal, and (C) right lateral views. Scale = 100 mm.

Caudal five is substantially smaller than caudal four so much so that there may be several missing caudals between caudal four and five. The centrum, transverse processes, and postzygapophyses are all smaller and less robust (Fig 14). A small ventral keel appears at the level of caudal five and continues caudally. Caudal five is also the first caudal with evident chevron facets. Caudal six preserves prezygapophyses whereas caudal five did not. They are approximately the same size as in caudal four, though the entirety of the vertebra is smaller suggesting this was also the case in caudal five. Caudals eight through twelve all have similar features to caudals five and six with a decrease in size and robusticity. This is similar to the caudal preserved with MSM P2117 and is probably between nine and eleven in the column. However, the caudal preserved with MSM P2117 is platycoelous unlike those of *No*. *graffami* (Fig 12). There is also a caudoventral heel on the MSM P2117 caudal that is much more expansive than those of UMNH VP16420. Both of these features are used to distinguish the species.

**Fig 15:**
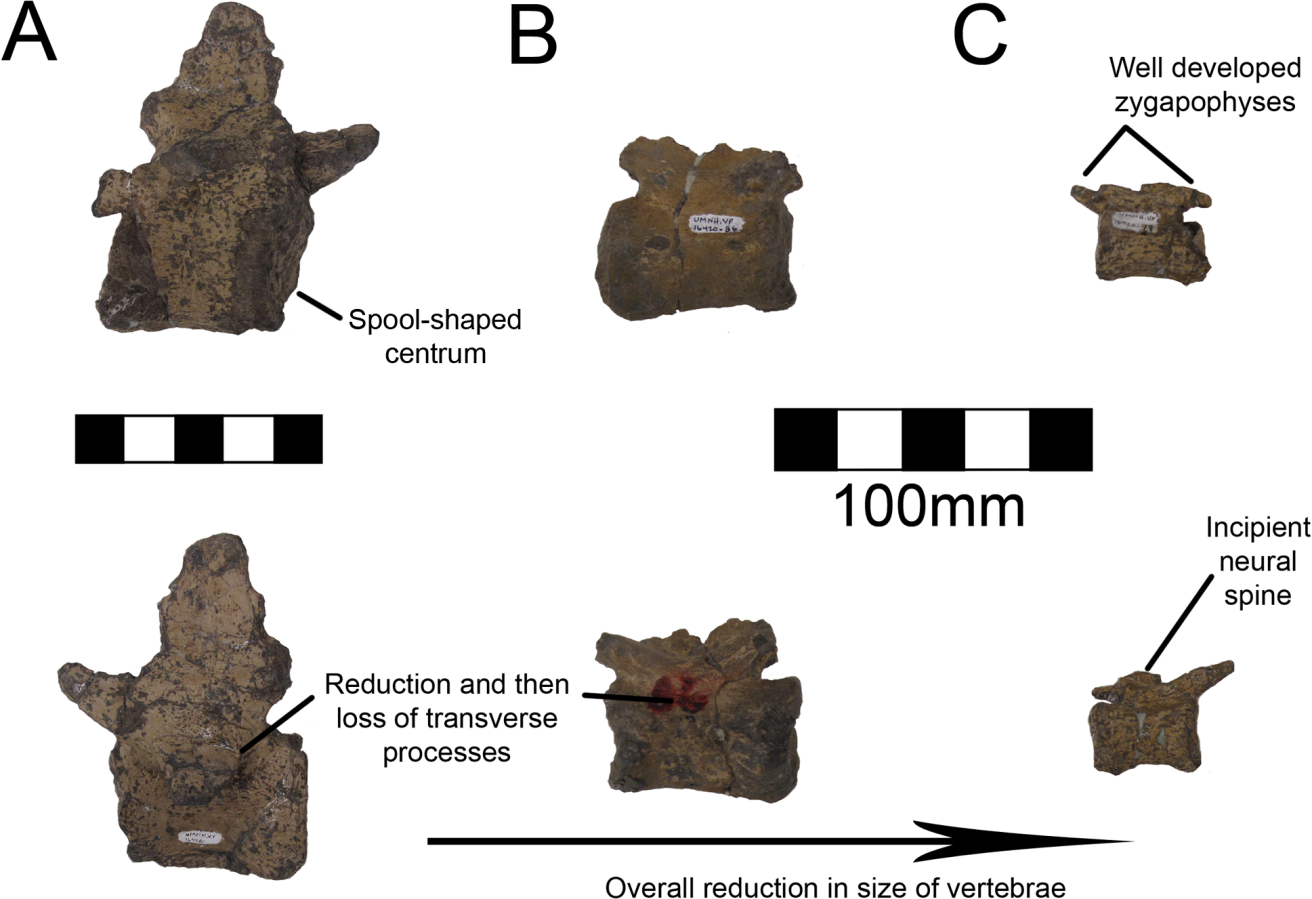
*Nothronychus* (UMNH VP16420) distal caudal vertebra. (A) More proximal distal caudal vertebra maintaining a large neural spine and transverse processes. Scale = 100 mm. (B) More distal distal caudal vertebrae and (C) distalmost caudal. Scale = 100 mm. Top figures are in left lateral view and bottom figures are in right lateral view.

Caudal thirteen has a spool-shaped centrum morphology in comparison with the boxier cranial caudals (Fig 15A). The prezygapophyses are much smaller and the chevron facets are incipient. Caudal fourteen shares this morphology and is the last vertebra with transverse processes. The final caudal with transverse processes is number thirteen in *Alxasaurus* [12]. Caudals fifteen and sixteen have a more exaggerated spool-shaped morphology whereby the centra are much longer than tall. They are more abbreviated craniocaudally than in *Falcarius* and are similar to other therizinosaurians. Caudal seventeen is much smaller than caudal fifteen and it is possible that several caudals between sixteen and seventeen were not preserved. The prezygapophyses are elongate extending cranially over more than half the preceding centrum. Caudals eighteen through twenty all share this morphology and become progressively smaller and less robust (Fig 15B and 15C). Although it is possible caudal twenty is the final caudal based on comparisons with other therizinosaurians [12, 14, 18, 40], it is also possible that there were several more caudals further distally. There is no evidence for a pygostyle-like structure as seen in *Beipiaosaurus* [40].

#### Chevrons

A number of proximal and middle caudal chevrons are preserved with UMNH VP16420 (*No*. *graffami*) and many can be articulated positively with their respective caudal vertebra (S2 Fig). Chevrons are known for *Falcarius*, *Alxasaurus*, and *Neimongosaurus*. No distal chevrons are known for UMNH VP16420. The chevrons decrease in size caudally. *Falcarius* has a cranial tuberosity on the chevrons that becomes more pronounced distally [18], but this cranial tuberosity is not found in UMNH VP16420 or *Neimongosaurus* [14]. The chevrons in UMNH VP16420 have straight shafts similar to *Neimongosaurus* [14], unlike the caudally deflected shafts of *Falcarius* [18]. The chevrons articulate with both of their respective caudal centra equally, as in *Falcarius* [18]. The haemal canal is ovular rather than subtriangular. The chevrons of UMNH VP16420 more closely resemble those of *Neimongosaurus* than *Falcarius* or *Alxasaurus* suggesting a change from caudally deflected chevrons in less specialized taxa to chevrons with straighter shafts through therizinosaurian evolution.

#### Ribs and gastralia

**Fig 16:**
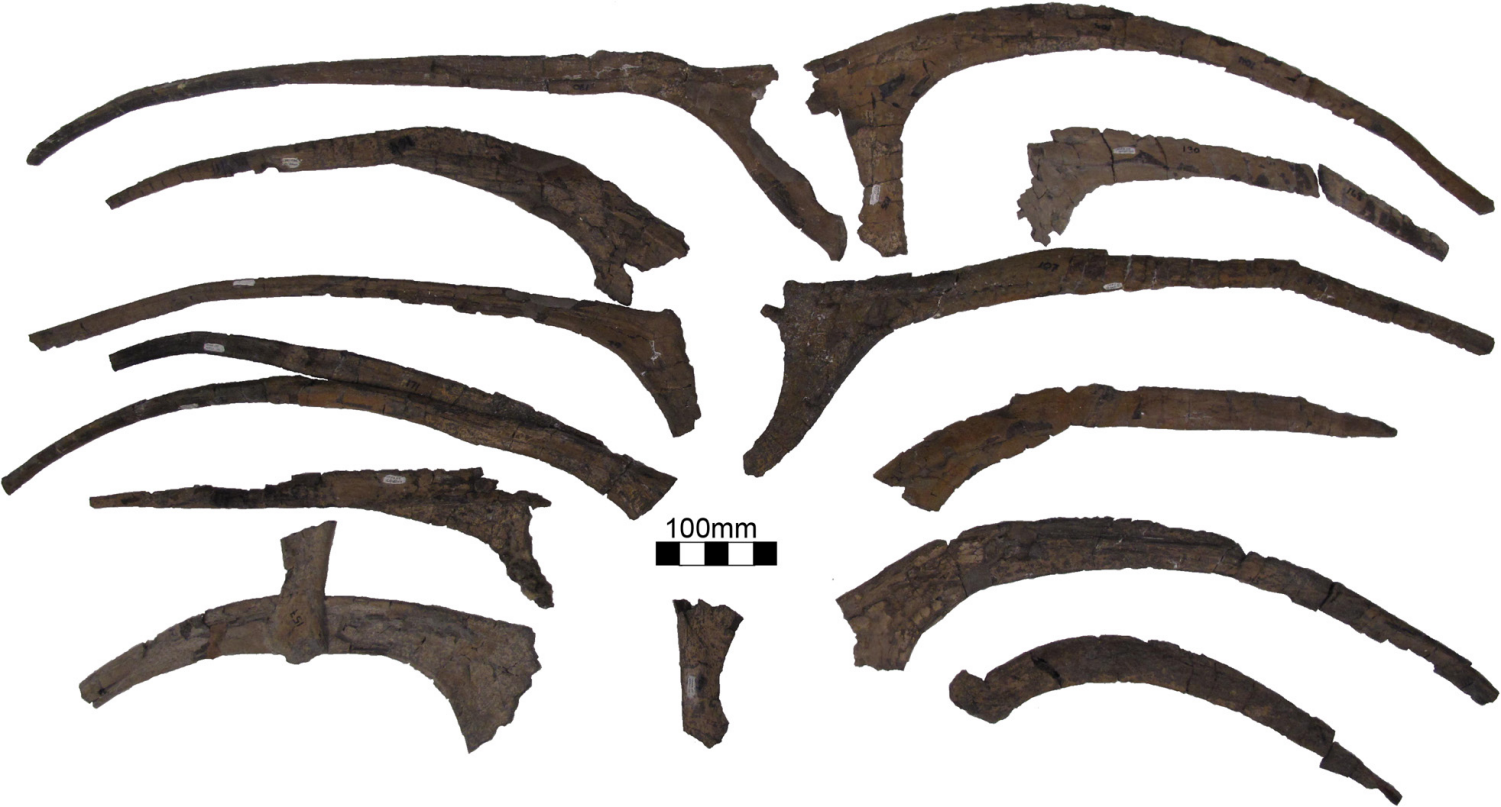
*Nothronychus* (UMNH VP16420) ribs. Large number of ribs preserved with UMNH VP16420. Scale = 100 mm.

UMNH VP16420 (*No*. *graffami*) preserves a large number of dorsal ribs (14 or more) as well as gastralia (Fig 16). These are disarticulated so it is not possible to associate particular ribs or gastralia with vertebrae except by inference using robusticity as a guide. The ribs vary in length substantially such that the largest rib is more than twice the length of the smallest. The largest ribs have an offset capitulum that is well developed and nearly as robust as the rib itself. In the largest ribs, the capitulum can be up to one quarter the total length of the rib. MSM P2117 (*No*. *mckinleyi*) preserves two gastralia. Kirkland and Wolfe [16] misidentified a small gastral element as a furculum. However, it appears too large to be a furculum based on the preserved element in UMNH VP16420 and is parsimoniously considered a gastral element. Both gastralia are crescent shaped in MSM P2117 and similar to those preserved in UMNH VP16420 (S3 Fig).

#### Scapula

**Fig 17:**
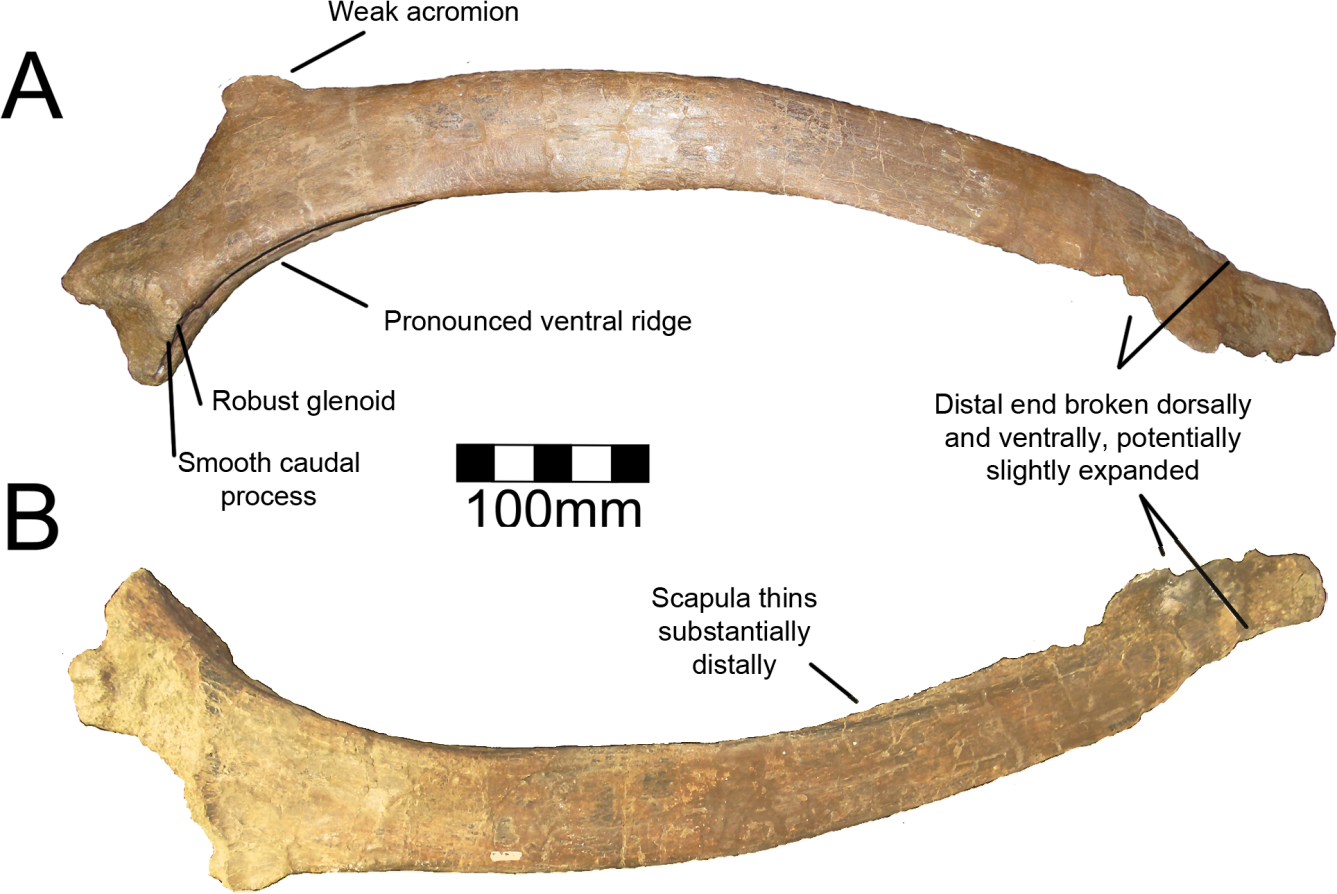
*Nothronychus* (MSM P2117) left scapula. Left scapula in (A) lateral and (B) medial views. Figure explanations on figure. Scale = 100 mm.

**Fig 18:**
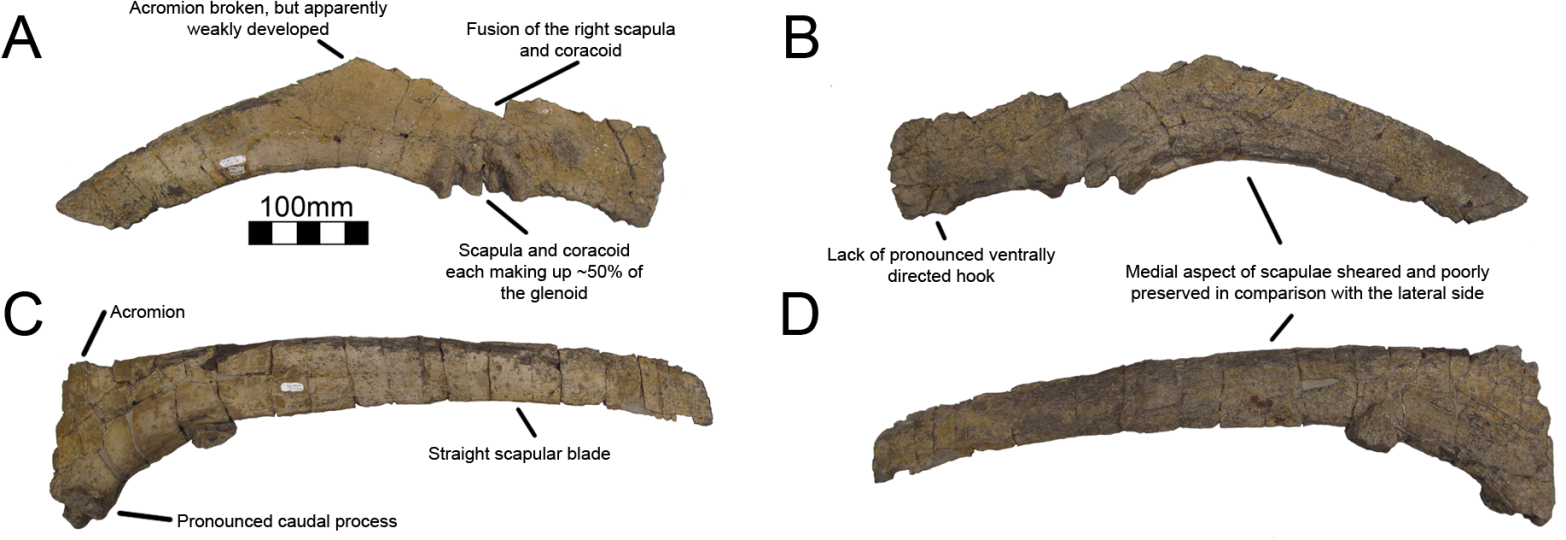
*Nothronychus* (UMNH VP16420) scapulae. Partial right proximal scapula and ventral coracoid in (A) lateral and (B) medial views. Left lateral scapula in (A) lateral and (B) medial views. Figure explanations on figure. Scale = 100 mm.

The right scapula of MSM P2117 (*No*. *mckinleyi*) is nearly complete, missing its distal end and the proximodorsal acromial region (Fig 17). Both scapulae are known for UMNH VP16420 (*No*. *graffami*) and they preserve aspects of the scapula not preserved in MSM P2117, yet are themselves fragmentary. The right scapula of UMNH VP16420 is missing most of the scapular blade and the acromion immediately dorsal to the glenoid, yet preserves the scapulocoracoid suture and is fused to a partial coracoid (Fig 18A and 18B). The left scapula of UMNH VP16420 preserves most of the scapular blade and most of the acromion (Fig 18C and 18D). The medial aspects of both scapulae of UMNH VP16420 have been sheared off and have been weathered, but the lateral aspects are well preserved. MSM P2117 preserves the medial aspect making the two specimens excellent complements.

Kirkland and Wolfe [16] describe the proximal scapula of MSM P2117 as likely not having an expanded acromion (Fig 17A). The acromial region is mostly missing in MSM P2117. The acromion of the left scapula of UMNH VP16420 is broken along the dorsal edge where the bone thins substantially. It appears that the acromion is mostly preserved in the left scapula of UMNH VP16420, but this will have to be confirmed by future finds. The acromion is expansive in all other therizinosaurian taxa that preserve this feature including *Falcarius*, *Jianchangosaurus*, *Beipiaosaurus*, *Suzhousaurus*, *Neimongosaurus*, and *Therizinosaurus* [4, 14, 17, 30, 35, 38]. The acromion of less specialized therizinosaurians such as *Falcarius* is much better developed than what is preserved in *Nothronychus* [17, 30]. The majority of the acromion is also missing in *Falcarius* specimens, but the angulation for the acromion is nearly 90° demonstrating the extreme expansion in comparison to *Nothronychus*. *Jianchangosaurus* has a reduced acromion in comparison with *Falcarius*, though the angle of the acromion is still near 90° showing that it is more developed than in *Nothronychus* [30]. A reduced acromion is found in ornithomimids [41] and oviraptorosaurs [42], which bracket therizinosaurians phylogenetically [2]. A more enlarged acromion is found in non-maniraptoriform theropods and basal avian forms.

Though the coracoid is not preserved in MSM P2117, it is evident based on the scapula that the scapula contributes 50% of the glenoid as in other therizinosaurians. The glenoid is the mediolaterally thickest aspect of the scapula (Fig 17). UMNH VP16420 has a pronounced caudal buttress on the caudal aspect of the glenoid fossa, a feature distinguishing *No*. *graffami* from *No*. *mckinleyi*. The corresponding area on MSM P2117 is flattened and reduced (Fig 17). The ventral aspect of the scapula is thickened for the proximal 30% of the scapular blade. Along the proximal third of the scapula, there is a prominent ventral groove in MSM P2117 (Fig 17). This is incipient in UMNH VP16420, but this is likely due to poor preservation of the medial aspect of both scapulae in UMNH VP16420 (Fig 18). This groove diminishes for 130 mm in the middle of the scapula and then is reestablished on the distal end. The scapula is much thicker ventrally than dorsally along the entire blade. There is a small, rugose tubercle on the lateral side of the ventral edge of the scapula along the groove on MSM P2117. This is likely the origin point for the m. triceps longus [43]. A rugose point is homologous with this groove in basal forms (*Falcarius*, *Jianchangosaurus*, *Beipiaosaurus*). The region in *Suzhousaurus* is much more robust, but also has a groove likely as an origin for the m. triceps longus.

The blade of the scapula is very straight as is characteristic for therizinosaurians except *Falcarius*. The scapula was noted as tapering distally in MSM P2117 [2, 16]. This is considered a derived character for therizinosaurians [17] and has been noted in *Therizinosaurus* [4]. However, the distal blades in *No*. *mckinleyi* and *No*. *graffami* are broken both ventrally and dorsally (Figs 17 and 18). The dorsal aspect of the distal blade is broken below the top of the scapular blade and shifted ventrally, thus this region may not have expanded dorsoventrally. The ventral aspect of MSM P2117 shows some bone expanding below the ventral border of the scapula suggesting distal expansion along the ventral border rather than a truly tapering blade. Given that *Falcarius*, *Jianchangosaurus*, *Alxasaurus*, *Suzhousaurus*, and *Neimongosaurus* have slightly expanding distal scapular blades [12, 14, 17, 30, 38], it is possible that the apparent tapering of the blade in *Nothronychus* is a preservational artifact.

#### Coracoid

**Fig 19:**
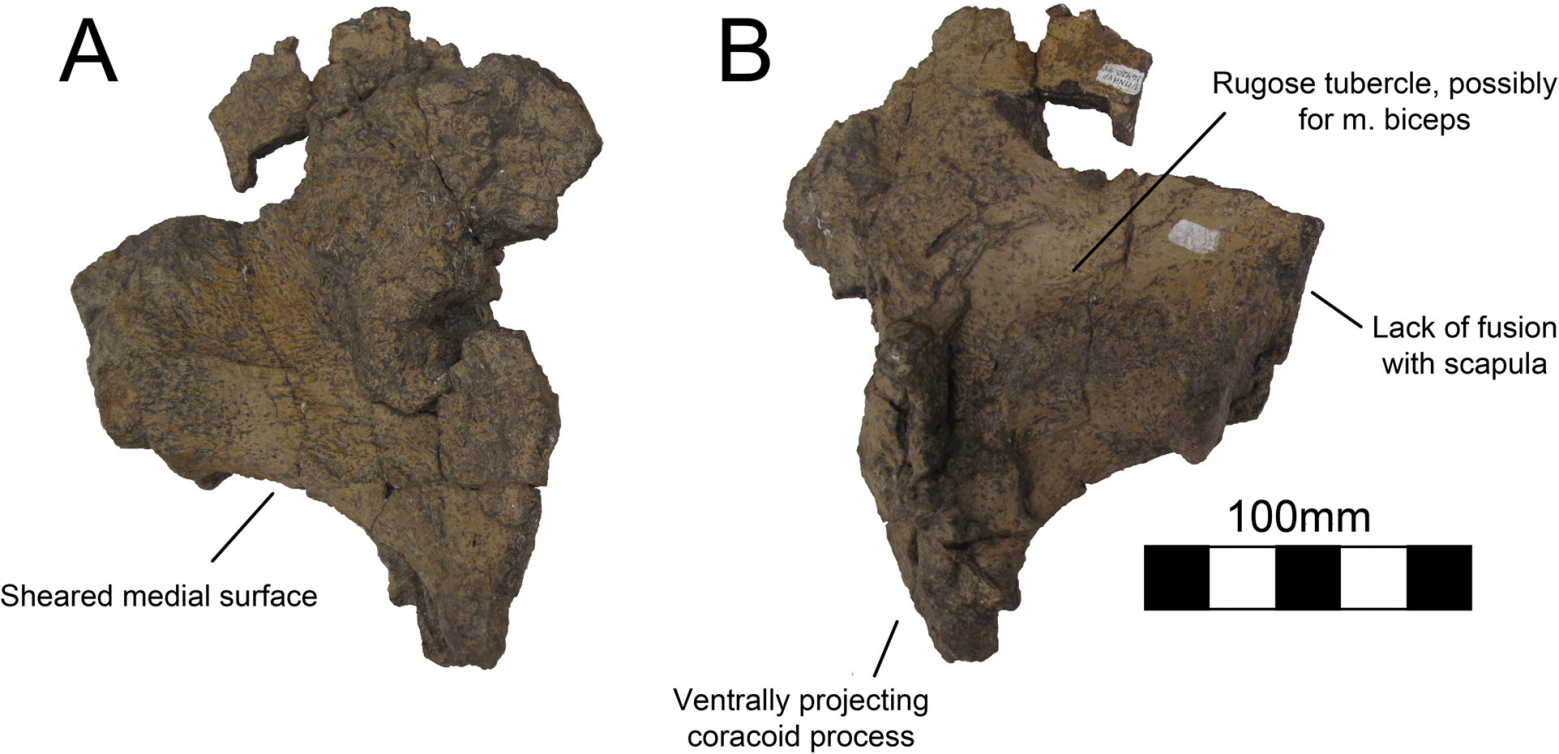
*Nothronychus* (UMNH VP16420) coracoid. Partial left coracoid in (A) medial and (B) lateral view. Figure explanations on figure. Scale = 100 mm.

Both coracoids are known for UMNH VP16420 (*No*. *graffami*), a partial right coracoid and a nearly complete left coracoid (Figs 18A, 18B and 19). Neither coracoid is known for MSM P2117 so this description only applies to *No*. *graffami*. Strangely, these two coracoids have very different morphology on their ventral margins. The ventral margin of the right coracoid is flattened, coming to a blunt coracoid process lacking a pronounced ventral projection (Fig 18A and 18B). However, the ventral margin of the left coracoid is strongly concave rather than flattened, and comes to a sharp, ventrally projecting coracoid process (Fig 19). It is possible that one of the coracoids is pathological, since it does not seem clear how diagenetic alteration would have affected either of these bones to this degree. However, it is not presently clear which of the two, if either, is pathologic.

Based on the preserved acromial regions of the scapulae of UMNH VP16420 and the fused scapulocoracoids of MSM P2117 (*No*. *mckinleyi*), the dorsal aspect of the coracoids of UMNH VP16420 is sheared parallel to the long axis of the scapulocoracoids. The more complete left coracoid indicates that *No*. *graffami* had similar coracoid proportions to other therizinosaurians. The sutural line is nearly obliterated on the medial and lateral aspects of the scapulocoracoid and has a faint cranial orientation on the right side, though the left scapulocoracoid is unfused demonstrating variation in fusion within an individual [2]. The suture appears most obviously in the glenoid region and the glenoid is made up of approximately 50% of the scapula and 50% of the coracoid. The coracoid foramen is not preserved on either side, but is presumed incipient as in other therizinosaurians.

Other therizinosaurians that have complete or partial coracoids include: *Falcarius* [17], *Beipiaosaurus* [13], *Alxasaurus* [12], *Suzhousaurus* [38], *Neimongosaurus* [14], and *Therizinosaurus* [4]. The coracoid of *No*. *graffami* is characteristic as it possesses a strongly concave hook-shaped coracoid process on the left side as is found in ornithomimosaurs [41], oviraptorosaurs [42], and other therizinosaurians, while the ventral process is absent on the right side. Therefore it is most likely that the left coracoid is characteristic of the true shape and the right coracoid is aberrant for *No*. *graffami*. The cranioventral aspect of the right coracoid of UMNH VP16420 does have a break, but the missing region is likely minor and would not support an extensive hooked process. The coracoid process in *Segnosaurus* is much less developed than in other therizinosaurians [6], but the coracoid is also much shorter craniocaudally than in UMNH VP16420 making the two easily distinguishable.

#### Humerus

**Fig 20:**
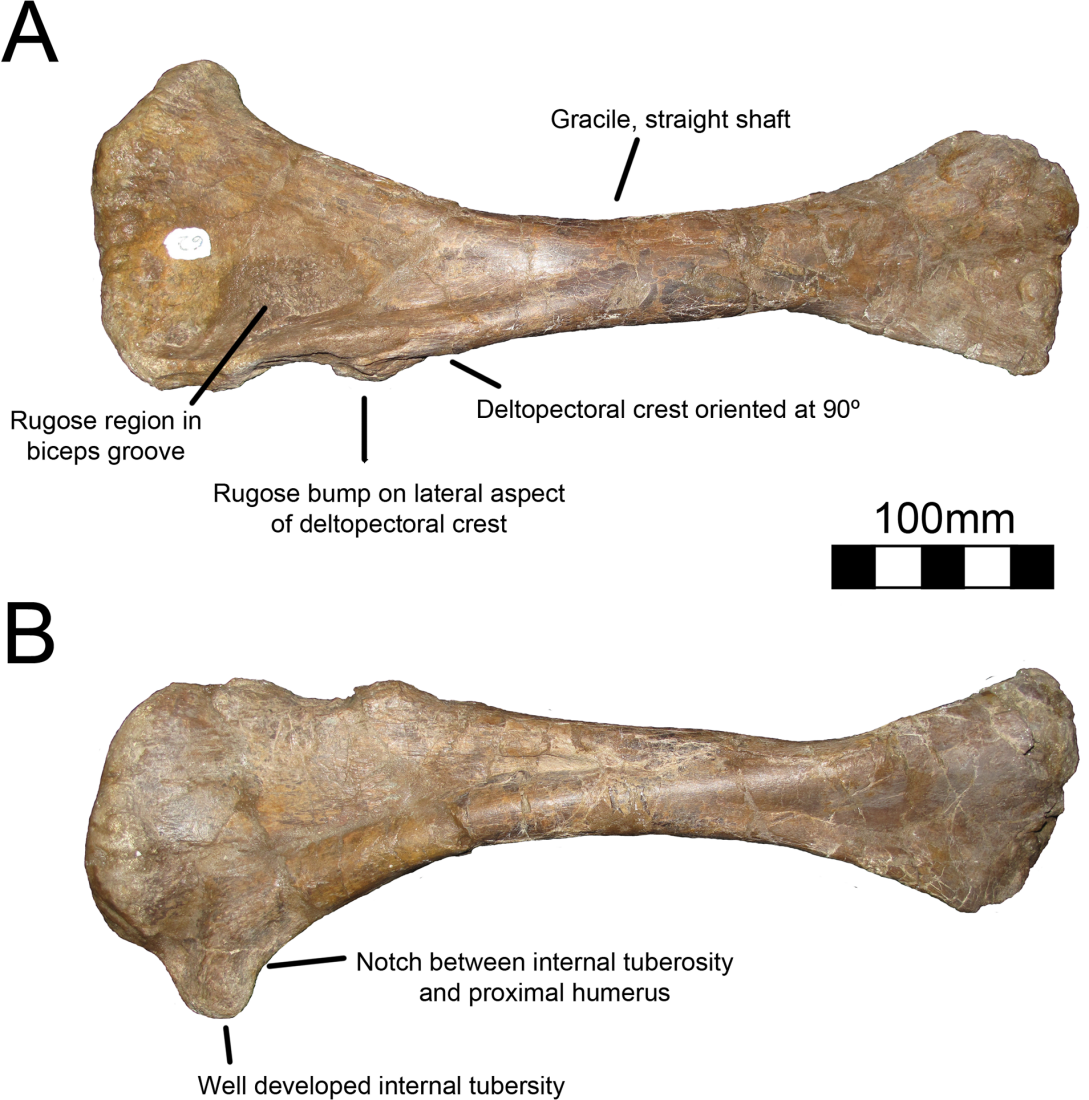
*Nothronychus* (MSM P2117) right humerus. Complete right humerus in (A) cranial and (B) caudal views. Figure explanations on figure. Scale = 100 mm.

**Fig 21:**
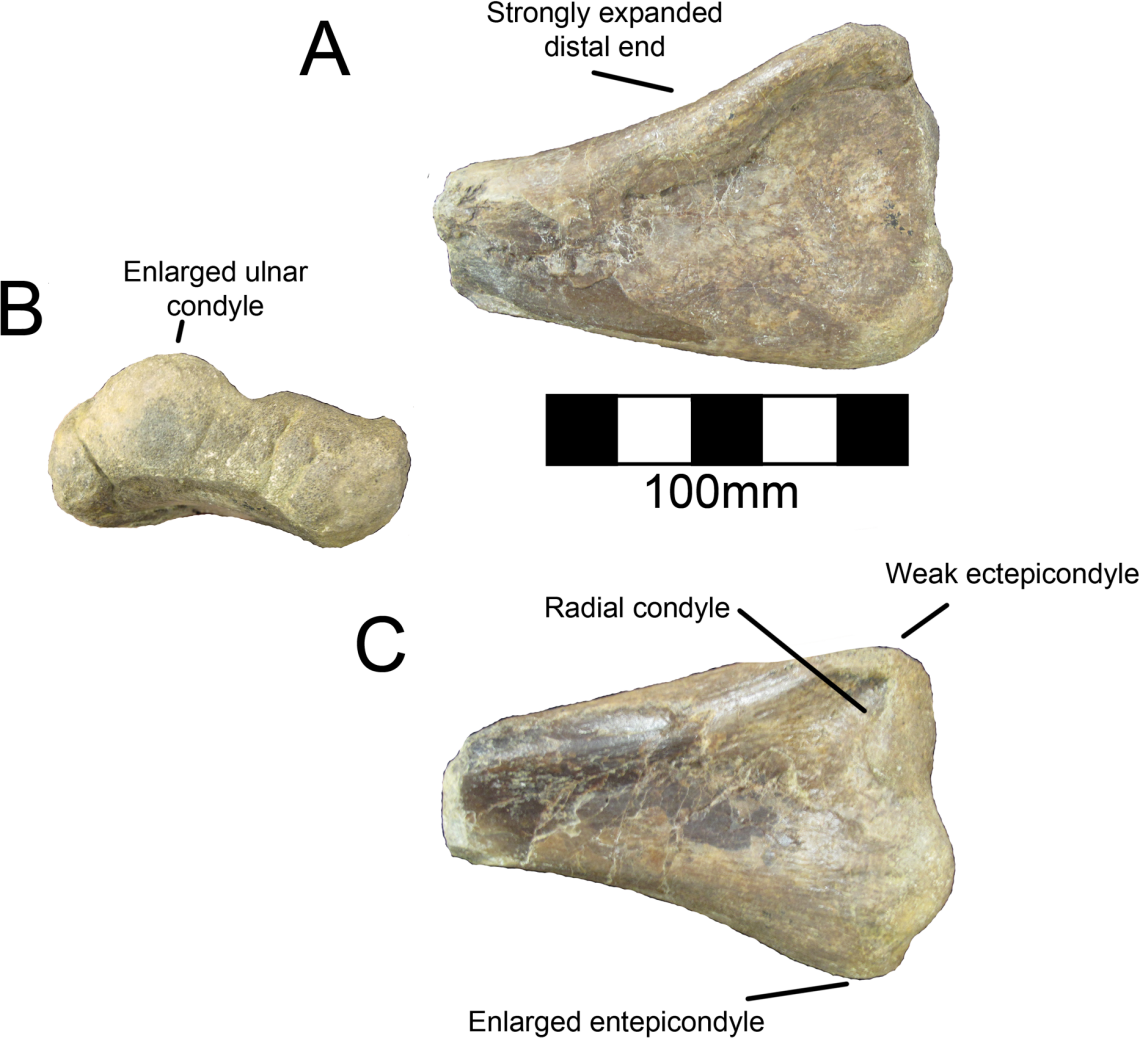
*Nothronychus* (MSM P2117) left humerus. Partial distal left humerus in (A) caudal, (B) distal, and (C) cranial views. Figure explanations on figure. Scale = 100 mm.

**Fig 22:**
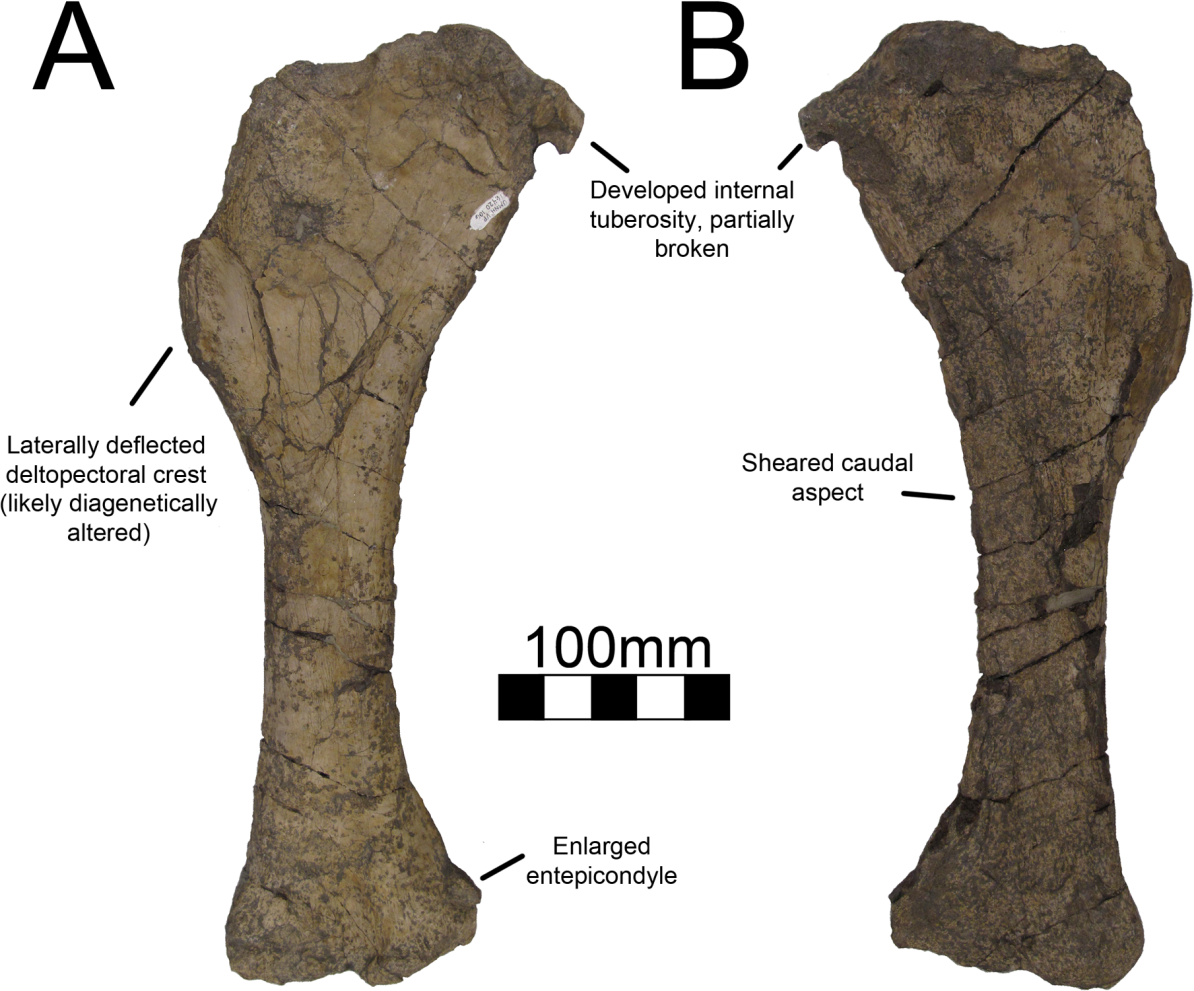
*Nothronychus* (UMNH VP16420) right humerus. Right humerus in (A) cranial and (B) caudal views. Figure explanations on figure. Scale = 100 mm.

The right humerus of MSM P2117 (*No*. *mckinleyi*) is completely preserved although the distal end is craniocaudally compressed (Fig 20). The left humerus preserves only the distal end, but it is undistorted (Fig 21). UMNH VP16420 (*No*. *graffami*) preserves both humeri in their entirety, but they are craniocaudally compressed and the caudal surface is sheared (Fig 22). A hypertrophied internal tuberosity is a characteristic trait of therizinosauroids and is present in both species of *Nothronychus* [18]. The internal tuberosity in both species of *Nothronychus* most resembles *Suzhousaurus* [38] while *Falcarius* and *Jianchangosaurus* have relatively smaller internal tuberosities (although still well developed) [17, 30] and *Neimongosaurus* and *Erliansaurus* have relatively larger internal tuberosities [14, 15]. *Therizinosaurus* has a relatively smaller internal tuberosity than other therizinosaurids. Both *Nothronychus* specimens have a depression between the head of the humerus and the internal tuberosity both on the proximal and caudal sides [2]. *Falcarius* has a small tuberosity lateral to the humeral head [17] that is not seen in either species of *Nothronychus*.

The humerus of MSM P2117 is more gracile than that of UMNH VP16420 and has a longer shaft relative to the length of the deltopectoral crest. Both humeri are gracile and elongate in comparison with other therizinosaurids and resemble *Suzhousaurus*. The proximal and distal ends are not substantially twisted in either species and resemble that of other therizinosaurians [2, 18]. UMNH VP16420 has a deltopectoral crest extending 42–45% of the length of the humerus (Fig 22) [2]. The length of the deltopectoral crest is approximately 129 mm in MSM P2117, making it 30–32% the length of the humerus (Fig 20). *Neimongosaurus*, *Erlikosaurus*, and *Falcarius* have deltopectoral crests with approximately the same proportions as MSM P2117 [14, 15, 17]. The deltopectoral crest of the right humerus of UMNH VP16420 is compressed laterally and the deltopectoral crest of the left humerus is compressed medially. It is possible that the deformation of the humerus has altered the relative length of the deltopectoral crest making it anomalously elongate; however, this seems unlikely. It would appear that the difference in the extent of the deltopectoral crest is a feature separating the two species of *Nothronychus*. The deltopectoral crest in MSM P2117 has a prominent lip oriented cranially at about 90° as in *Falcarius*, *Jianchangosaurus*, *Neimongosaurus*, and *Erliansaurus* (Fig 20) [14, 15, 17, 30]. The biceps groove medial to the deltopectoral crest is highly rugose in MSM P2117 (Fig 20). There is a distinct tuberosity on the lateral aspect of the deltopectoral crest in MSM P2117 (Fig 20) that is not present in UMNH VP16420 (Fig 22), though there is marked rugosity in this region. This tuberosity is not found in other therizinosauroids and may represent pathology of the insertion of the m. deltoideus.

The distal end of the right humerus of MSM P2117 and both humeri of UMNH VP16420 are crushed. Here we use the left humerus of MSM P2117, which is undeformed, for description. The radial condyle is mediolaterally larger than the ulnar condyle, but dorsoventrally smaller, and both have been rotated onto the cranial aspect of the humerus, as in therizinosauroids generally [4, 14, 15, 17]. An alariform entepicondyle is also well developed in both species of *Nothronychus* (Figs 21 and 22) [2] as well as *Falcarius*, *Suzhousaurus*, and *Erliansaurus* [15, 17, 38], and in contrast to *Jianchangosaurus*, which has a reduced entepicondyle [30]. The craniolateral ridge present on the distal humerus of *Falcarius* is not present in either MSM P2117 or UMNH VP16420 and this region is instead rounded.

#### Ulna

**Fig 23:**
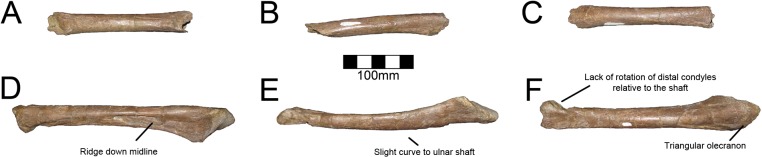
*Nothronychus* (MSM P2117) antebrachial element and right ulna. Antebrachium in multiple views (A, B, C). Right ulna in (D) cranial, (E) medial, and (F) caudal views. Figure explanations on figure. Scale = 100 mm.

**Fig 24:**
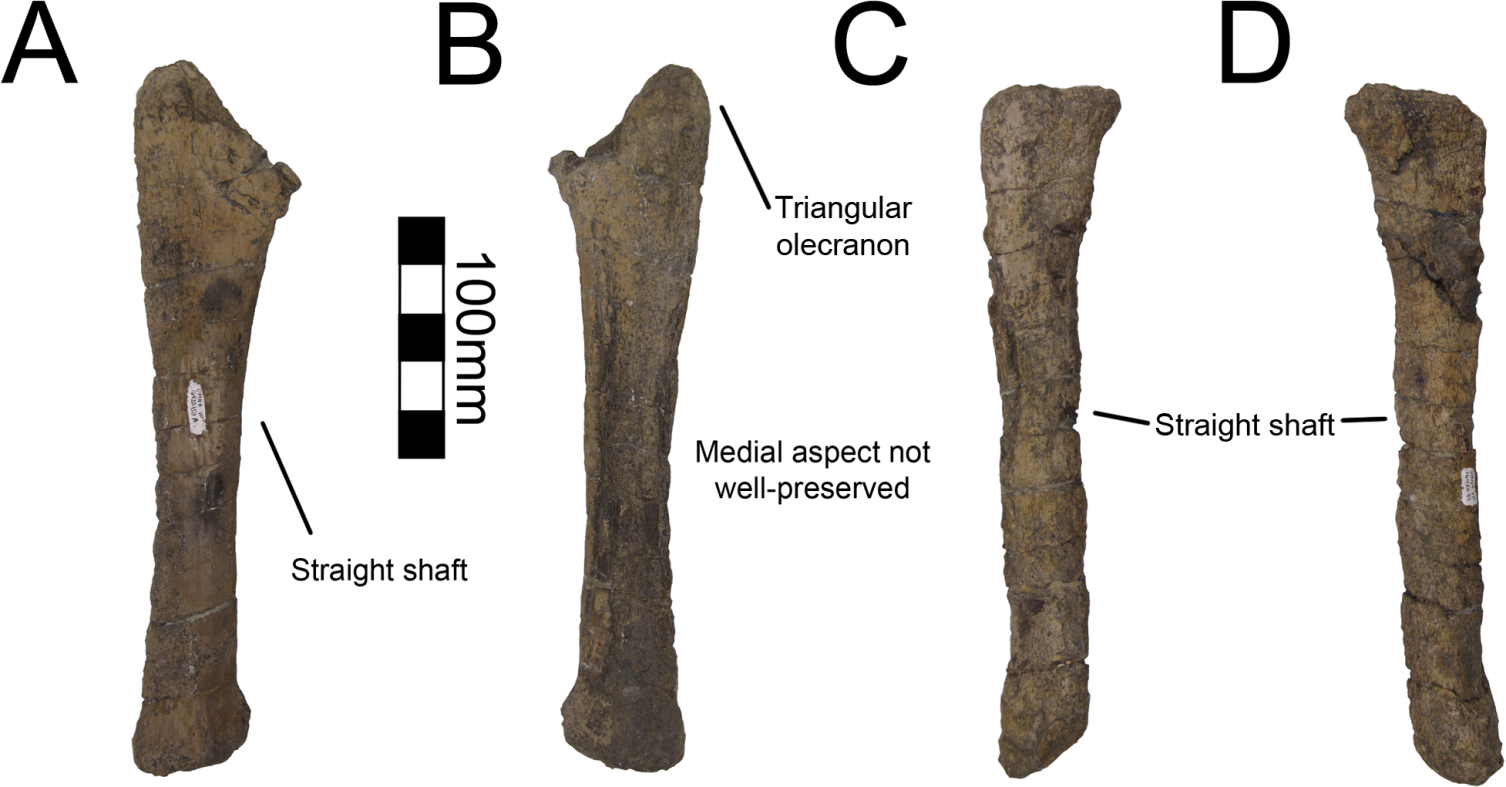
*Nothronychus* (UMNH VP16420) ulna and radius. Right ulna in (A) lateral and (B) medial views. Right radius in (C) lateral and (D) medial views. Figure explanations on figure. Scale = 100 mm.

Both ulnae are completely preserved for MSM P2117 (*No*. *mckinleyi*) and UNMH VP16420 (*No*. *graffami*) (Figs 23C, 23D, 23E, 24A and 24B). UMNH VP16420 has mediolaterally compressed ulnae that are missing cortical bone on the medial aspect. Considering the poor preservation of this element in UMNH VP16420, MSM P2117 is used here for description. The ulnae of MSM P2117 are slightly dorsally convex, especially proximally (Fig 23E), but not to the degree seen in non-therizinosaurian maniraptorans [25]. *Jianchangosaurus*, *Erliansaurus*, *No*. *graffami*, and *Therizinosaurus* have straight ulnar shafts [2, 4, 15, 30]. However, given the poor preservation of the ulnae, diagenetic distortion in UMNH VP16420 makes it difficult to assess the degree of curvature in *No*. *graffami* (Fig 24A). Though originally used as a synapomorphy distinguishing *No*. *mckinleyi* and *No*. *graffami*, we do not consider this feature to be definitive in UMNH VP16420. The caudal aspect is missing cortical bone surface on the distal end of both ulnae of MSM P2117 and UMNH VP16420. However, this does not seem to have affected the shape of the bone. The ulnae are 72–74% the length of the humeri in both MSM P2117 and UMNH VP16420. This is slightly lower than the ratio in *Falcarius* (77%) and *Jianchangosaurus* (78%) [17, 30]. The olecranon is triangular and expanded as in other therizinosaurians (Figs 23F and 24A). The medial distal condyle is expanded in comparison with the incipient lateral condyle on the right ulna (Fig 23F). However, the left ulna has subequal distal condyles. The condyles are not rotated as in *Falcarius* or *Erliansaurus* [15, 17] and are straight relative to the shaft as in *Therizinosaurus* [4].

#### Radius

Both radii are preserved in UMNH VP16420 (*No*. *graffami*) and are not substantially distorted though are missing some cortical bone surface (Fig 24C and 24D). Only the proximal half of the right radius is preserved; however, the left is complete. The shafts of the radii are straight, although as discussed for the ulnae, this may be related to diagenetic distortion in UMNH VP16420. Therizinosaurian radii are generally straight, but often have a slight sigmoid curve as in *Therizinosaurus* [4], *Neimongosaurus* [14], and *Falcarius* [17], unlike *Jianchangosaurus*, which has a completely straight radius [30]. The proximal end is rounded and ball-shaped and the distal end is flattened and cup-shaped. The proximal end has a facet for articulation with the ulna as in *Falcarius*. There is an incipient tubercle for the insertion of the m. biceps brachii and m. brachialis proximally, which may have been more pronounced, but are reduced due to poor preservation of the bone surface. Such a tubercle is present in *Neimongosaurus* [14], but not in *Falcarius* [17] or *Jianchangosaurus* [30].

A partial antebrachial element (MSM P4487) was found in the MSM P2117 (*No*. *mckinleyi*) quarry and consists solely of a fragment of the midshaft (Fig 23A, 23B and 23C). It has not been attributed to the holotype of *No*. *mckinleyi*, yet given that it is the same size and cross sectional shape as other antebrachial elements of MSM P2117, and no other theropod taxon is known from the site, it is likely referable to MSM P2117. The proximal aspect is triangular in cross-section, whereas the distal shaft is transversely compressed. The shaft is relatively straight. We interpret this as a fragment of the radius. However, both radii of UMNH VP16420 are crushed, therefore the cross-sectional shape of MSM P4487 cannot be compared to the radii attributed to *No*. *graffami*.

#### Metacarpals and manual phalanges

**Fig 25:**
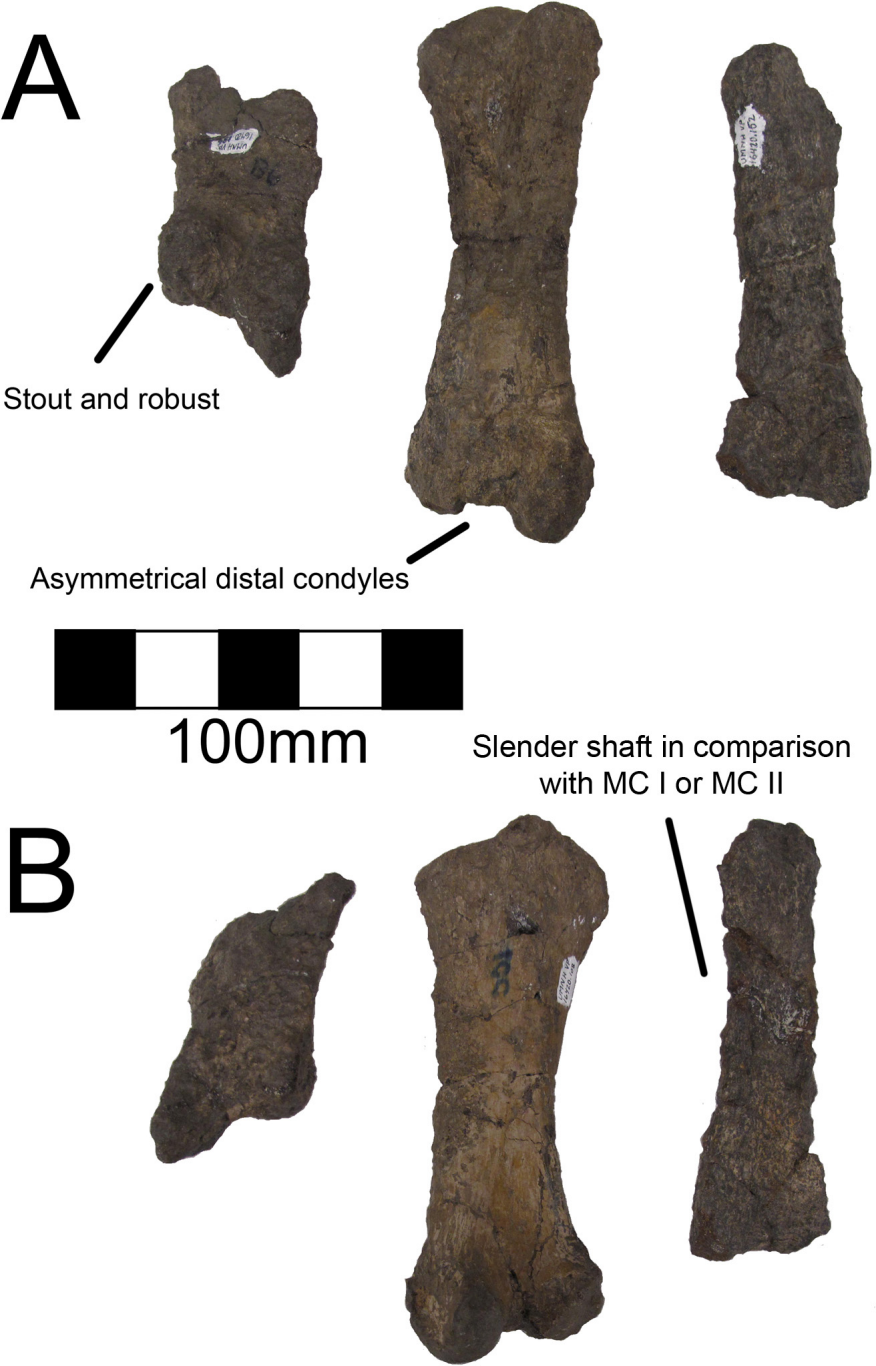
*Nothronychus* (UMNH VP16420) metacarpus. Metacarpus in (A) cranial and (B) caudal views. Figure explanations on figure. Scale = 100 mm.

**Fig 26:**
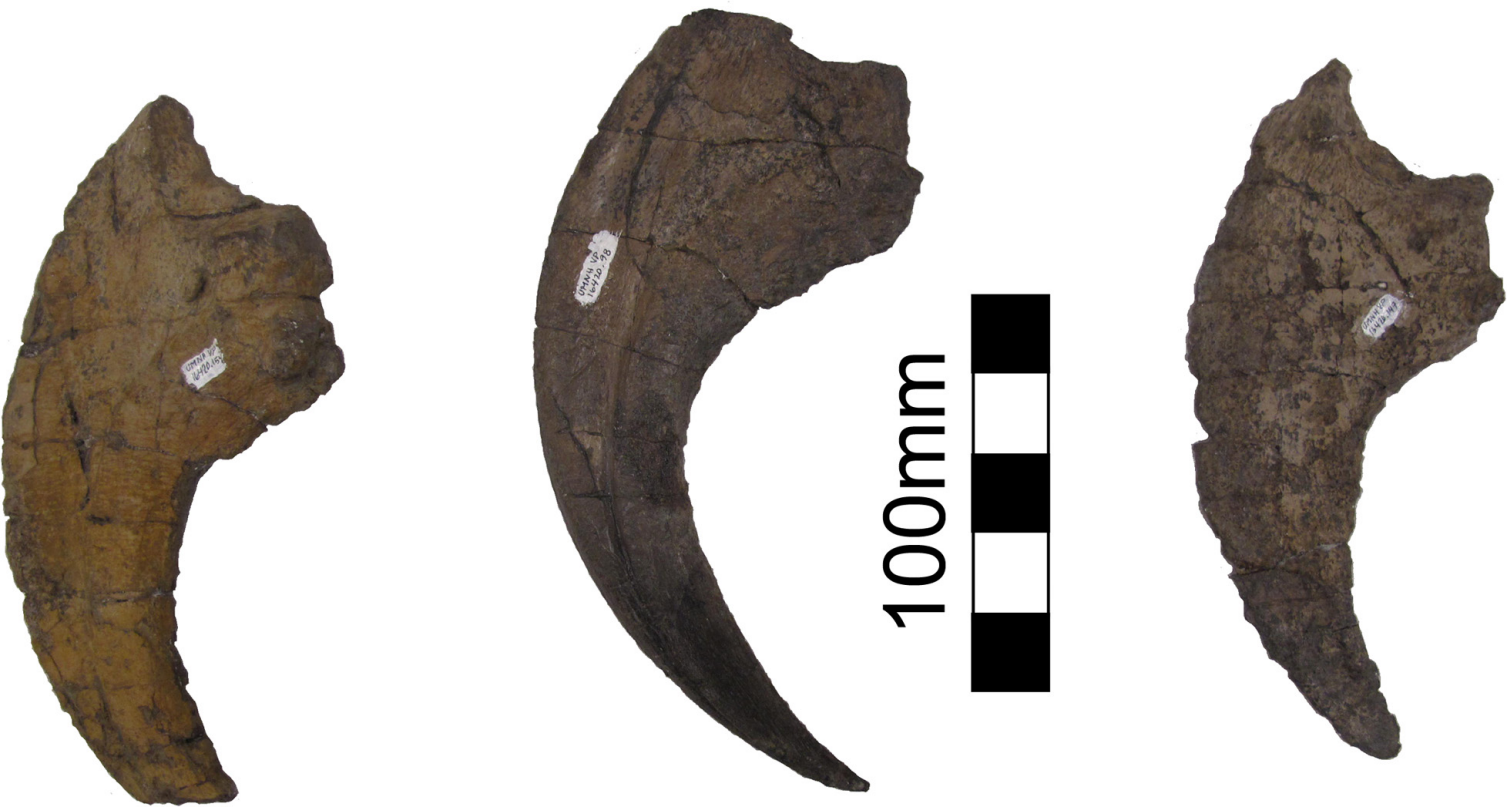
*Nothronychus* (UMNH VP16420) unguals. Three unguals medial to lateral from left to right in lateral view. Scale = 100 mm.

UMNH VP16420 (*No*. *graffami*) preserves metacarpals 1–3 (Fig 25), all three unguals (Fig 26), and a number of manual phalanges. MSM P2117 (*No*. *mckinleyi*) also preserves two manual phalanges, PI-1 and PII-1. There is no difference evident between the overlapping manual material in UMNH VP16420 and MSM P2117. Considering the lack of preservation in MSM P2117 and the excellent preservation of UMNH VP16420, the description of the manus will follow UMNH VP16420 unless otherwise noted. Metacarpals II and III are about half the length of the antebrachial elements. Metacarpal I is the most robust and shortest of the three, followed by metacarpal III, with metacarpal II being the longest, as in *Beipiaosaurus* and *Falcarius* [13, 17], and theropods generally. All the metacarpals have pronounced distal condyles, although they are all diagenetically distorted, especially metacarpal II. The proximal ends of the metacarpals are crushed and flattened, but there are two proximal condyles for articulation with the carpus on metacarpal II, though these condyles are much less pronounced than the distal condyles (Fig 25). The distal end of metacarpal II is asymmetric as in *Falcarius*, *Jianchangosaurus*, and *Erliansaurus* [15, 17, 30]. Metacarpal III is seemingly more slender than either metacarpal I or II, as in *Falcarius*, *Jianchangosaurus*, and *Beipiaosaurus* [13, 14, 30], though it is the most poorly preserved of the three.

There are six phalanges associated with UMNH VP16420 including PI-1, PI-2, PII-1, PII-2, PII-3, and PIII-4 with PI-2, PII-3, and PIII-4 representing unguals. The phalanges range in size from slightly larger than metacarpal I (90.5 mm) to longer than metacarpal II (141 mm). The phalanges on both digits I and II increase in length distally (PI-1 = 134 mm and PI-2 = 182 mm; PII-1 = 102 mm, PII-2 = 133 mm, PII-3 = 187 mm). The distal condyles are pronounced to the same degree as the metacarpals, similar to other therizinosaurians and theropods generally [13, 15, 17, 30]. The proximal surfaces have concave depressions for articulation with the preceding condyles, which are asymmetric on digit I, but symmetric on digit II. The phalanges on digits I and II are both elongate and gracile similar to *Erliansaurus*, *Beipiaosaurus*, and *Alxasaurus* [12, 13, 15]. They are unlike the shorter, more robust phalanges of *Falcarius* [17].

Of the two phalanges preserved in MSM P2117, the larger one is attributed to PI-1 and the slightly smaller one is attributed to PII-1. The two phalanges are generally similar, though the larger one is approximately 15 mm longer, more robust, and has an asymmetric proximal end. This asymmetry characterizes digit I rather than digit II allowing for the phalanges to be accurately referred. The smaller phalanx is referred to PII-1 based on its large size, generally symmetric condyles, and similarity to the PII-1 in *Erliansaurus*, *Beipiaosaurus*, and *Alxasaurus* where the phalanx is bifurcated distally into two robust subequal condyles [12, 13, 15].

Four ungual phalanges are preserved in UMNH VP16420, three of which are from the same hand (Fig 26). Both second unguals (PII-3) are preserved based on having similar size and morphology. The largest ungual is PI-2, followed by PII-3, and then PIII-4 similar to *Falcarius* and *Erliansaurus* [15, 17]. PI-2 and PIII-4 are broken, but PII-3 is complete. All three unguals have large articular surfaces for articulation with the phalanges as well as large tubercles for the insertion of the digital flexors (Fig 26). There is a deep groove extending along each ungual. PII-3 has a much more pronounced extensor pit than the other unguals, similar to *Falcarius* [17], but in contrast with *Jianchangosaurus* [30] and *Erliansaurus* [15].

#### Ilium

Both ilia of UMNH VP16420 (*No*. *graffami*) are firmly articulated to the sacrum. Neither ilium is preserved with MSM P2117 so this discussion only applies to *No*. *graffami*. The entirety of the sacrum and both ilia are dorsoventrally flattened such that the ilia flare laterally much more than would have been the case in life. It is difficult to know the orientation of the ilia due to the crushing. Other features such as the everted dorsal margin of the ilium as reconstructed by Zanno et al. ([2], Fig 1) may also be related to distortion and more material will be required in order to assess the concavity and convexity of parts of the ilia with certitude. *Segnosaurus* does have an everted dorsal margin on the ilium so it is possible that UMNH VP16420 preserves this as an undistorted structure.

The preacetabular processes splay outwards laterally and likely would have been ventrally oriented if uncrushed. The preacetabular processes are much longer than the postacetabular processes as in *Segnosaurus* [6], but not *Falcarius* or *Jianchangosaurus* [18, 30]. The preacetabular processes have strongly developed ventral projections (Fig 11) as in *Falcarius*, *Jianchangosaurus*, *Nanshiungosaurus*, and *Segnosaurus* [6, 7, 18, 30]. The ventral margin would have extended far ventral from the dorsal rim of the acetabulum in an uncrushed form as in other derived therizinosaurians. There is also a much less pronounced dorsal projection of the preacetabular process that is about 30% the size of the ventral projection on the dorsal aspect of the preacetabular process as in *Beipiaosaurus* [40] and derived therizinosaurians such as *Nanshiungosaurus* [7], *Suzhousaurus* [44] and *Segnosaurus* [6]. This is not present in basal forms such as *Falcarius* or *Jianchangosaurus*, which have flatter dorsal margins.

The acetabula have pronounced cranial rims that would have limited femoral protraction to some degree as in *Suzhousaurus* [44] and *Neimongosaurus* [14]. However, this could be preservational as previously noted. The acetabula are inset from the pre- and postacetabular processes and appear to be ventrally projecting rather than laterally projecting possibly due to the angulation of the femoral head. The supraacetabular crest is only partially visible in ventral orientation due to crushing of the acetabula upward into the sacrum, though it appears to be expanded as in other therizinosaurians (Fig 11) [14, 15, 18, 44]. The ilia form 50% of the acetabula with the ischia and pubes contributing far smaller proportions as in other therizinosaurians. The ischial peduncles are transversely broad and rugose, forming circular bosses (Fig 11). In contrast, the pubic peduncles are craniocaudally reduced and form a thin cup for the femoral head (Fig 11). These peduncle proportions are seen in all other derived therizinosaurians. The pubic and ischial peduncles of *Falcarius* and *Jianchangosaurus* have similar robusticity and overall size, which is not the case in more derived forms [6, 7, 15, 18, 30, 40, 44]. Zanno [18] first noted the evolution of a larger ischial peduncle relative to the pubic peduncle as a derived character present in therizinosaurians.

As in other derived therizinosaurians, the postacetabular processes of the ilia are reduced in comparison with the preacetabular processes. Due to diagenetic distortion, it is not possible to determine the relative angulation of these processes. The caudolateral portions of the postacetabular processes have a pronounced boss (Fig 11) as seen in *Segnosaurus* [6] and *Erliansaurus* [15] and to a lesser degree in *Neimongosaurus* [14] and *Suzhousaurus* [44]. This boss is not as pronounced in more basal forms [18, 30, 40]. The shape of the postacetabular process in therizinosaurians is characteristically triangular, but is squarer in UMNH VP16420 (Fig 11). The degree to which the postacetabular process is squared is difficult to assess considering the deformation, though it is clear that it was not simply acuminate as in less specialized therizinosaurian taxa. The presence of the enlarged boss on the caudolateral aspect could be related to the origins of the m. iliofibularis or m. flexor tibialis externus. Enhancement in the importance of these muscles may have caused the postacetabulum to become squarer in some derived therizinosaurians.

#### Pubis

**Fig 27:**
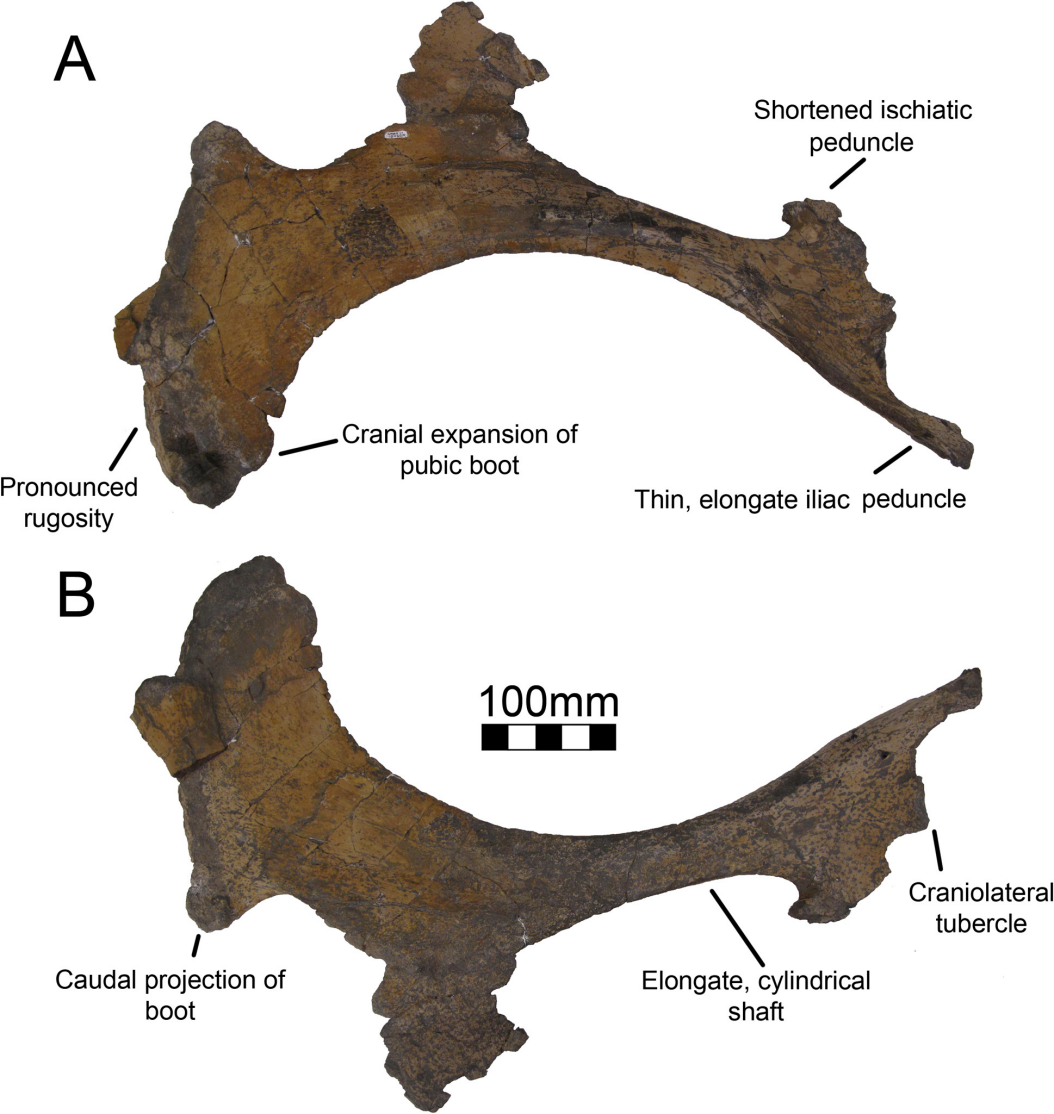
*Nothronychus* (UMNH VP16420) right pubis. Right pubis in (A) lateral and (B) medial views. Figure explanations on figure. Scale = 100 mm.

Both pubes of UMNH VP16420 (*No*. *graffami*) are virtually complete although the left pubis does not preserve the articulation with the ischium (Fig 27). Neither pubis is preserved in MSM P2117 (*No*. *mckinleyi*) and commentary on the pubes only applies to *No*. *graffami*. The pubis is fully retroverted and contacts the ischium along its long axis. The right pubis has only the proximal part of the obturator process of the ischium fused to it, a character distinguishing the genus *Nothronychus* from other therizinosaurians. This feature can be confirmed for MSM P2117 based on the ischia. The contact between the pubis and ischium in UMNH VP16420 is thickened. The left pubis does not preserve much of the proximal articulation; otherwise, the pubes are identical. The iliac peduncle of the pubis is not thickened along the acetabular margin and comes to a sharp edge. The contribution of the pubes to the acetabula is extensive on the cranial and ventral aspects as in *Suzhousaurus* and *Segnosaurus* [6, 44]. There is a pronounced craniolateral tubercle proximally as in *Falcarius*, which is not seen in *Suzhousaurus* [18, 44]. The ischiatic peduncle of the pubis is much smaller than the iliac peduncle and is located closer proximodistally to the shaft of the pubis (Fig 27). Neither peduncle is as expanded or robust as in *Suzhousaurus* [44] and more resembles the condition in *Segnosaurus* [6]. The ischiatic peduncle has a shallow sulcus on the ventral aspect forming the dorsal border of the obturator foramen as in other therizinosaurians.

The shaft of the pubis is elongate and cylindrical unlike the mediolaterally-flattened form of *Suzhousaurus* [44]. Further, it has a slight medial deflection in contrast with the lateral deflection of FRDC-GSJB-2004-001 [Fossil Research and Development Center of the Third Geology and Mineral Resources Exploration Academy of Gansu Province, Lanzhou, China] (*Suzhousaurus*; [44]), though compression could play a role in the apparent deflection of either of these specimens. The pubes of *Falcarius* are only slightly sigmoid lacking the cranial concavity present in UMNH VP16420 resulting from the enlarged boot [18]. However, the oval cross section of the shaft in *Falcarius* is similar to that of UMNH VP16420.

Distally, the pubes bear a cranially expanded pubic boot as in other therizinosaurians (Fig 27) [6], though it is much more pronounced than in less specialized forms such as *Falcarius* and *Beipiaosaurus* [18]. The boot is rugose and is also somewhat transversely expanded, especially on the lateral aspect (Fig 27). The caudal aspect of the pubic boot has a slight caudolateral projection as in *Falcarius* [18], but is relatively diminutive in comparison with other taxa. The weak caudal projection on the pubic boot distinguishes *No*. *graffami* from other therizinosaurian taxa. Just proximal to the boot is the attachment with the ischium. There is a sulcus between the boot and the obturator process. The medial aspect of the pubic boot is slightly rugose, but the medial aspect of the pubis in general is without rugosity. The connection for the m. ambiens is not defined.

#### Ischium

**Fig 28:**
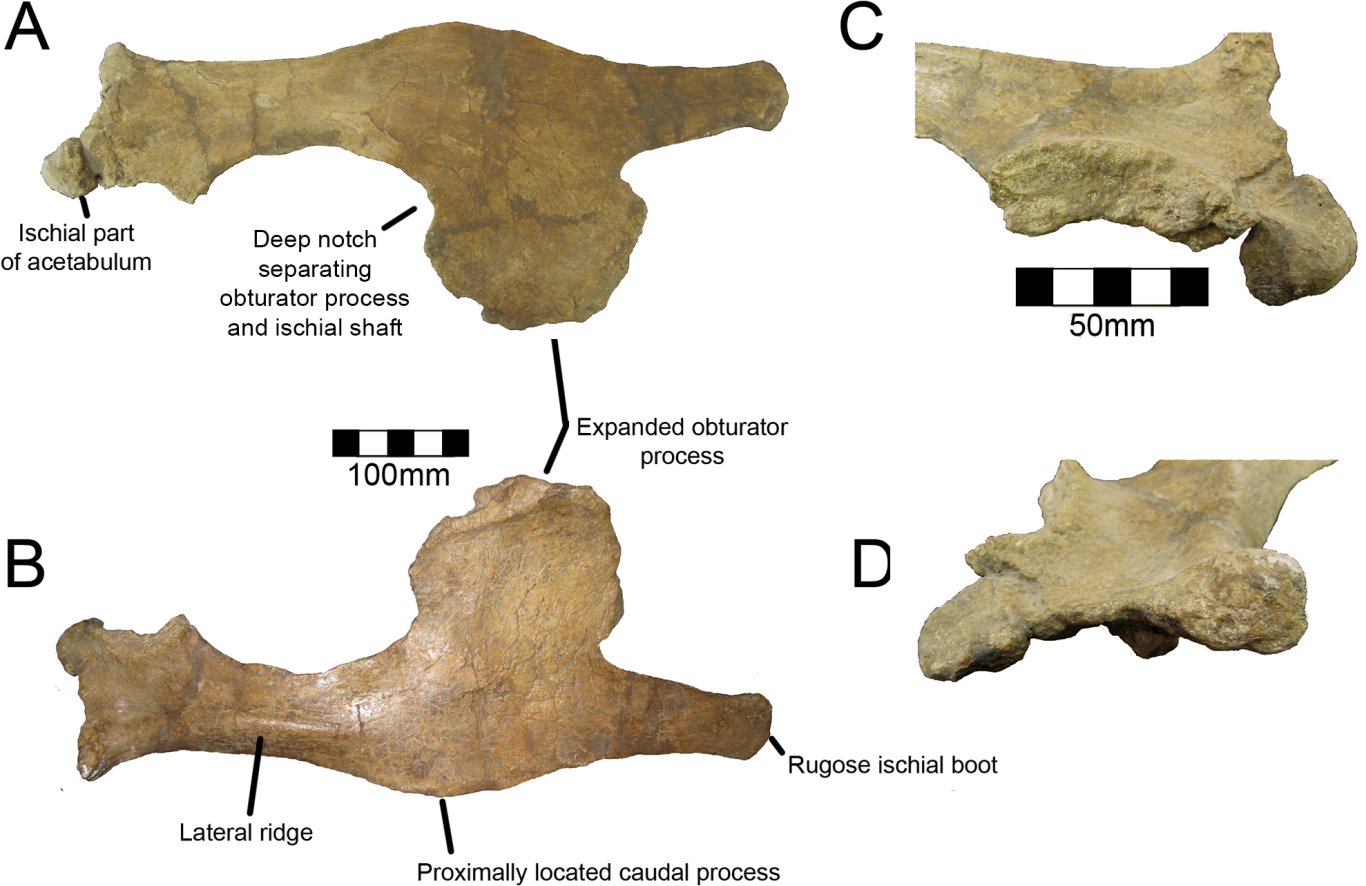
*Nothronychus* (MSM P2117) right ischium. Right ischium in (A) lateral and (B) medial views. Figure explanations on figure. Scale = 100 mm. The (C) pubic and (D) iliac articulations shown closer. Scale = 50 mm.

**Fig 29:**
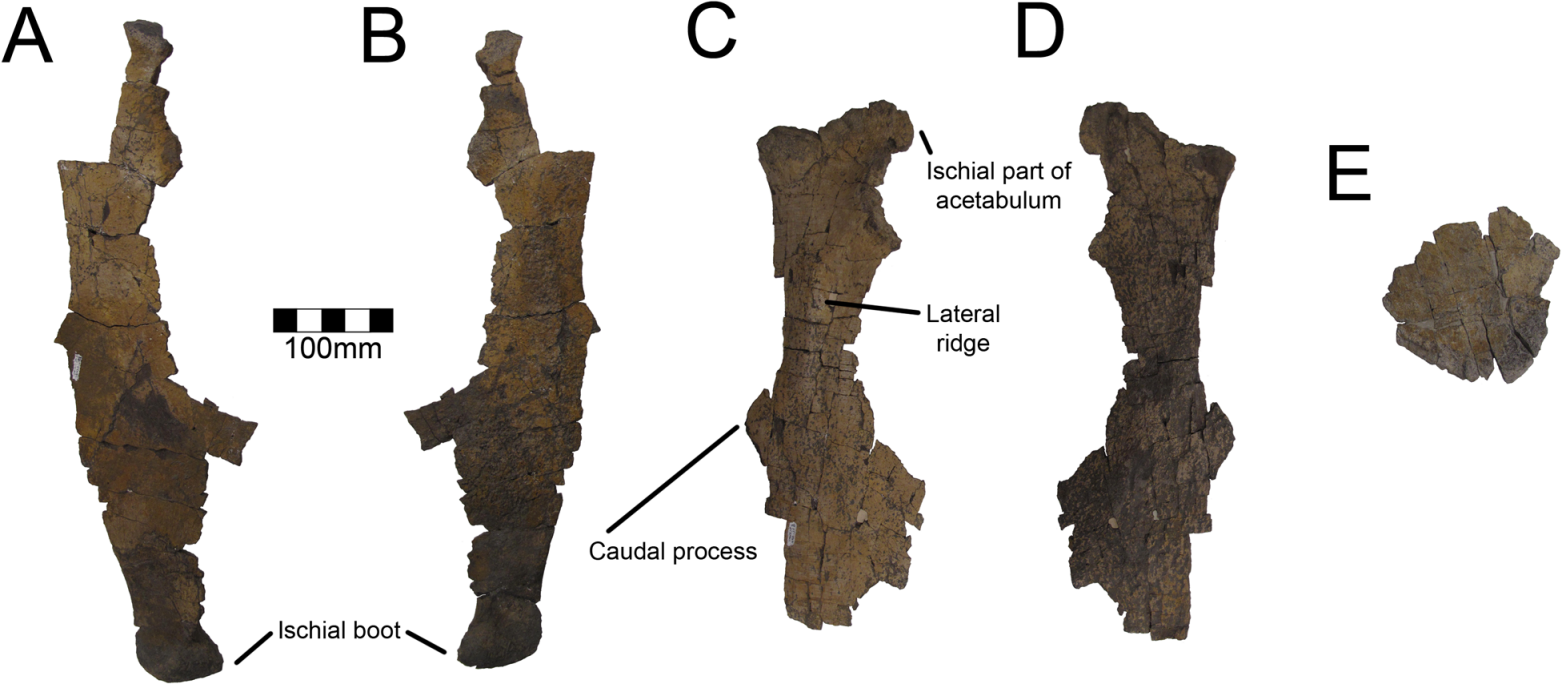
*Nothronychus* (UMNH VP16420) ischia. Left ischium in (A) medial and (B) lateral view. Right ischium in (C) lateral and (D) medial views. (E) Broken obturator process. Scale = 100 mm.

Both ischia are preserved for MSM P2117 (*No*. *mckinleyi*) (Fig 28) and UMNH VP16420 (*No*. *graffami*) (Fig 29), but they are partial for UMNH VP16420. Therefore, the description primarily follows MSM P2117. The ischia of both specimens are characteristic for derived therizinosaurians by virtue of the expanded obturator process. The subcircular obturator process found in both species of *Nothronychus* differentiates *Nothronychus* from other therizinosaurian genera. The ischia are subequal in size to the pubes not considering the size of the pubic boot. This character is also seen to a lesser degree in less specialized therizinosaurian forms [18, 30]. The obturator process is broken in *Falcarius*, but shows slight expansion suggesting hypertrophy of the process only developed in more derived forms [18]. This is further supported by the modest development of the obturator process in *Jianchangosaurus* [30]. In contrast with *Falcarius*, the shafts of the ischia of MSM P2117 are straight as in *Jianchangosaurus*, *Suzhousaurus*, and *Segnosaurus*. However, both *Nothronychus* species have a deep notch separating the ischial shaft and obturator process unlike other therizinosaurians. This is narrower in MSM P2117 than in UMNH VP16420 and serves as a feature distinguishing the two species. As in other therizinosaurians, the iliac and pubic peduncles are flattened medially and expanded laterally. The iliac and pubic peduncles are approximately equal in length (length iliac peduncle: 73.5 mm; length pubic peduncle: 74.3 mm). The iliac peduncle is twice the length of the pubic peduncle in *Falcarius* [18]. They are subequal in *Segnosaurus* [6] and *Suzhousaurus* [44], which may reflect an evolutionary trend toward the development of a more robust ischium in therizinosaurians. There is a dorsally projecting prominence between the peduncles, which forms the ischiadic portion of the acetabulum. The prominence is substantial in MSM P2117, but shows that the ischium does not contribute a significant portion of the acetabulum similar to other therizinosaurids and *Suzhousaurus*.

The caudal process of the ischium of MSM P2117 is pronounced and extends approximately one-third the length of the ischium (Fig 28). The caudal process of the ischium is considered small in UMNH VP16420 and is positioned above the dorsal aspect of the obturator process (Fig 29) [2]. However, the caudal aspects of the ischia in UMNH VP16420 are damaged, so it is possible that the caudal processes extend further ventrally. Further finds are required to definitively determine if this character can reliably be used to separate the species. Until such time it is retained as a distinguishing feature. The caudal process of *Suzhousaurus* is located distal to the top of the obturator process and is much more pronounced than in MSM P2117 [44]. It is much smaller in *Segnosaurus* and exactly opposite the obturator process [6]. This feature is seemingly absent in *Falcarius* and *Jianchangosaurus* [18, 30]. Along the lateral shaft of the ischium of MSM P2117 and UMNH VP16420 proximal to the obturator process is a prominent ridge extending from the iliac peduncle distally. This ridge likely corresponds to one of the heads of the adductor muscle [45]. An incipient ridge is present in *Falcarius* [18]. *Segnosaurus* has a rather pronounced expansion in the middle of the shaft, though it was originally considered “insignificant” [6]. There is a distal boot ventral to the obturator process extending one-fourth the length of the ischium in both species. The projection is thin transversely and ends in a rugose ischial boot. The boot does not expand craniocaudally and is slightly deflected laterally, unlike *Suzhousaurus* where it is much more robust, although this could be size related since *Suzhousaurus* is a larger animal [44]. It is also not especially cranially expanded unlike the condition in *Jianchangosaurus* where the distal boot comes to a cranioventrally oriented point [30].

#### Femur

**Fig 30:**
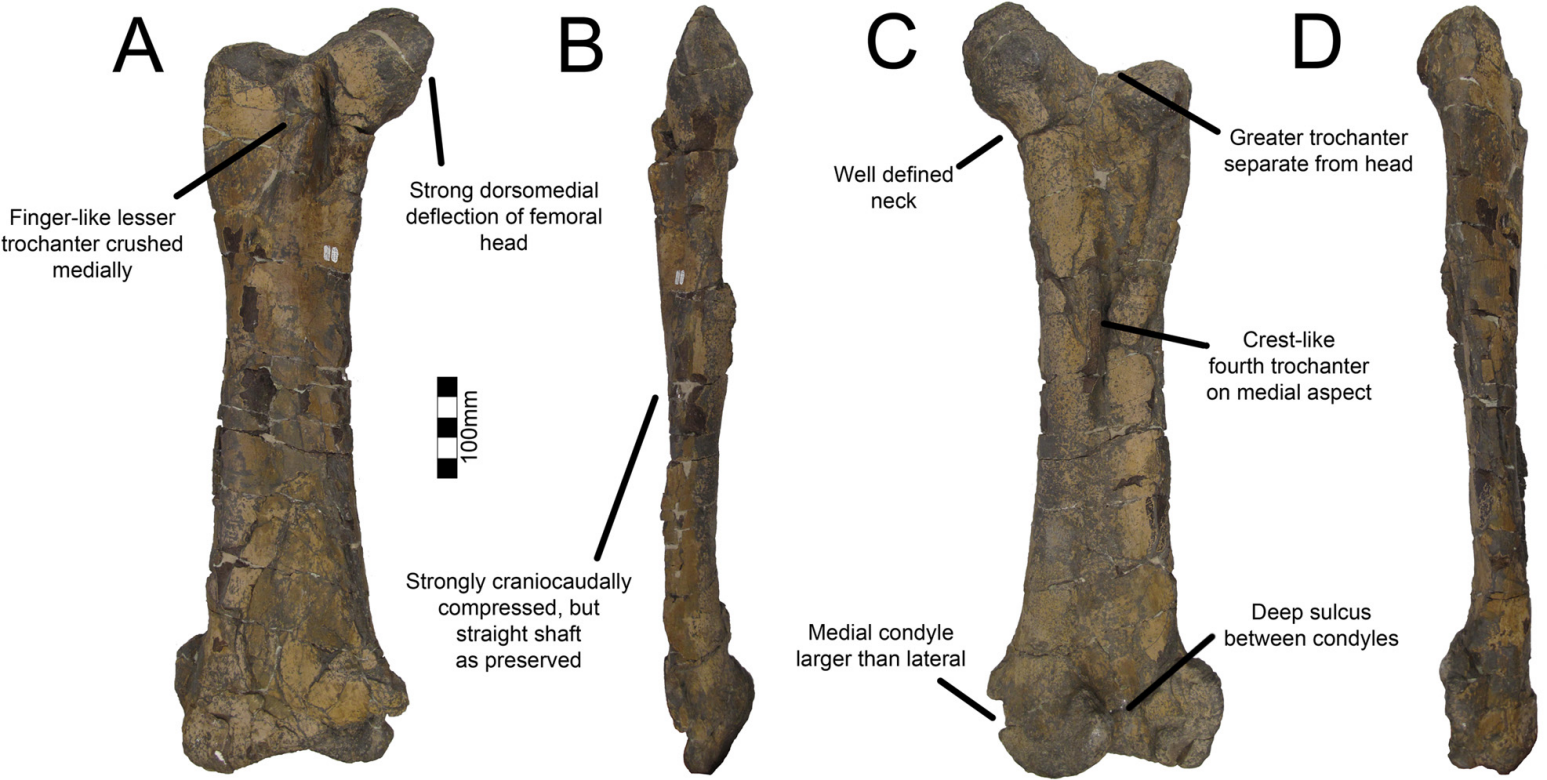
*Nothronychus* (UMNH VP16420) right femur. Right femur in (A) cranial, (B) medial, (C) caudal, and (D) lateral views. Figure explanations on figure. Scale = 100 mm.

Both femora of UMNH VP16420 (*No*. *graffami*) are preserved (Fig 30) and neither femur is known for MSM P2117 (*No*. *mckinleyi*) so all features described only apply to *No*. *graffami*. The femora of UMNH VP16420 are not sigmoid as they are in *Falcarius* [18] or bowed as in *Alxasaurus* and *Suzhousaurus* [12, 44]; however, this may be due to diagenetic distortion. Both femora are strongly craniocaudally compressed and are robust. The femoral cross section is wider transervsely than long craniocaudally, likely a preservational artifact. The head of the femur is rugose and the width of the head is nearly the same width as the midshaft of the femur. The head is strongly dorsally deflected on both sides (Fig 30), similar to *Alxasaurus* and *Suzhousaurus* [12, 44] and unlike the perpendicular orientation in *Falcarius*, *Jianchangosaurus*, *Beipiaosaurus*, and *Neimongosaurus* [13, 14, 18, 30].

The greater trochanter of the right femur is better preserved than the left. The greater trochanter is completely separated from the head (Fig 30) as in other derived therizinosaurians (*Suzhousaurus*, *Neimongosaurus*, *Erliansaurus*, *Segnosaurus*), but unlike basal forms (*Falcarius*, *Beipiaosaurus*) [18]. The left greater trochanter has been medially inset due to crushing such that the entire trochanter is sheared (Fig 30). The lesser trochanter is somewhat medially placed rather than restricted to the craniolateral aspect of the femur. This may also be related to diagenetic deformation. The dorsal edge of the trochanter is rugose and the ventral ridge is smooth. There is a deep sulcus on the medial aspect of the lesser trochanter similar to *Falcarius*, *Jianchangosaurus*, *Alxasaurus* and *Segnosaurus* [6, 12, 18, 30]. The lesser trochanter is closely oppressed to the greater trochanter. There is no accessory trochanter on the lesser trochanter, which is present in *Falcarius* [18].

The fourth trochanter is located just proximal to midshaft as in other theropod taxa and extends distally to the midshaft as in *Suzhousaurus* (Fig 30) [44]. It is more robust than in most theropods forming a long, narrow ridge similar to *Suzhousaurus*. The lateral aspect of the fourth trochanter gently rises to the apex, whereas the medial aspect is slightly concave with the apex of the trochanter having a medial deflection (Fig 30). The entire fourth trochanter is located on the medial aspect of the bone. There is a deep sulcus along the caudal aspect of the femur just lateral to the fourth trochanter and it extends for most of the length of the bone (Fig 30). This is likely more pronounced than in life due to postmortem distortion. The medial distal condyle is more robust than the lateral condyle as in *Falcarius*, *Suzhousaurus*, and *Neimongosaurus* [14, 18, 44], but unlike *Erliansaurus* in which they are subequal in size and robusticity [15]. The lateral condyle of UMNH VP16420 is craniocaudally distorted in both femora. The flexor intertrochanteric groove is wide and deep such that the craniocaudal distance between the condyles is the thinnest aspect of the bone. The groove is also especially developed in *Neimongosaurus* and *Segnosaurus* [6, 14]. There is a slight groove between the condyles on the extensor side as well. The distal condyles on both sides are laterally deflected due to crushing.

#### Tibia

**Fig 31:**
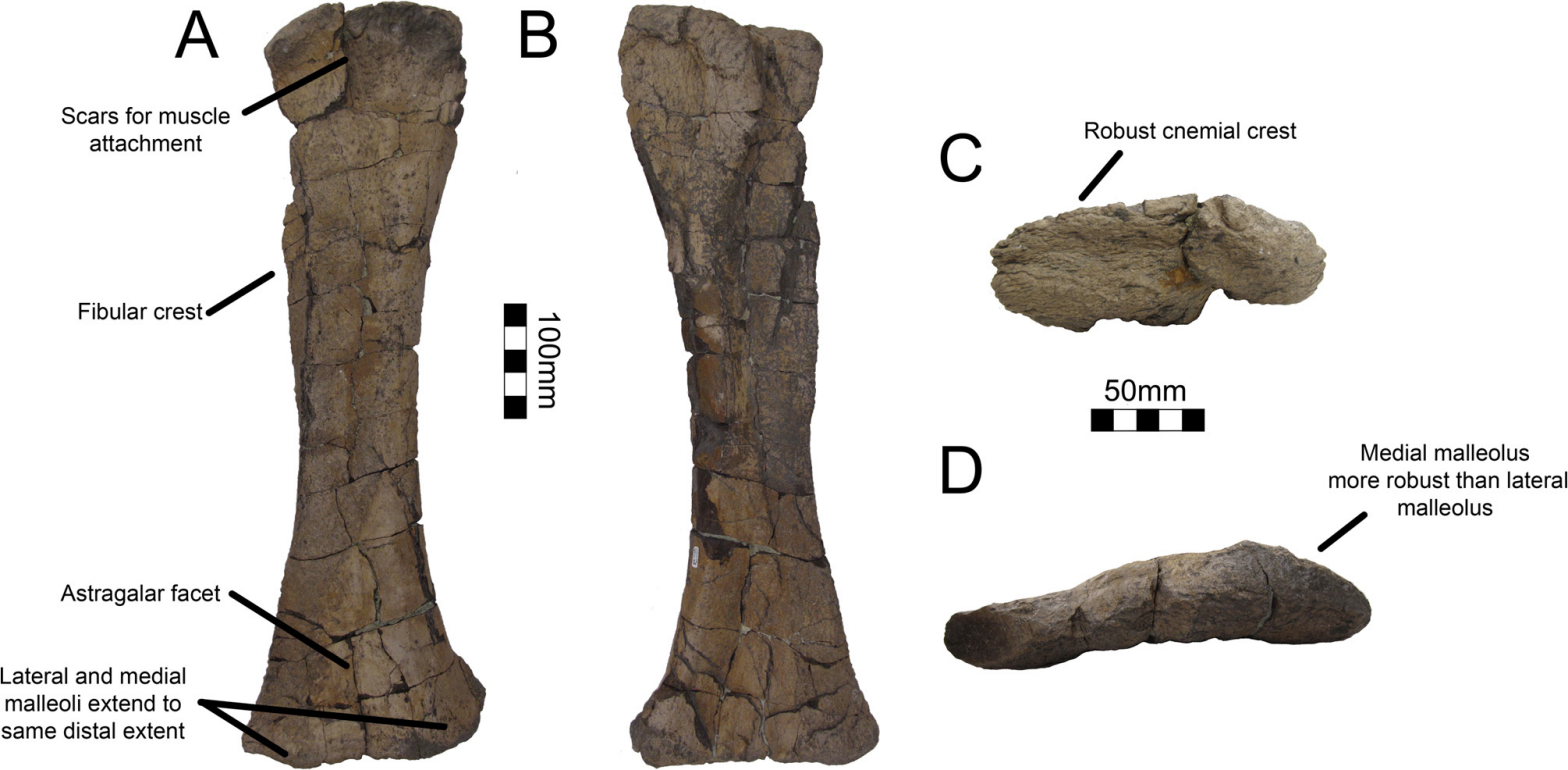
*Nothronychus* (UMNH VP16420) right tibia. Right tibia in (A) cranial, (B) caudal, (C) proximal, and (D) distal views. Figure explanations on figure. Scale = 100 mm for A and B. Scale = 50 mm for C and D.

**Fig 32:**
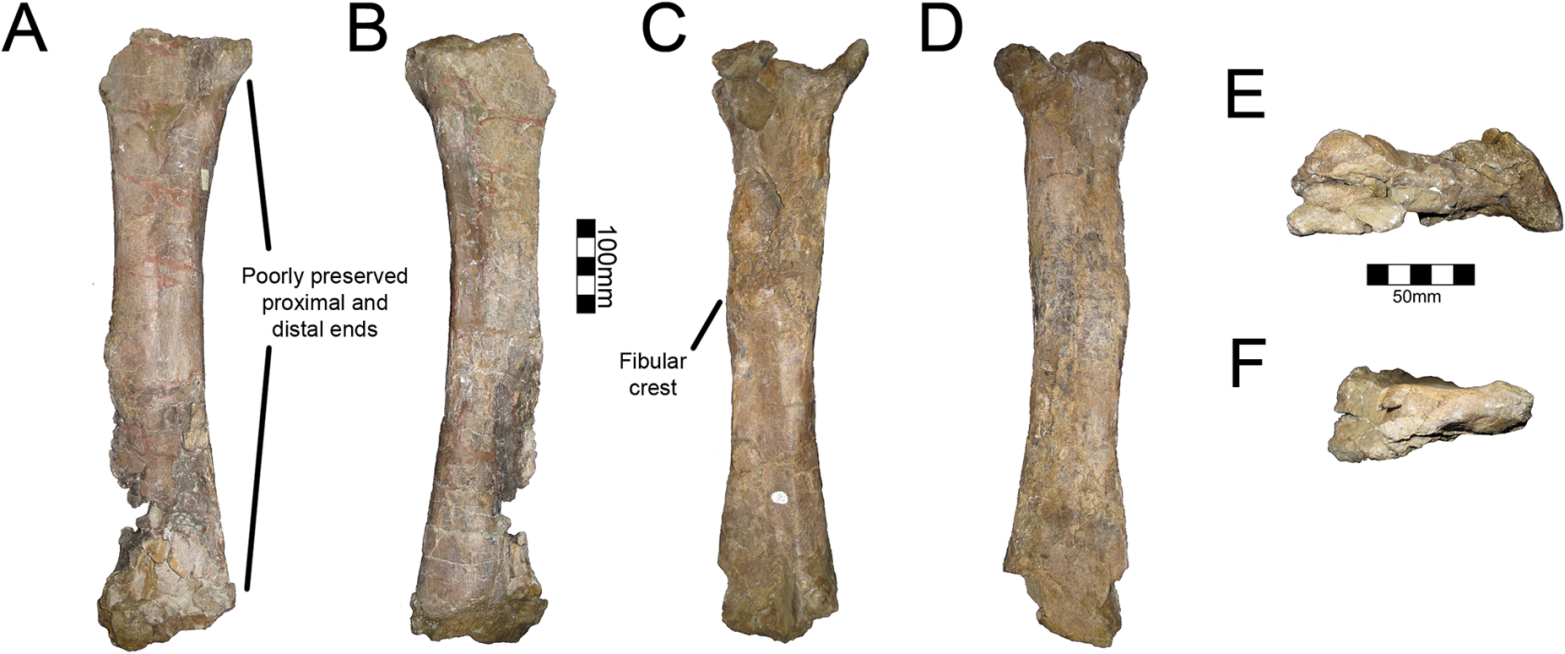
*Nothronychus* (MSM P2117) tibiae. Right tibia in (A) cranial and (B) caudal views. Left tibia in (C) caudal and (D) cranial views. Scale = 100 mm. (E) Proximal left tibia. (F) Distal left tibia. Scale = 50 mm.

Right and left tibiae are preserved in UMNH VP16420 (*No*. *graffami*) and MSM P2117 (*No*. *mckinleyi*) (Figs 31 and 32); however, the left tibia of UMNH VP16420 is the only complete example with both proximal and distal ends (Fig 31). The left tibia of UMNH VP16420 is craniocaudally compressed, yet all of its features are evident. The cnemial crest is robust and strongly laterally deflected due to crushing (Fig 31). The cnemial crest of *Falcarius* is incipient in contrast. Craniocaudal crushing precludes the confirmation that the proximal tibia forms a triangle and does not allow assessment of which structure (the cnemial crest, as in *Falcarius*, or the lateral condyle, as in more specialized therizinosaurians [18]), forms the apex. The cranial aspect of the proximal tibia has a row of three depressions for the insertion of the proximal leg musculature (Fig 31). The medial depression is the largest. The lateral aspect of the tibia also has a pronounced tubercle for muscle insertions. The intercondylar groove between the proximal condyles is not very pronounced, but this is probably because the lateral and medial condyles are medially compressed. As in *Falcarius*, the medial condyle is larger and more robust than the lateral condyle [18]. Neither tibia of MSM P2117 nor the right tibia of UMNH VP16420 preserves the proximal end so these features only apply to *No*. *graffami*.

The fibular crest of the tibia is extensive and continues distally to the midshaft as in *Erliansaurus* and *Neimongosaurus* and unlike *Falcarius* in both *Nothronychus* species. (Fig 31) [6, 14, 15, 18]. The lateral malleolus and medial malleolus are equally extensive distally, unlike *Neimongosaurus* in which the lateral malleolus is more distally extensive [14]. Both distal malleoli approximate one another in overall area, though the medial malleolus is slightly more robust. There is a pronounced groove along the caudal surface running from the proximal intercondylar groove to the distal end, flattening out at about the level where the distal malleoli start to splay outwards. There is a small facet for the ascending process of the astragalus on the cranial surface as in other therizinosaurians (Fig 31). The right tibia and the tibiae of MSM P2117 exhibit all of these features demonstrating that they are not the result of diagenetic alteration. The tibiae have no known features distinguishing the two *Nothronychus* species.

#### Fibula

**Fig 33:**
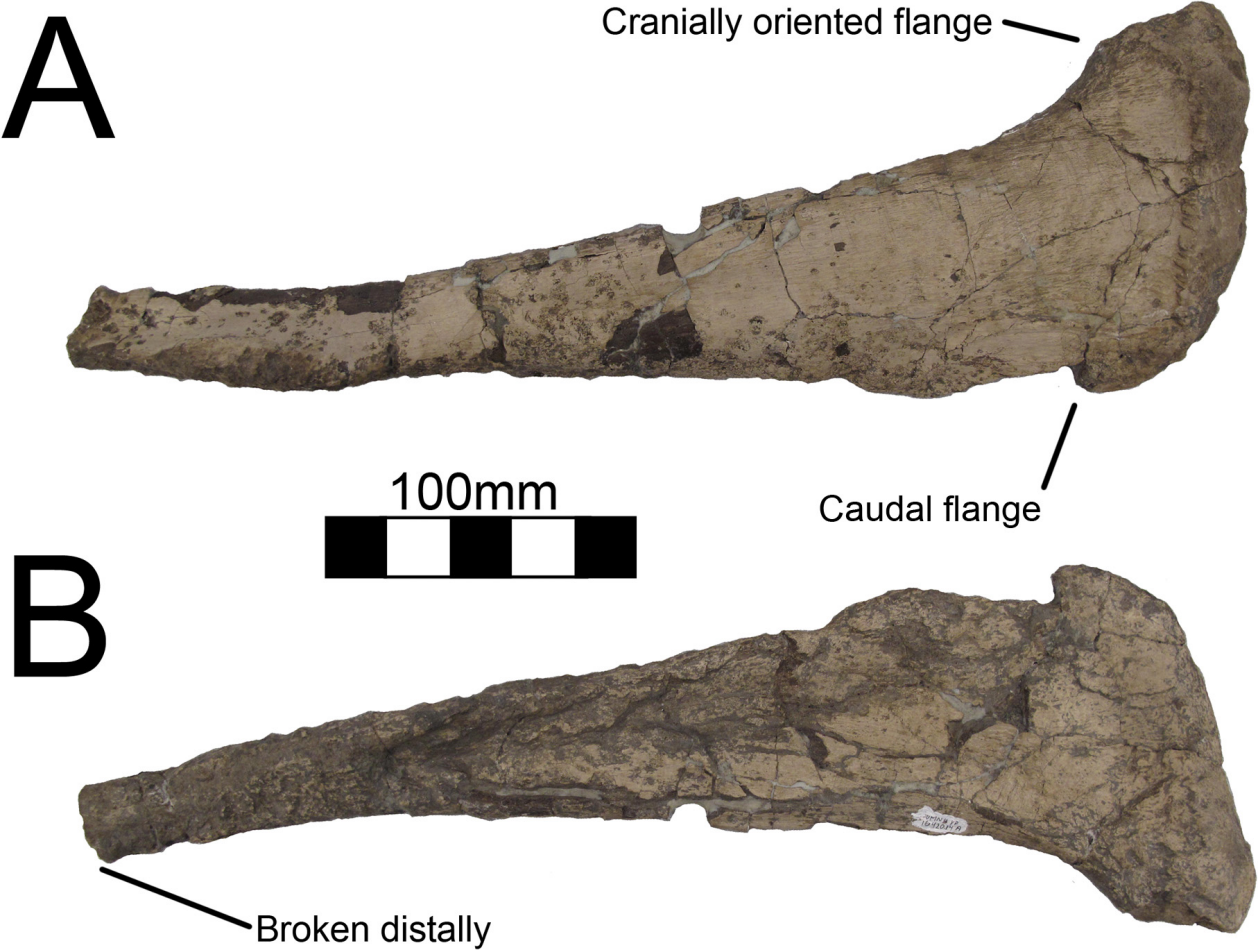
*Nothronychus* (UMNH VP16420) left fibula. Left fibula in (A) lateral and (B) medial views. Scale = 100 mm.

**Fig 34:**
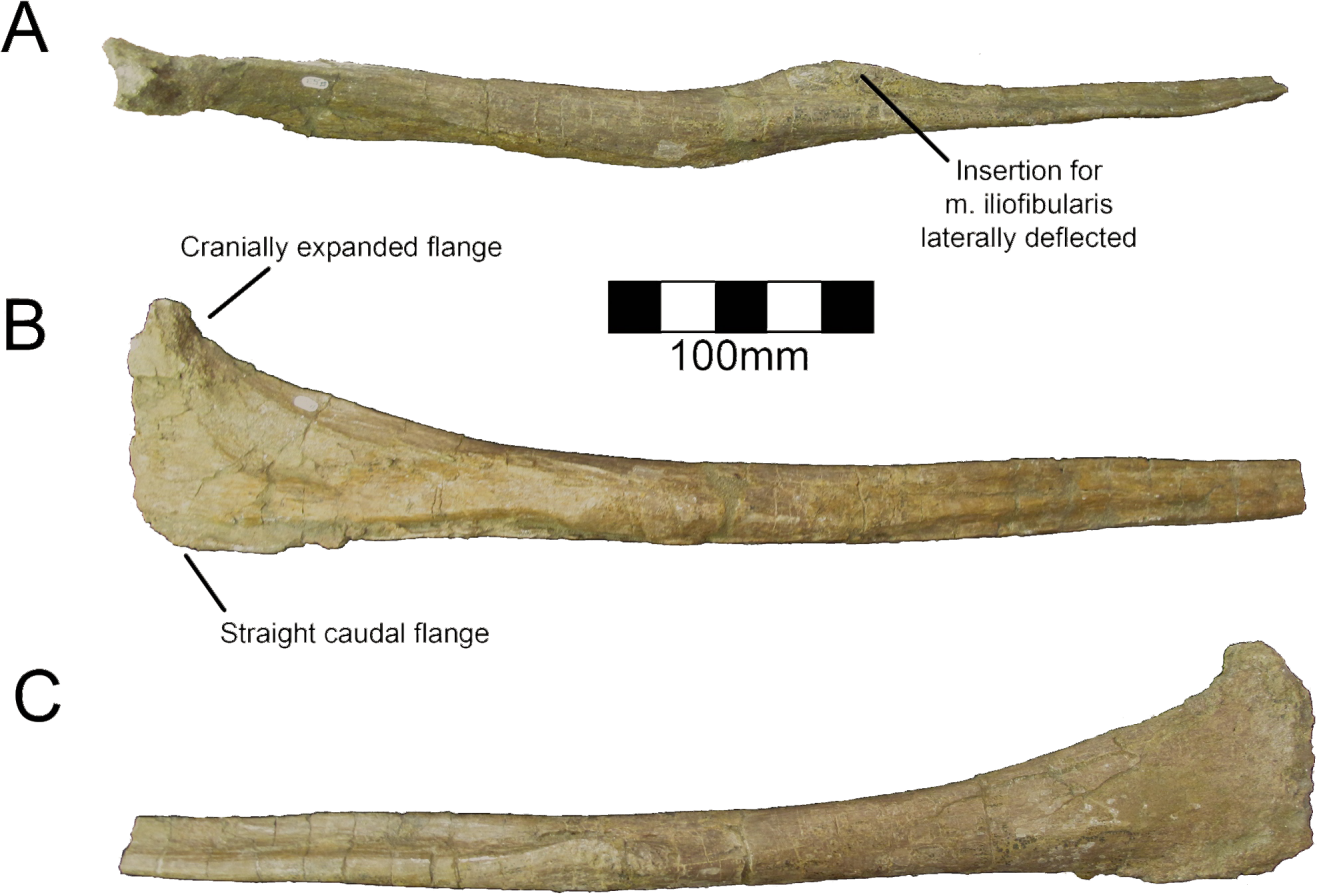
*Nothronychus* (MSM P2117) left fibula. Left fibula in (A) cranial, (B) medial and (C) lateral views. Figure explanations on figure. Scale = 100 mm.

UMNH VP16420 (*No*. *graffami*) preserves both fibulae (Fig 33) and MSM P2117 (*No*. *mckinleyi*) preserves the right fibula (Fig 34), which is the best preserved and most complete of the three. The discussion of the fibula will refer to MSM P2117 except when stated otherwise as there are no differences apparent between the fibulae of the two species. The proximal part of the right fibula of MSM P2117 and the majority of the shaft are preserved, though it is broken distally as it is in UMNH VP16420. The proximal aspect has a hypertrophied cranially directed flange for articulation with the tibia and then thins substantially distally (Fig 34). The caudal aspect of the flange is not proximally deflected as in *Erliansaurus* [15] (Fig 34). The shaft is straight and is entirely flat for articulation with the fibular crest of the tibia as in UMNH VP16420 and *Erliansaurus* [15]. The tubercle for insertion of the m. iliofibularis is hypertrophied and is located 295 mm from the proximal end of the bone (Fig 34). The tubercle is not preserved on UMNH VP16420 as the bone is broken proximal to where the tubercle would have been located. The tubercle for the m. iliofibularis is preserved slightly proximal to midshaft as in *Jianchangosaurus*, *Erliansaurus* and UMNH VP16420 [2, 15, 30] presuming that the fibula extended to the distal end of the tibia. Distal to this tubercle, the fibula becomes much more reduced as in other therizinosaurians [6, 15]. Based on the tibiae, the fibulae were approximately 600–610 mm in total length, if they extended distally to the end of the tibiae as in other therizinosaurids.

#### Astragalus

**Fig 35:**
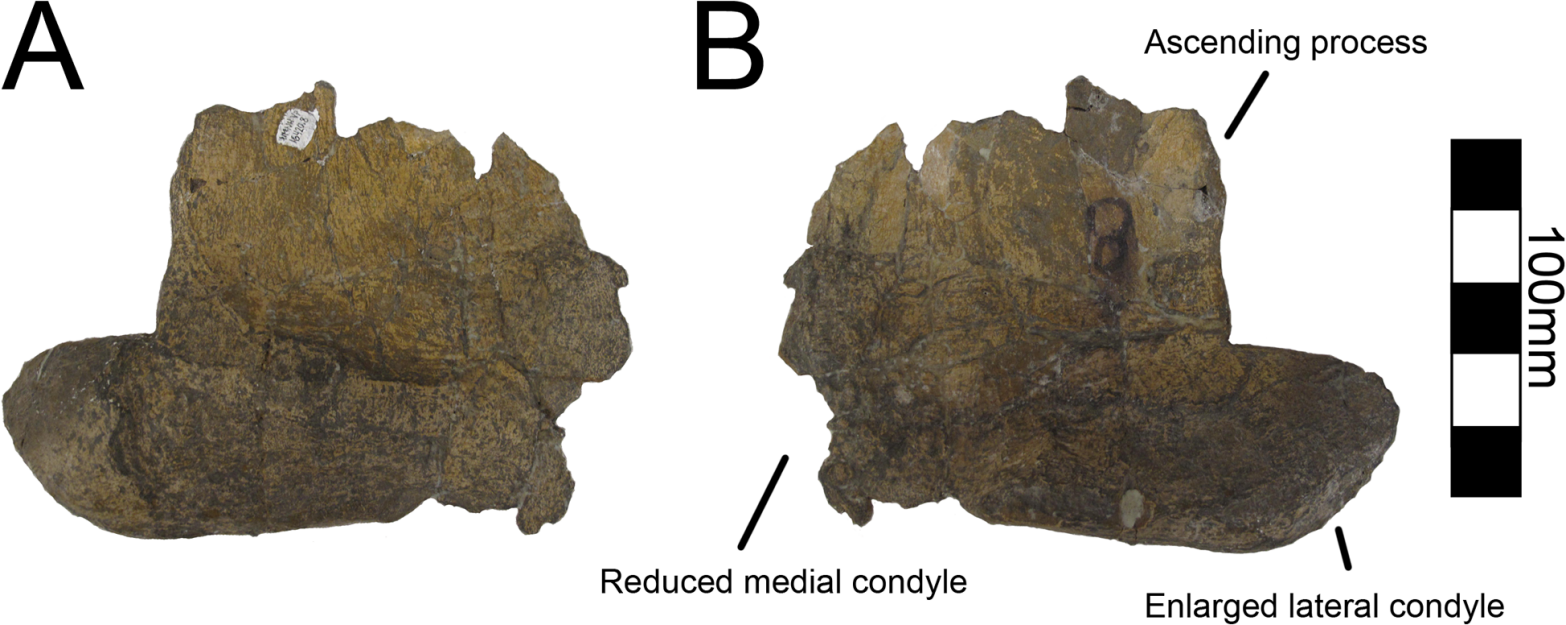
*Nothronychus* (UMNH VP16420) right astragalus. Right astragalus in (A) cranial and (B) caudal views. Figure explanations on figure. Scale = 100 mm.

Both astragali of UMNH VP16420 (*No*. *graffami*) are preserved disarticulated from the tibiae (Fig 35). The lateral process of the astragalus is much more pronounced and robust than the medial process. The medial process is a thin layer of bone and is better preserved on the right astragalus. The lateral process is thickened into a bulb (Fig 35). The thickened aspect extends to the midline of the bone medially and caudally to cup the distal end of the tibia. This leaves the lateral surface of the tibia exposed in cranial aspect as in other specialized therizinosaurids such as *Segnosaurus* and *Therizinosaurus*. Zanno et al. [2] noted the reduction of the astragalar body as a synapomorphy of therizinosaurids. The ascending process is situated along the medial side on the right astragalus and is broken on the left. It is not clear if there is a lateral projection of the ascending process proximally as in *Segnosaurus* [6] and *Therizinosaurus* [5] due to breaks on the proximal end. However, Zanno et al. [2] consider the morphology to suggest a lateral projection. The ascending process as preserved extends about 200% the height of the astragalar body and is a thin sheet of bone proportionally similar to *Falcarius* [18] and *Segnosaurus* [6].

#### Metatarsals

**Fig 36:**
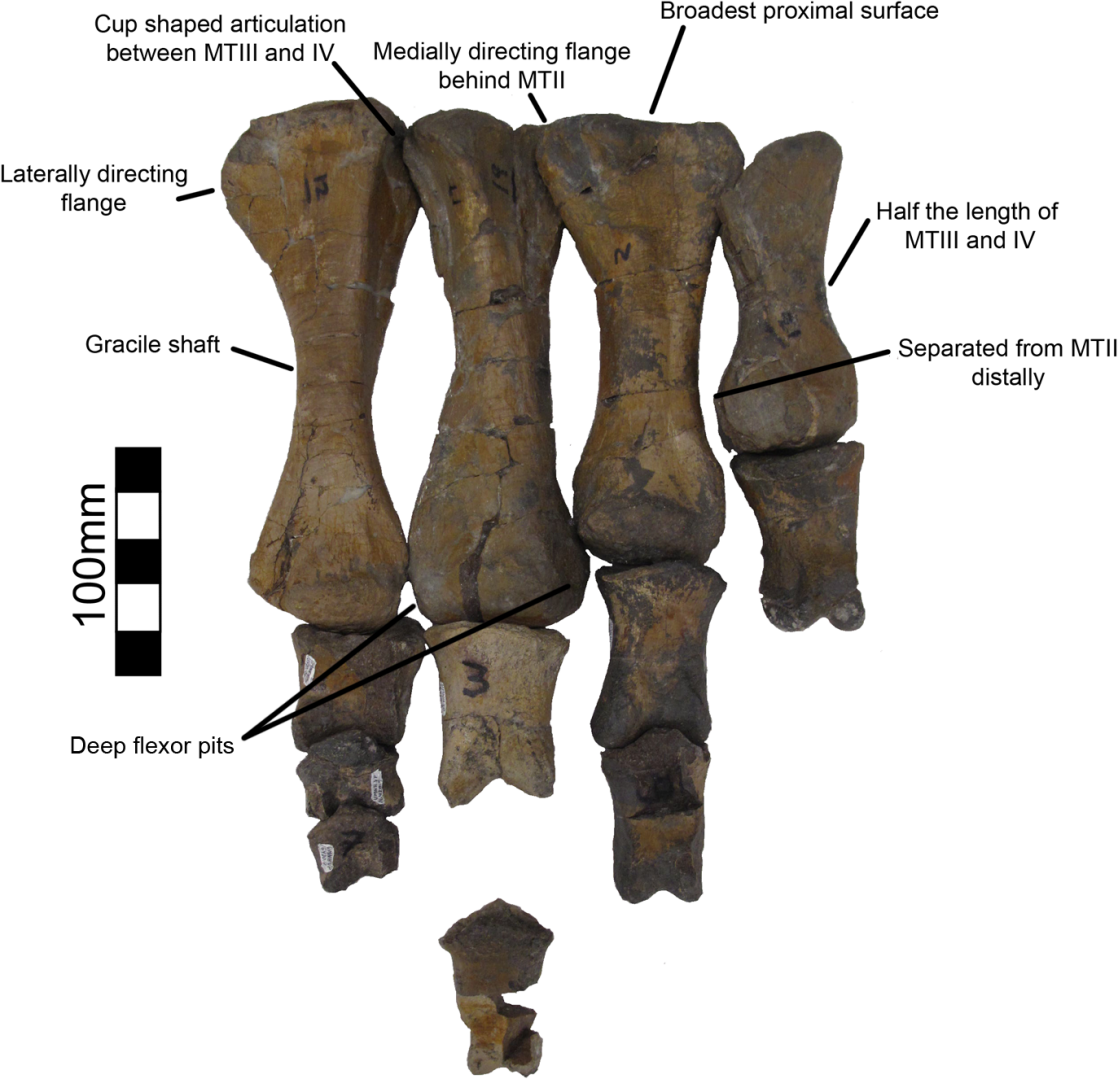
*Nothronychus* (UMNH VP16420) right pes. Right pes including metatarsals I-IV and associated phalanges articulated as in life. Figure explanations on figure. Scale = 100 mm.

All four metatarsals of the left foot and a number of pedal phalanges are known for UMNH VP16420 (*No*. *graffami*) (Fig 36). A right metatarsal II and IV are also preserved. One partial metatarsal is known with MSM P2117 (*No*. *mckinleyi*). There are no differences evident between the two species based on the preserved material, but the majority of the description only pertains to *N*. *graffami* due to a lack of metatarsal materials for *N*. *mckinleyi*.

Metatarsal I is the smallest of the metatarsals and is approximately half the length of metatarsals III and IV (Fig 36) as in *Neimongosaurus*, *Segnosaurus*, and *Therizinosaurus* [5, 6, 14], yet dramatically different from *Falcarius*, which has a diminutive and splinted metatarsal I [18]. The trend for a functional tetradactyl foot in therizinosaurians is pronounced in the highly specialized forms *Segnosaurus* and *Therizinosaurus*, although shortening and increasing robusticity of the metatarsus is already observed in less specialized forms such as *Falcarius* [18]. The distal end retains a single condyle for articulation with the proximal phalanx. The proximomedial aspect extends much further proximally than the lateral side, unlike the other metatarsals (Fig 36) yet similar to *Segnosaurus* and *Therizinosaurus* [5, 6]. The lateral side fits snugly with the medial side of metatarsal II, but is deflected as in *Neimongosaurus*, *Segnosaurus*, and *Therizinosaurus* [5, 6, 14].

Metatarsal II is longer than metatarsal I, but is shorter than either metatarsal III or IV. This is also the case in *Segnosaurus* [6]. In *Falcarius*, metatarsal II and III are subequal in length [18]. The proximolateral condyle has a slight cranial deflection so the medial flange on metatarsal III can fit tightly on the caudal surface. Unlike metatarsal III and IV, there is no flattened proximal flange. Both sides of the proximal end are craniocaudally thickened giving metatarsal II the broadest proximal proportions of the metatarsals (Fig 36) as in *Neimongosaurus* and possibly *Falcarius*, but unlike *Segnosaurus* [6, 14, 18]. The distal end is also composed of a single condyle, unlike the bicondylar condition in *Falcarius* [18]. There are pronounced collateral ligament pits on either side of the condyle. The metatarsal associated with MSM P2117 is likely the distal end of metatarsal II. Both specimens UMNH VP16420 and MSM P2117 are generally the same size, and the metatarsal associated with MSM P2117 is the same size and shape as metatarsal II of UMNH VP16420.

Metatarsal III is subequal in size with metatarsal IV as in *Segnosaurus* [6]. The proximal end has a medially directed flange to fit caudal to metatarsal II (Fig 36). The lateral aspect is thickened to form a rounded surface for articulation with metatarsal IV (Fig 36), as in *Falcarius* and *Segnosaurus* [6, 18]. The distal end has two pronounced condyles (Fig 36) as in *Segnosaurus* [6] rather than a single pronounced condyle as in metatarsal III or IV of *Falcarius* [18]. Further, unlike metatarsal IV, there are deep flexor and extensor pits on the sides of the condyles as in metatarsal III of *Falcarius* [18].

Metatarsal IV is the most robust and longest of the metatarsals. Metatarsal IV is somewhat smaller than metatarsal III in both *Falcarius* and *Segnosaurus* [6, 18]. The proximal end is triradiate as in *Falcarius* (Fig 36) [18] and is craniocaudally flattened in comparison with the rounded distal end. The lateral aspect of the proximal end tapers to a laterally directed flange (Fig 36), the mirror image of the medially-directed flange in metatarsal III. A lateral flange is not present in *Neimongosaurus* [14]. The medial side of the proximal end forms a depression cupping the adjacent metatarsal. The midshaft of the bone is more constricted in comparison with the expanded proximal and distal ends and is less robust than the shaft of metatarsal III (Fig 36) as in *Falcarius* and *Segnosaurus* [6, 18]. Distally there is an expanded medial condyle and an extremely small, incipient lateral condyle as in *Falcarius* [18]. There is a pronounced embayment on the distocaudal surface between the two condyles.

#### Pedal phalanges

**Fig 37:**
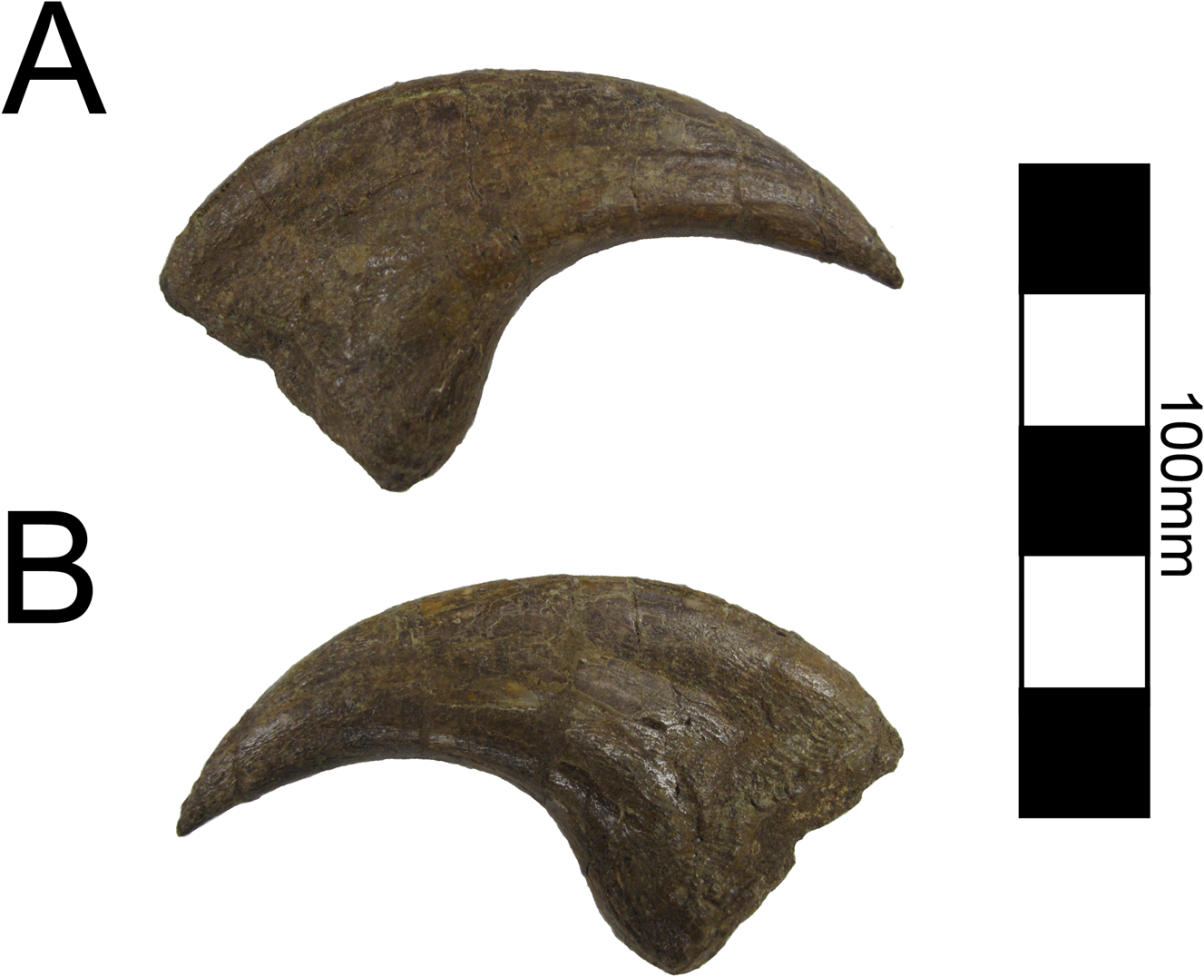
*Nothronychus* (MSM P2117) pedal ungual. Pedal ungual in side views. Digit unknown. Scale = 100 mm.

A total of eleven pedal phalanges are preserved with UMNH VP16420 (*No*. *graffami*) and three are associated with MSM P2117 (*No*. *mckinleyi*). Considering the higher degree of completion of the pes in UMNH VP16420, the discussion of pedal phalanges follows UMNH VP16420 since there are no clear morphological differences between the pes of the two species. Following Zanno et al. [2], the right foot is missing I-2, III-2, III-4, and IV-5 (Fig 36). The proximal phalanx of digit I is preserved. Proximally it is bowl-shaped and not bifurcated. Distally, there are two distinct condyles with deep collateral ligament pits as in *Segnosaurus* and *Therizinosaurus* [5, 6]. The left PI-1 is preserved for MSM P2117 and is similar in size and shape to PI-1 in UMNH VP16420. Digit II preserves PII-1 and PII-2. PII-1 is the largest phalanx preserved as in *Therizinosaurus* [5]. The ligament pits on PII-1 are less pronounced than in PI-1, but otherwise PII-1 has the same shape as PI-1. PII-2 is approximately the same size as PI-1, but has a different proximal surface for articulation with the two distal condyles of PII-1. PII-2 is nearly subequal in length with PII-1, but is much less robust. PIII-1 is identical to PII-1. PIII-2 is missing. PIII-3 is identical to PII-2. In *Alxasaurus*, there are no ligamentous pits on these phalanges [12], but they are present on UMNH VP16420. PIV-1 is the smallest proximal phalanx and is two-thirds the length of II-1 and III-1 as in *Falcarius* and *Therizinosaurus* [5, 18], while maintaining the same robusticity of the proximal and distal surfaces. PIV-2, PIV-3, and PIV-4 are preserved and are progressively smaller than PIV-1. Based on the similarities to UMNH VP16420, it is possible to refer the remaining two small phalanges preserved with MSM P2117 to PIV-2 and PIV-4. Two pedal unguals are preserved in MSM P2117 and one in UMNH VP16420. The pedal unguals in both specimens are robust, though are substantially smaller than the manual unguals as in other therizinosaurians (Fig 37). They are approximately the same length as the pedal phalanges and are gently recurved. The pedal unguals are transversely expansive rather than compressed like the manual unguals. This robusticity is especially evident on the articulation facets.

## Discussion

### Taphonomic Description

**Fig 38:**
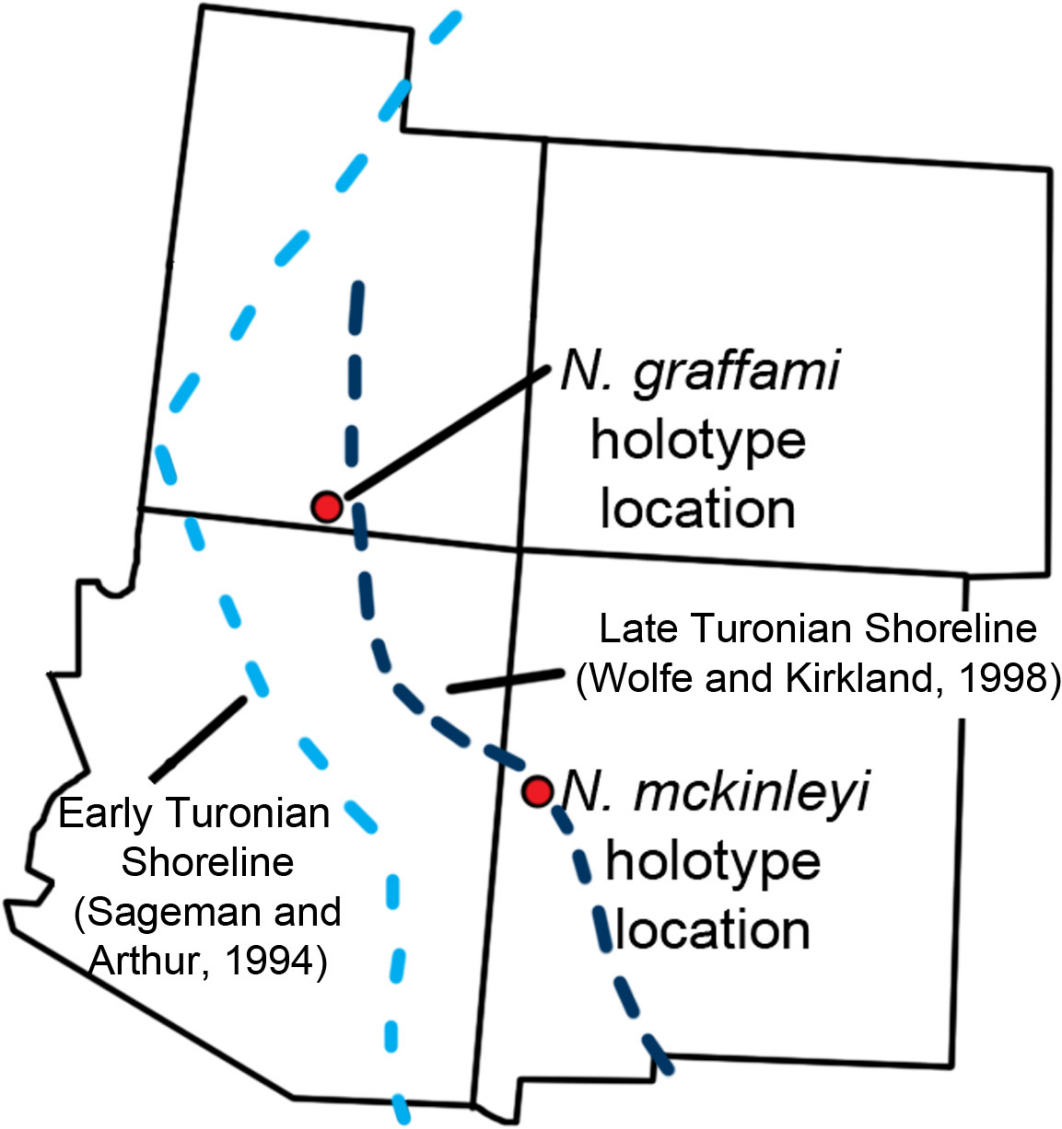
Location map. The locations of the quarries producing UMNH VP16420 and MSM P2117 with US states overlaid. This is in relation to the estimated Turonian shoreline (light blue shoreline based on Sageman and Arthur [58]; dark blue shoreline based on Wolfe and Kirkland [27]). The shoreline reconstructed by Sageman and Arthur [58] is based on the early Turonian and that by Wolfe and Kirkland [27] is for the late Turonian demonstrating a regression of the Western Interior Seaway during the Turonian. This shows that during the early Turonian UMNH VP16420 was offshore.

In addition to the importance of osteological characterizations of *No*. *mckinleyi* and *No*. *graffami*, it is critical to put these species into a geological context. MSM P2117 (*No*. *mckinleyi*) and UMNH VP16420 (*No*. *graffami*) were recovered from strata of similar age (early-middle Turonian) and are nearby geographically (Fig 38), yet are known from very different depositional environments. MSM P2117 was found in the Moreno Hill Formation (~92 Ma) of New Mexico along with the ceratopsian *Zuniceratops* [23, 46], the hadrosauroid *Jeyawati* [47], an unnamed tyrannosaur, and abundant, and exceptionally preserved fossil wood [48]. UMNH VP16420 is known from the Tropic Shale (~92.5–93 Ma) of southern Utah along with polycotylid plesiosaurs and other marine taxa [28]. A short description of the taphonomy of the bonebed where MSM P2117 was found has been published [49] and the geology of the UMNH VP16420 locality was briefly described in reference to other vertebrate taxa discovered at the locality [28]. However, neither of these localities has been described extensively and they have not been stratigraphically correlated.

The locality that produced MSM P2117 (*No*. *mckinleyi*) is known as the Haystack Butte Quarry, and is located in the Moreno Hill Formation, southwestern Zuni basin in west-central New Mexico. The Cretaceous section in the Zuni Basin is composed of terrestrial, estuarine, nearshore, and marine rocks indicative of the transgression and regression sequences of the Western Interior Seaway during the Late Cretaceous. The Moreno Hill Formation itself represents a terrestrial environment during a regressive phase of the Western Interior Seaway.

Several recent studies [27, 50–52] have generated a better understanding of the stratigraphic placement of the Zuni Basin Cretaceous section, including refined placement of the Moreno Hill Formation. The Moreno Hill Formation overlies the Atarque Sandstone [51], which has produced both the bivalve *Mytiloides labiatus* and the ammonite *Collignoniceras wollgari* indicative of an early–middle Turonian age [27]. McLellan et al. [51] subdivided the Moreno Hill Formation into lower, middle, and upper members. The dinosaur fossil assemblage including MSM P2117 is located in the middle of the lower member [27]. This section of the Moreno Hill Formation is likely correlated to the Torrivio Sandstone member of the Gallup/Crevasse Canyon complex [27, 53, 54] and is middle Turonian in age (~92 Ma).

**Fig 39:**
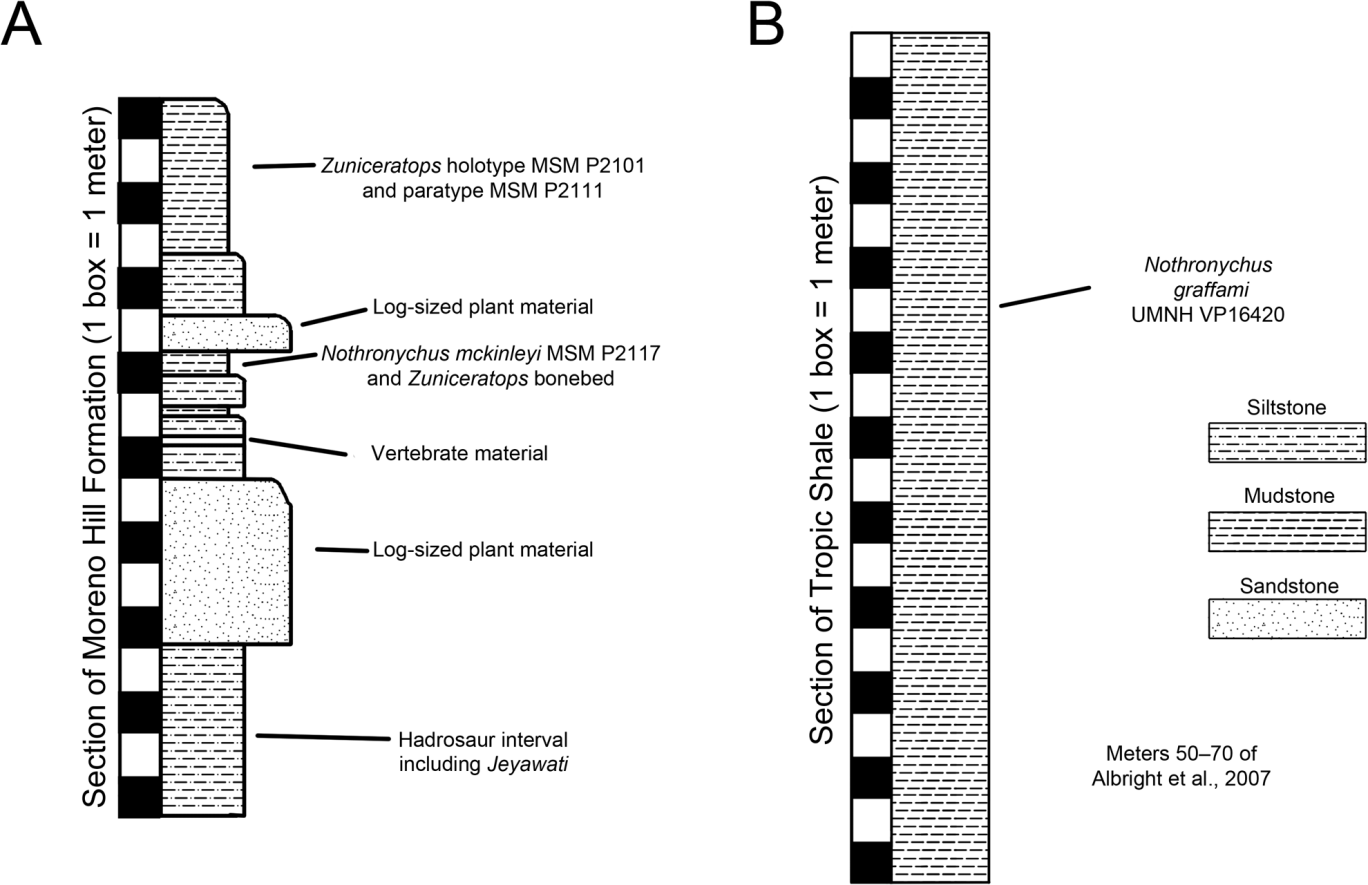
Stratigraphic sections. (A) The stratigraphic section of the Moreno Hill Formation in west-central New Mexico showing the relationship of the MSM P2117 Haystack Butte Quarry in relation to the other dinosaur discoveries in the area (Based on Wolfe and Kirkland [27]). (B) The stratigraphic section of the Tropic Shale showing the location of the UMNH VP16420 locality (Based on Albright et al. [28]). 1 box = 1 meter.

The bone-producing section of the Haystack Butte Quarry [27] is seventeen meters thick and is composed of alternating claystone, siltstone, and sandstone with one silicrete concretionary layer of less than 20 cm (Fig 39A) [27]. Dinosaur bones are found primarily in the mudstone layers. MSM P2117 was discovered with abundant *Zuniceratops* material in a paucispecific bone bed, four meters below the *Zuniceratops* holotype specimen, two meters above the unnamed theropod specimen, and eight meters above *Jeyawati*. Two channel sandstones that have substantial log material embedded in them bracket the bone bed. Logs and other plant material are common in the sandstone intervals. The lower sandstone is a four-meter thick fluvial channel sandstone with crossbedding demonstrating that the paleoenvironment was a high-energy fluvial system [49].

**Fig 40:**
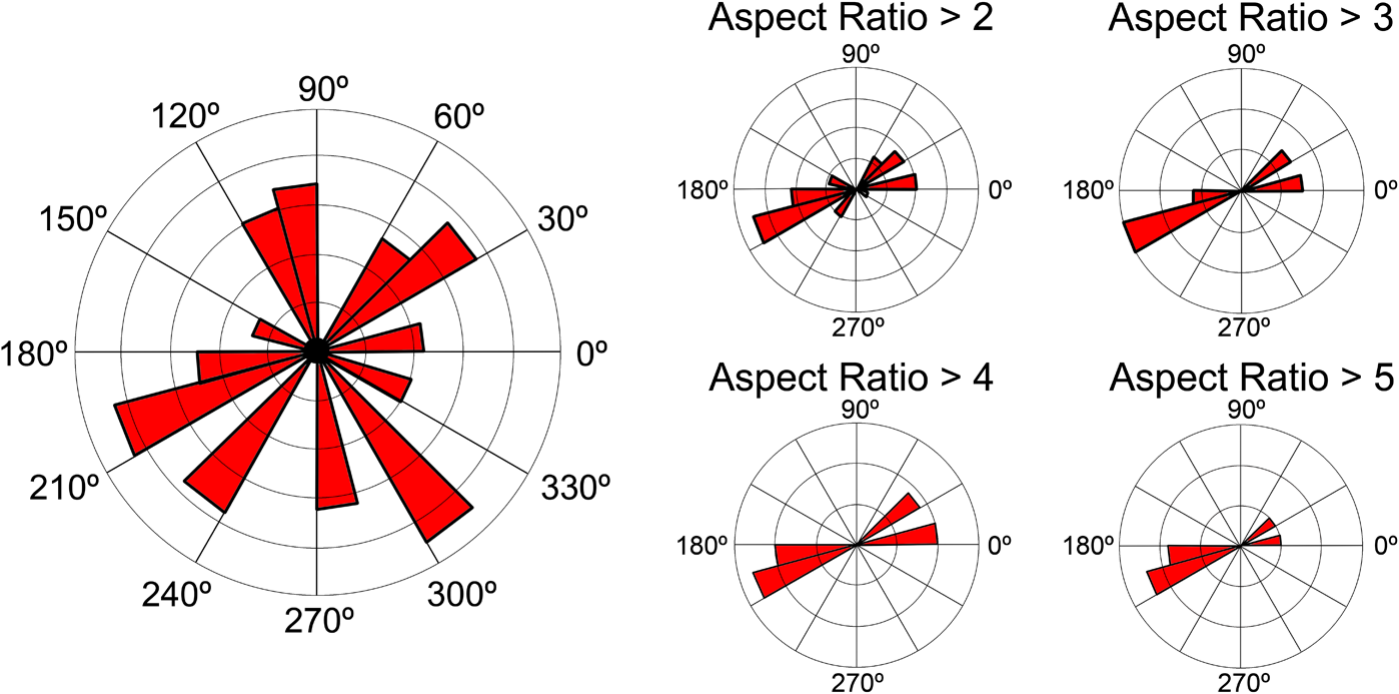
MSM P2117 Bonebed Rose Diagram. A rose diagram showing the orientation of all specimens preserved in the Haystack Butte Quarry with subsets of the data split by aspect ratios.

The condition of the bones indicates some predation and subaerial weathering, although there was not prolonged exposure of the bones since many retain well-preserved cortical bone [49]. The paucispecific bone bed measures 14 meters by 6 meters and is 1 meter thick. It preserves numerous disarticulated specimens attributed to *Zuniceratops*, all the preserved material of MSM P2117, and a large number of logs. The material is completely disarticulated with many of the bones of MSM P2117 spaced meters apart with logs or *Zuniceratops* material separating them. For example, the ischia of MSM P2117 were found three meters apart. The distribution and frequency of specimen orientations were sorted into aspect ratio categories with a length: width ratio greater than 2, 3, 4, and 5 (S1 Table). Specimens with aspect ratio greater than 2:1 are clustered into bimodal frequencies relative to the compass directions and are greatest at two orientations of 75–90° apart (S1 Table; Fig 40). The higher the aspect ratio is, the more substantial the preferred orientation is, suggesting that longer elements are more likely to be preferentially oriented. This further supports a fluvial deposition of specimens under the influence of strong currents. It is likely that the logs acted as a trap for the *Nothronychus* and *Zuniceratops* materials causing them to become concentrated.

In contrast with the fluvial system where MSM P2117 was located, UMNH VP16420 (*No*. *graffami*) was discovered in a deep-water shale bed in the Tropic Shale from southern Utah, nearly 325 km northwest of the Haystack Butte Quarry. The Tropic Shale is bounded by the underlying Naturita Formation and overlying Straight Cliffs Formation and is roughly equivalent to the temporally and geographically extensive Mancos Shale [55, 56]. The Tropic Shale spans from 94 to 90.5 Ma [28]; however, the strata producing UMNH VP16420 can be constrained to between 93.5 to 92 Ma (early Turonian) based on the lowest occurrence of the ammonoid *Mammites nodosoides* [57]. UMNH VP16420 was found 5 meters below this lowest occurrence and 65 meters above the contact with the underlying Naturita Formation [2, 28, 56] (Fig 39B). In addition to UMNH VP16420, other vertebrate material found in the strata includes the polycotylids *Trinacromerum*, *Polycotylus*, and *Palmula* as well as turtle and fish specimens [28].

The western paleoshoreline of the Western Interior Seaway is considered to have been approximately 100 km west of the field site that produced UMNH VP16420 during the early Turonian based on reconstructions by Sageman and Arthur [58] (Fig 38). This indicates that *No*. *graffami*, a clearly terrestrial animal, must have died and floated a long distance on the ocean before sinking and being buried by sediment. Although this is an improbable taphonomic mode, it is not unknown in dinosaurs. The marine Smoky Hill Chalk has produced dinosaur material, including nodosaurs, as well as other terrestrial vertebrate taxa [59, 60]. These localities are more than 100 km from reconstructed paleoshorelines suggesting that ‘float and bloat’ was the taphonomic mode for these taxa [59]. The ‘float and bloat’ hypothesis is also presumed to be the preservational mode for UMNH VP16420. Though a large number of bones are present for UMNH VP16420, the overall preservation of the bone is quite poor. The bones are diagenetically flattened and sheared, making interpreting dimensions and orientation of osteological structures problematic.

The holotypes of *No*. *mckinleyi* and *No*. *graffami* are geographically separate and are also separated temporally by likely 1–1.5 million years and possibly up to 3 million years (*No*. *mckinleyi* representing a younger taxon). UMNH VP16420 (*No*. *graffami*) is known from a section below the *Mammites nodosoides* biozone well constrained to the early Turonian (93.5 to 92 Ma) and MSM P2117 (*No*. *mckinleyi*) was recovered above the *Collignoniceras woolgari* biozone constrained to the middle Turonian (~92 Ma), demonstrating clear temporal separation between the two species. Further, UMNH VP16420 was preserved during a transgression while MSM P2117 was preserved during a regression. The peak transgression during this period occurred during the early Turonian [52] with a regression in the middle Turonian resulting in the deposition of the Atarque Sandstone and Moreno Hill Formation.

The precise geographical separation of these two specimens is unknown because UMNH VP16420 was preserved in an allochthonous environment. It is presumed that MSM P2117 did not travel far from where it died due to the good preservation of the bone surface, lack of breaks in the bone, and the fact that a fluvial system would not be able to carry large bones more than several kilometers. However, UMNH VP16420 must have been carried approximately 100 km from where it lived and died, as it is a terrestrial animal. Although the distance between the localities where each taxon was discovered is 325 km, their separation in life was potentially much greater.

### The Effect of Diagenesis on Morphology

The contrasting taphonomic histories of each animal led to different preservation regimes and degrees of diagenetic deformation. In general, MSM P2117 (*No*. *mckinleyi*) exhibits minimal diagenetic deformation as a result of a reasonably fast burial in fluvial sediments. However, UMNH VP16420 (*No*. *graffami*) underwent extensive diagenetic deformation as a result of intensive compression in shale sediments. As such, we have taken careful consideration to address diagenetic deformation and its impact on the morphology of this specimen throughout.

One side of all bone elements in UMNH VP16420 is extremely poorly preserved and the bone surface is often degraded or missing. Many characters that distinguish taxa are related to the angulation of certain structures, which can be modified by plastic deformation [36]. Hedrick and Dodson [37] showed that a number of characters that were originally considered to separate three species of *Psittacosaurus* were caused by diagenetic distortion and were not valid and that the three taxa actually belonged to a single taxon. Therefore, it is critical to temper osteological assessments with the potential inputs of diagenetic deformation.

As has been previously noted, sauropod vertebrae are especially susceptible to diagenetic deformation because of their fine structures and thin, pneumatic bone [61]. Therizinosaurian vertebrae also exhibit highly pneumatic, complex cervical and dorsal vertebrae and they are also easily affected by deformation. Though both MSM P2117 (*No*. *mckinleyi*) and UMNH VP16420 (*No*. *graffami*) preserve cervical vertebrae, very little can be said about their morphology due to breakage and plastic deformation (Figs 4 and 5). The dorsal vertebrae in UMNH VP16420 are also problematic. They are more robust than the cervical vertebrae and it is therefore much easier to comment on their structure. However, due to the high degree of deformation in UMNH VP16420, it is difficult to determine whether certain features evident in the dorsal vertebrae are biologic or diagenetic in nature, such as the extent or presence of individual laminae and even length: width ratios of the centra. Due to these considerations no diagnostic characteristics of the presacral vertebrate have been used to distinguish *No*. *graffami* and *No*. *mckinleyi*.

Breakages are also an ever-present issue in paleontological morphology studies. The general shearing of one side of the bone in the majority of the UMNH VP16420 elements greatly limits the ability to interpret material. For example, breakages along the distal scapular blade in both UMNH VP16420 and MSM P2117 do not allow for the assessment of whether or not the distal scapular blade is expanded or tapered. *No*. *mckinleyi* has been coded in phylogenetic analyses as possessing this character [2]. Kirkland and Wolfe [16] first interpreted the scapulae of MSM P2117 as tapering, but we were not able to demonstrate that here due to the presence of breaks.

Relative angulations of bones are also an issue. The ulnae of UMNH VP16420 were considered straight rather than curved, a character used to distinguish *No*. *graffami* from *No*. *mckinleyi* in previous phylogenetic analyses [2, 19]. Given the taphonomic environment of UMNH VP16420 and the condition of the material, it is unlikely that a curved ulna would be preserved and it would more likely be straightened during compression. As such, we have eliminated this feature from the characteristics distinguishing the two *Nothronychus* species.

In our revisions to the diagnoses of *No*. *graffami* and *No*. *mckinleyi* we have excluded the characters described above that are likely to have been affected by distortion. Even so, the morphological differences between the two taxa are relatively minor and therefore it is possible that the variation we attribute to species-level differences actually represent individual variation or unrecognized deformation. Although current data support the validity of these two species, discovery of more complete material of *No*. *mckinleyi* or diagenetically unaltered material of *No*. *graffami* may demonstrate that the two taxa are actually the same species that occupied a substantial geographic and temporal range.

## Conclusions

Although therizinosaurians were a poorly known group as recently as the late 1990s, the discovery of a number of taxa in China and North America has greatly expanded our understanding of this important group. However, detailed descriptions of therizinosaurian taxa remain sparse. This analysis provides the most complete description of derived therizinosaurians to date and a taphonomic evaluation of the holotype localities.

Both species of *Nothronychus* are reexamined and found to be distinguishable based on a number of characters. Generally, *Nothronychus* is similar to other therizinosaurids and the closely related therizinosauroid, *Suzhousaurus*. *Nothronychus* is the most complete therizinosaurid genus known and exemplifies the highly pneumatic vertebrae, reduced caudal vertebral count, enlarged manual unguals, enlarged and fully retroverted sloth-like pelvis, and functionally tetradactyl pes that are characteristic of derived therizinosaurs. We also reevaluate the characters distinguishing the *Nothronychus* species from one another and from other therizinosaurian genera amending some previous characters.

*No*. *mckinleyi* is a younger taxon than *N*. *graffami* [27, 55]. The beds producing *No*. *mckinleyi* overlie the Atarque Sandstone, which contains the *Collignoniceras woollgari* biozone and are referable to the middle Turonian. Albright et al. [28] showed that *No*. *graffami* is found in slightly older rocks temporally calibrated by the *Mammites nodosoides* biozone, indicating an early Turonian age. The temporal separation between the two species may be up to three million years. Further, the species are found in different environments: *No*. *mckinleyi* in a fluvial channel and *No*. *graffami* in deep sea sediments. Contrasting taphonomic factors clearly impacted the preservation of the two species. We interpret some morphological differences between these specimens to be the result of diagenetic distortion in *No*. *graffami*, whereas others appear to have biological significance. It is critical to consider the effects of diagenetic deformation on morphology when analyzing characters as demonstrated for these taxa.

## Supporting Information

S1 Fig Cervical rib of MSM P2117. Scale = 100 mm.
(TIF)

S2 Fig Selected chevron elements of UMNH VP16420. Scale = 100 mm.
(TIF)

S3 Fig Selected gastralia of UMNH VP16420. Scale = 100 mm.
(TIF)

S4 Fig Line drawing of Fig 4. Scale = 100 mm.
(TIF)

S5 Fig Line drawing of Fig 5A and 5B. Scale = 100 mm.
(TIF)

S6 Fig Line drawing of Fig 7. Scale = 100 mm.
(TIF)

S7 Fig Line drawing of Fig 8. Scale = 100 mm.
(TIF)

S8 Fig Line drawing of Fig 9. Scale = 100 mm.
(TIF)

S9 Fig Line drawing of Fig 10. Scale = 100 mm.
(TIF)

S10 Fig Line drawing of Fig 15A. Scale = 100 mm.
(TIF)

S1 Multimedia Zip File containing surface scans of various bone elements of MSM P2117 (*No*. *mckinleyi*) and *No*. *graffami* (UMNH VP16420). (A) UMNH VP16420 caudal vertebra (B) UMNH VP16420 caudal vertebra (C) UMNH VP16420 caudal vertebra (D) UMNH VP16420 left scapula (E) UMNH VP16420 right scapula (F) UMNH VP16420 humerus (G) UMNH VP16420 ulna (H) UMNH VP16420 radius (I) UMNH VP16420 manual claw (J) UMNH VP16420 pubis (K) UMNH VP16420 ischium (L) UMNH VP16420 femur (M) UMNH VP16420 tibia (N) UMNH VP16420 fibula (O) MSM P2117 caudal vertebra (P) MSM P2117 scapula (Q) MSM P2117 left humerus (R) MSM P2117 right humerus (S) MSM P2117 antebrachial element (T) MSM P2117 ulna (U) MSM P2117 manual phalanx (V) MSM P2117 left ischium (W) MSM P2117 left tibia (X) MSM P2117 right tibia (Y) MSM P2117 fibula (Z) MSM P2117 metatarsal (AA) MSM P2117 pedal claw (BB) MSM P2117 gastralia (CC) MSM P2117 gastralia.
(ZIP)

S1 Table Sheet 1: The ordering of the caudal vertebrae of UMNH VP16420 based on field numbers for identification. Sheet 2: Selected measurements of MSM P2117. Sheet 3: Selected measurements of UNMH VP16420 (based on Zanno et al., 2009). Sheet 4: Measurements of the long bones of therizinosaur appendicular elements. Sheet 5: MSM P2117 bone bed angle and aspect ratio data.
(XLSX)
