# Supplementary figures and images for: The Slothful Claw: Osteology and Taphonomy of Nothronychus mckinleyi and N. graffami (Dinosauria: Theropoda) and Anatomical Considerations for Derived Therizinosaurids
Source: PLoS One. 2015 Jun 10;10(6):e0129449. doi: 10.1371/journal.pone.0129449 (PMC4465624; doi:10.1371/journal.pone.0129449)

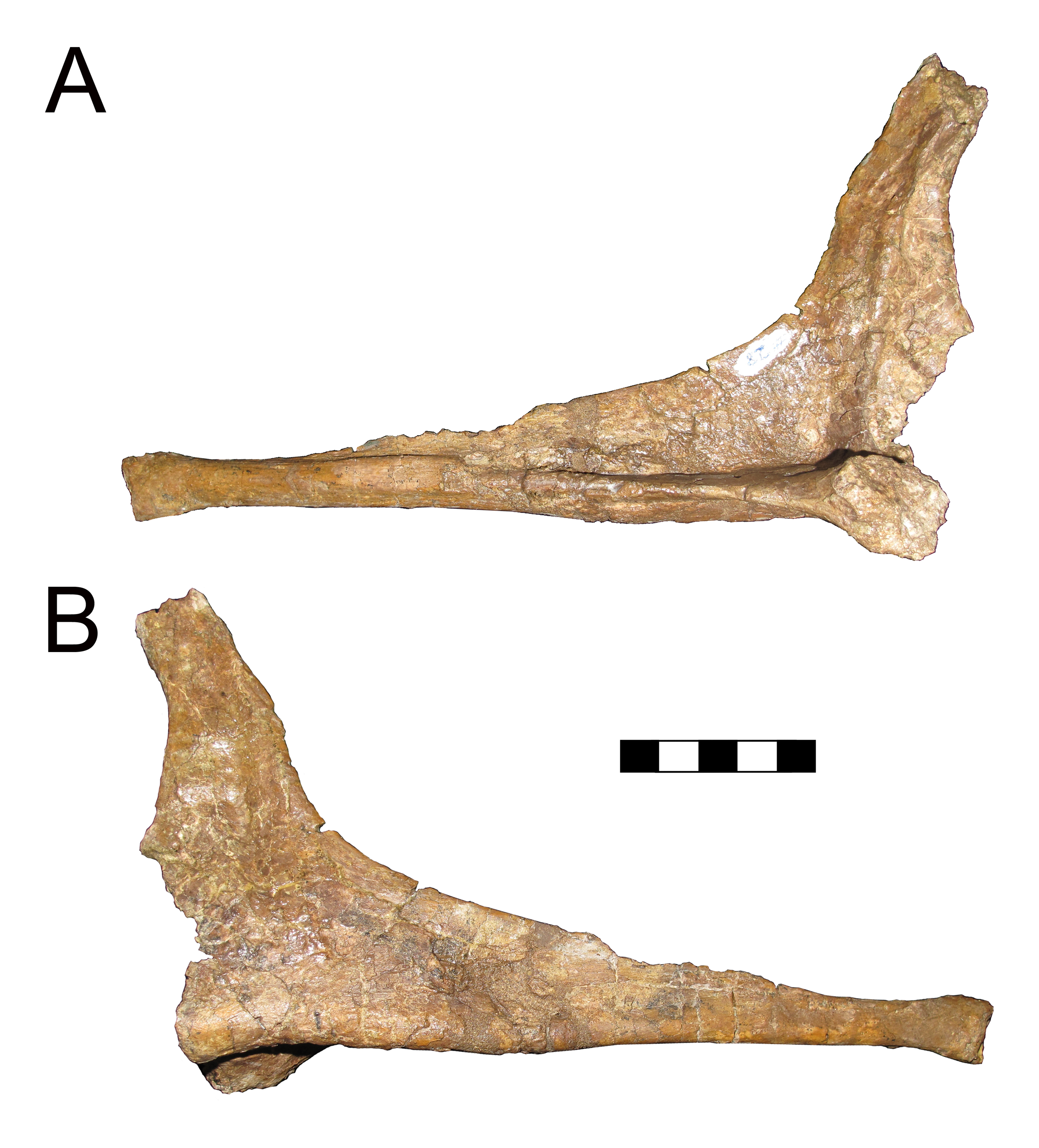

Supplement: S1 Fig — Scale = 100 mm. (TIF) [file pone.0129449.s001.tif]

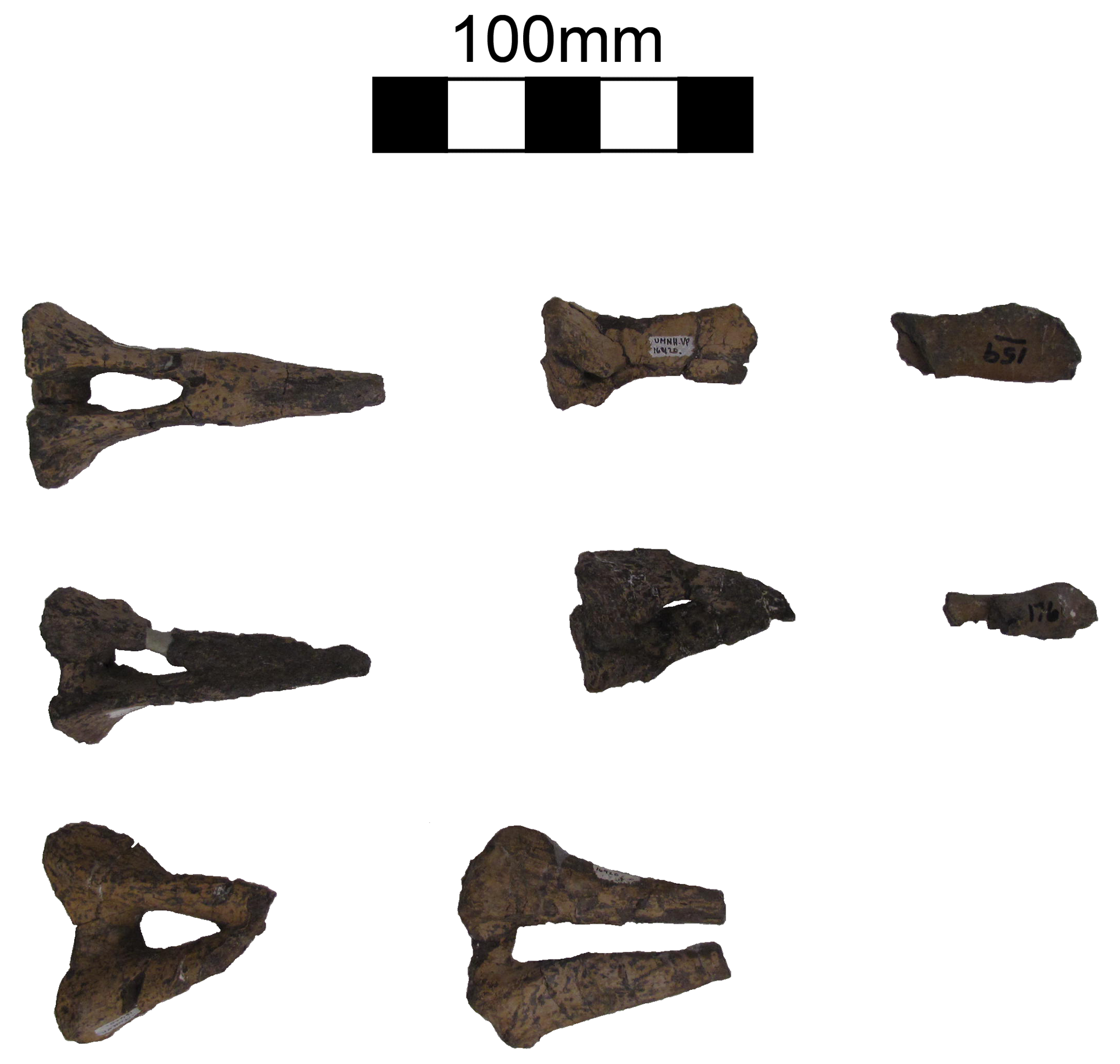

Supplement: S2 Fig — Scale = 100 mm. (TIF) [file pone.0129449.s002.tif]

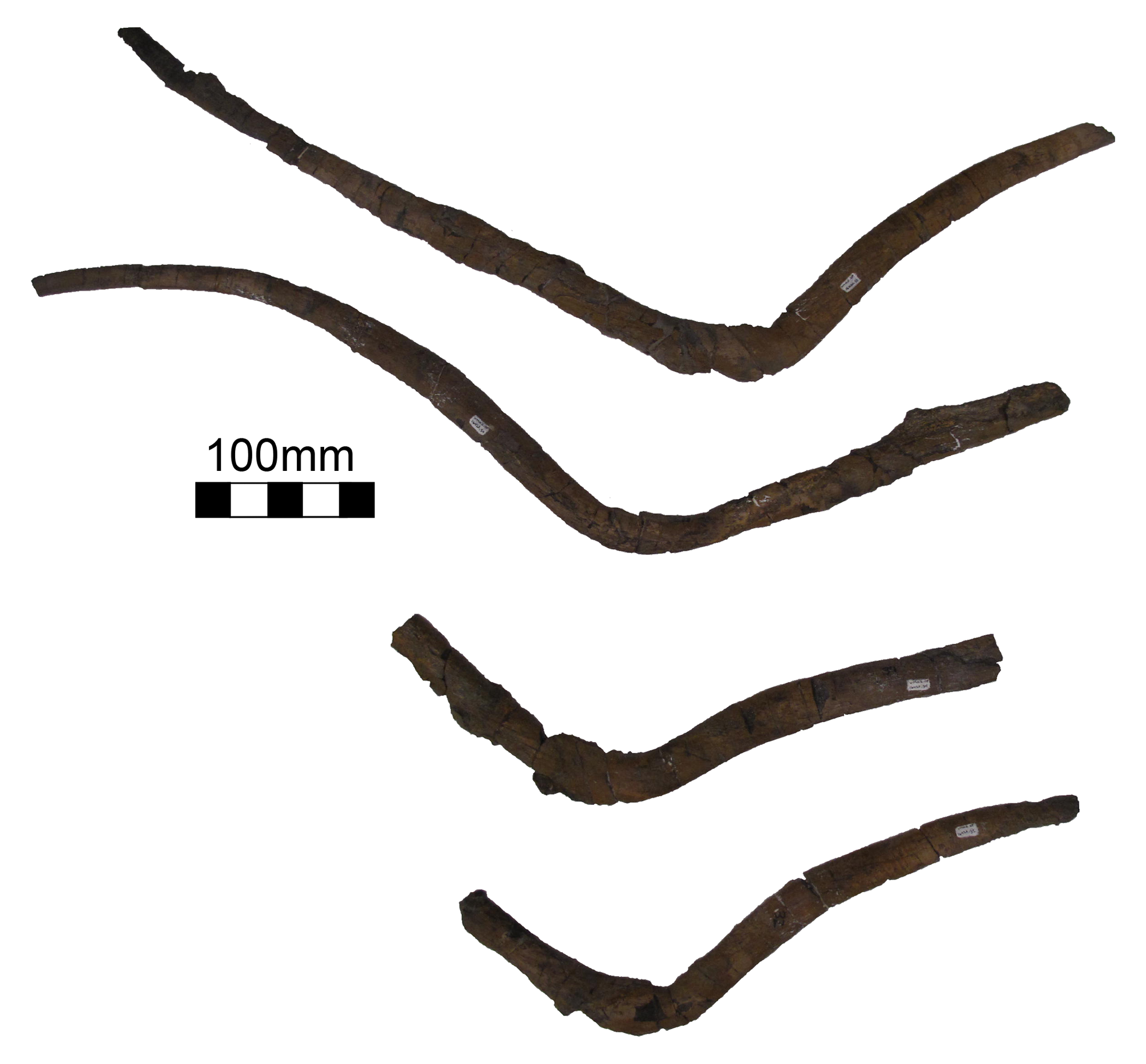

Supplement: S3 Fig — Scale = 100 mm. (TIF) [file pone.0129449.s003.tif]

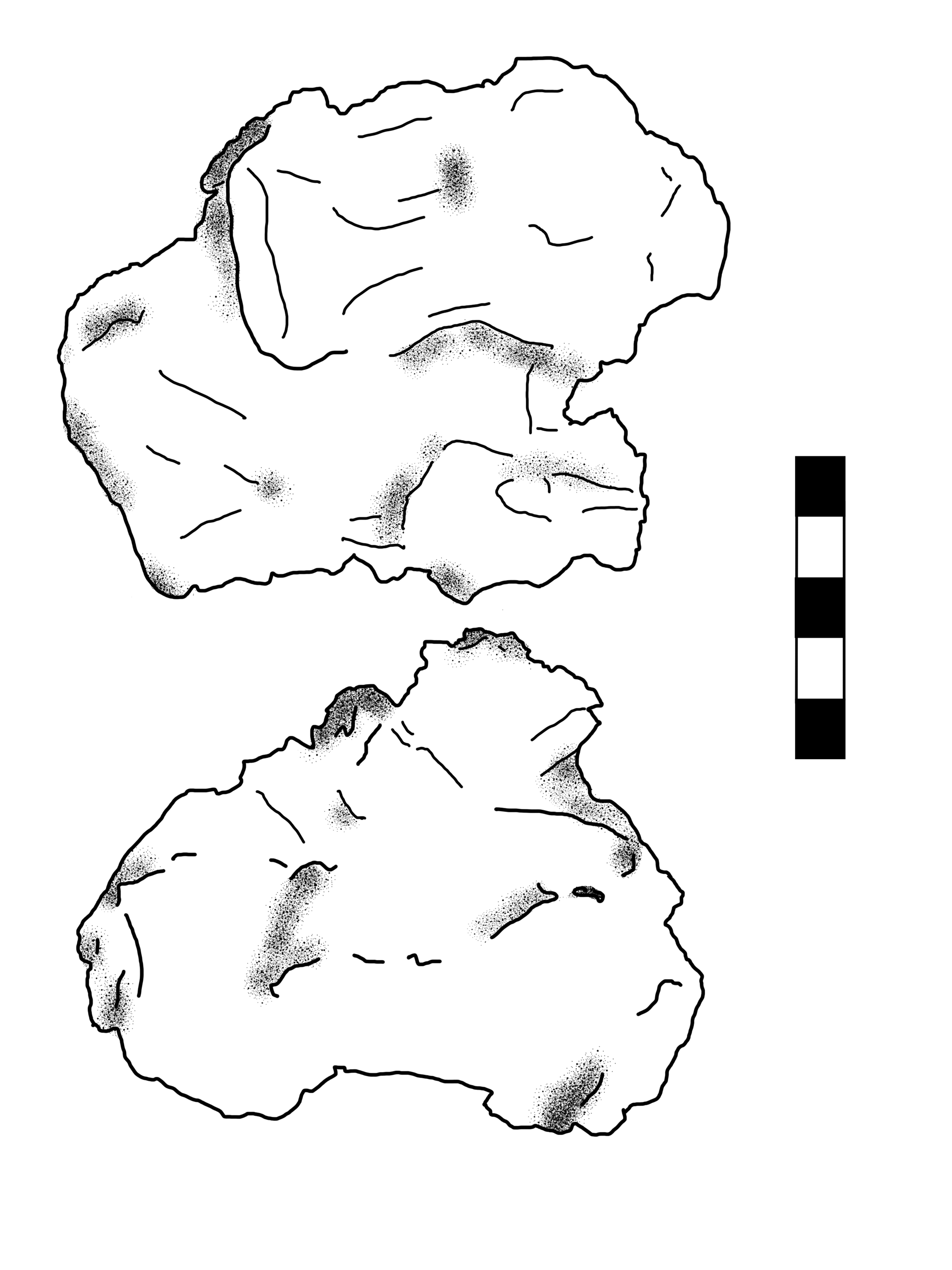

Supplement: S4 Fig — Scale = 100 mm. (TIF) [file pone.0129449.s004.tif]

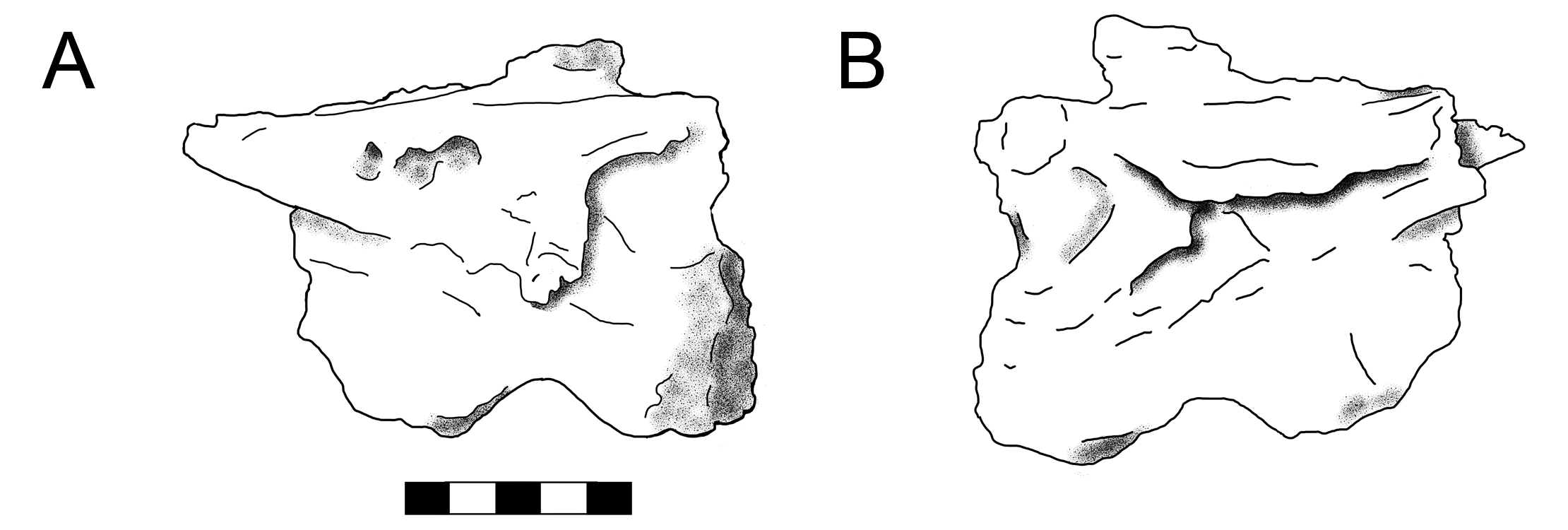

Supplement: S5 Fig — Scale = 100 mm. (TIF) [file pone.0129449.s005.tif]

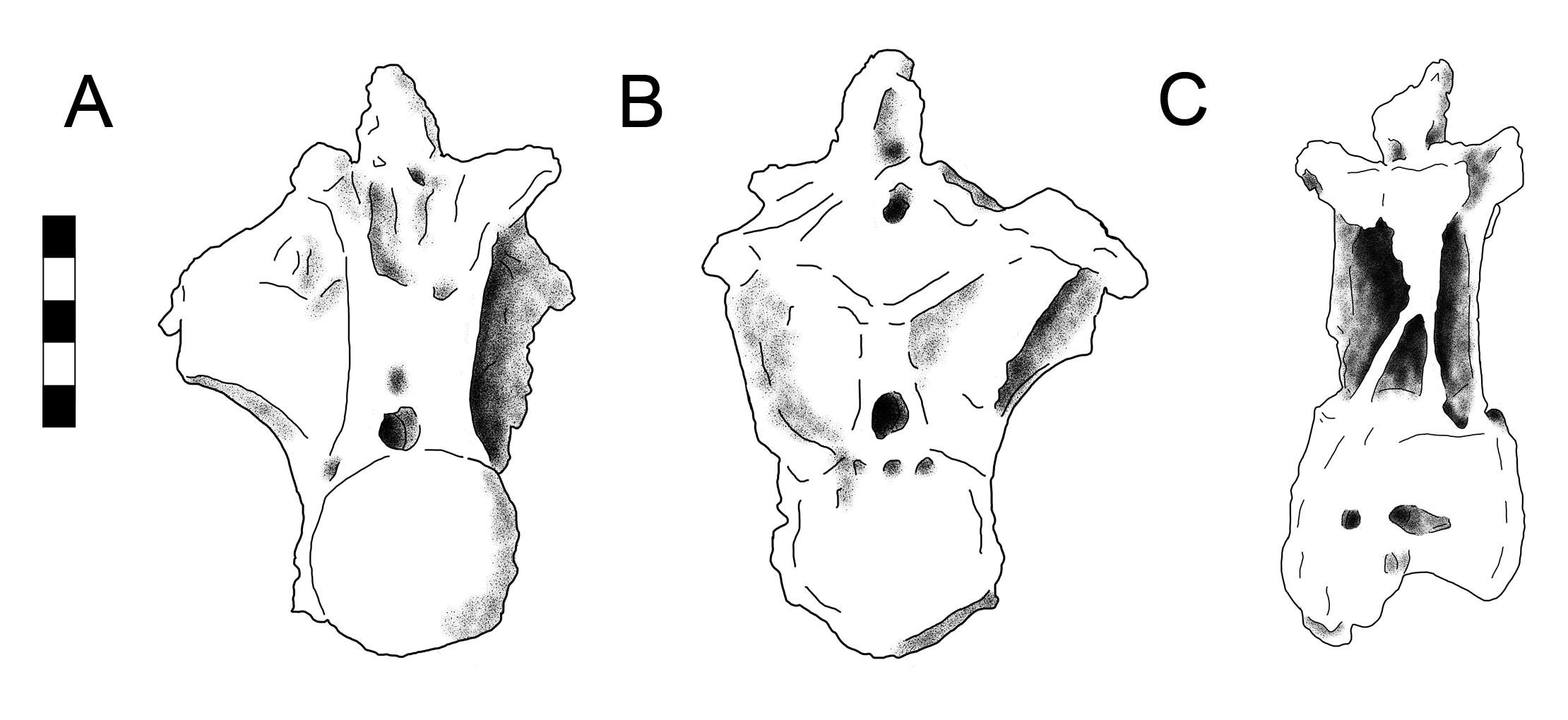

Supplement: S6 Fig — Scale = 100 mm. (TIF) [file pone.0129449.s006.tif]

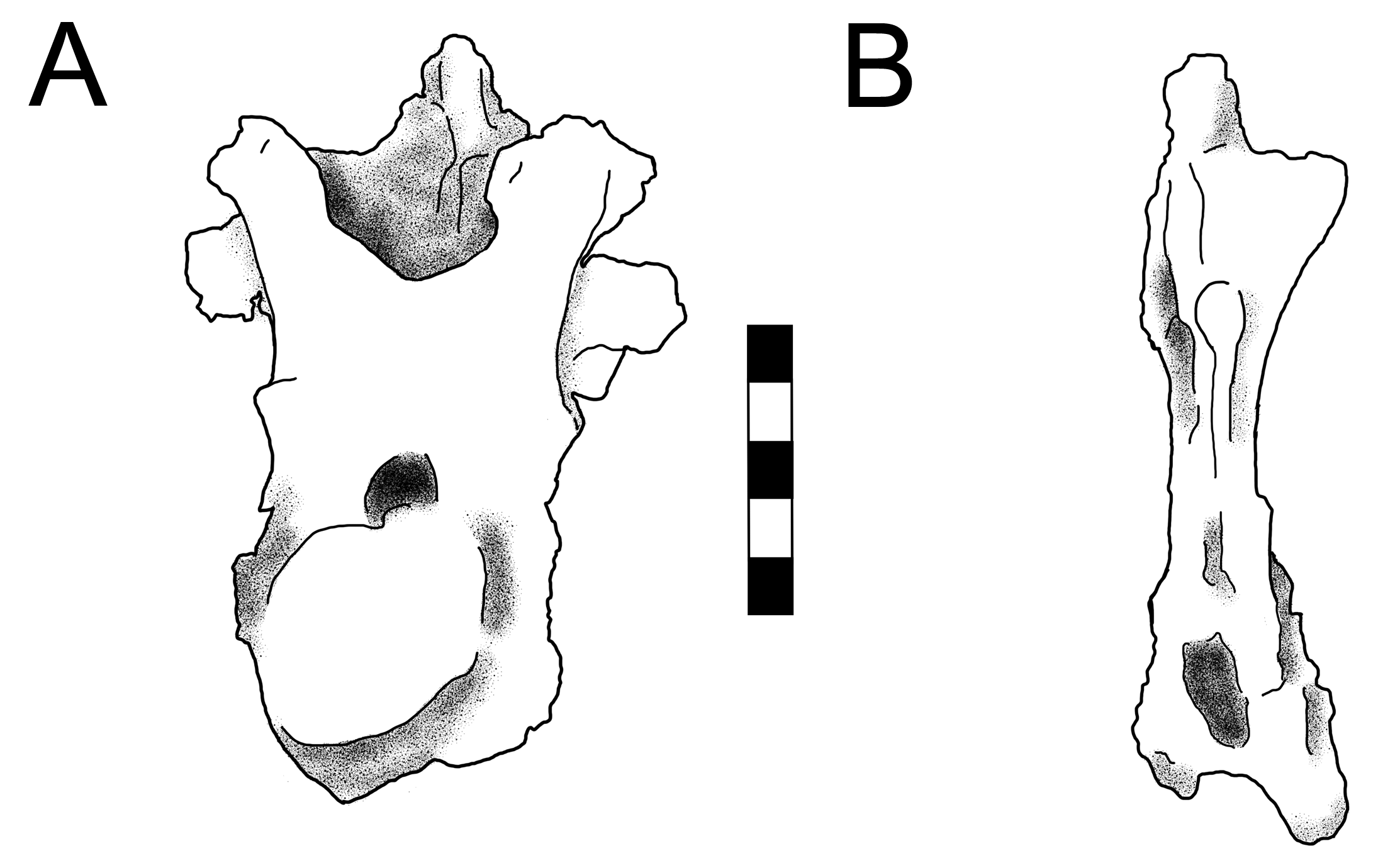

Supplement: S7 Fig — Scale = 100 mm. (TIF) [file pone.0129449.s007.tif]

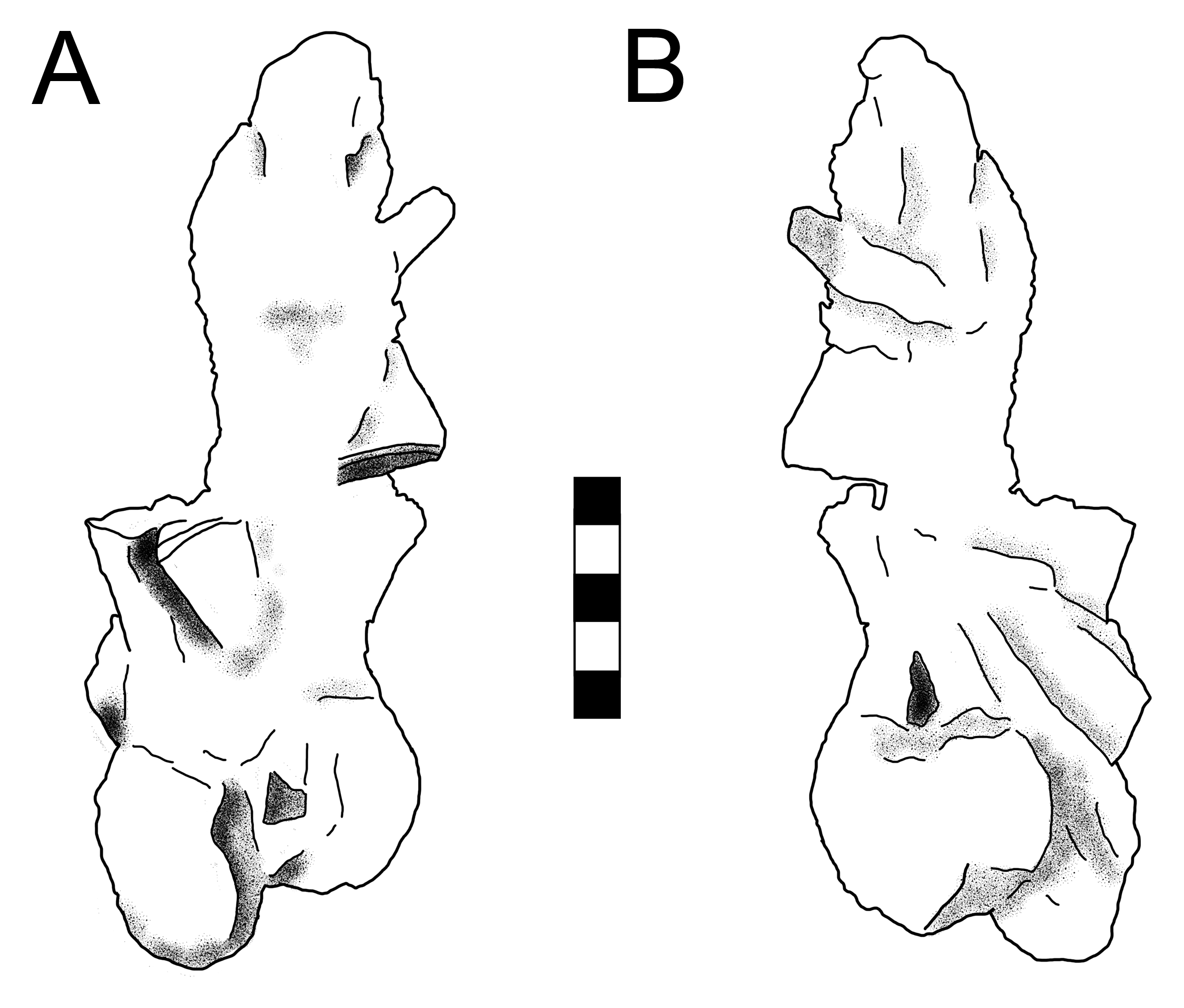

Supplement: S8 Fig — Scale = 100 mm. (TIF) [file pone.0129449.s008.tif]

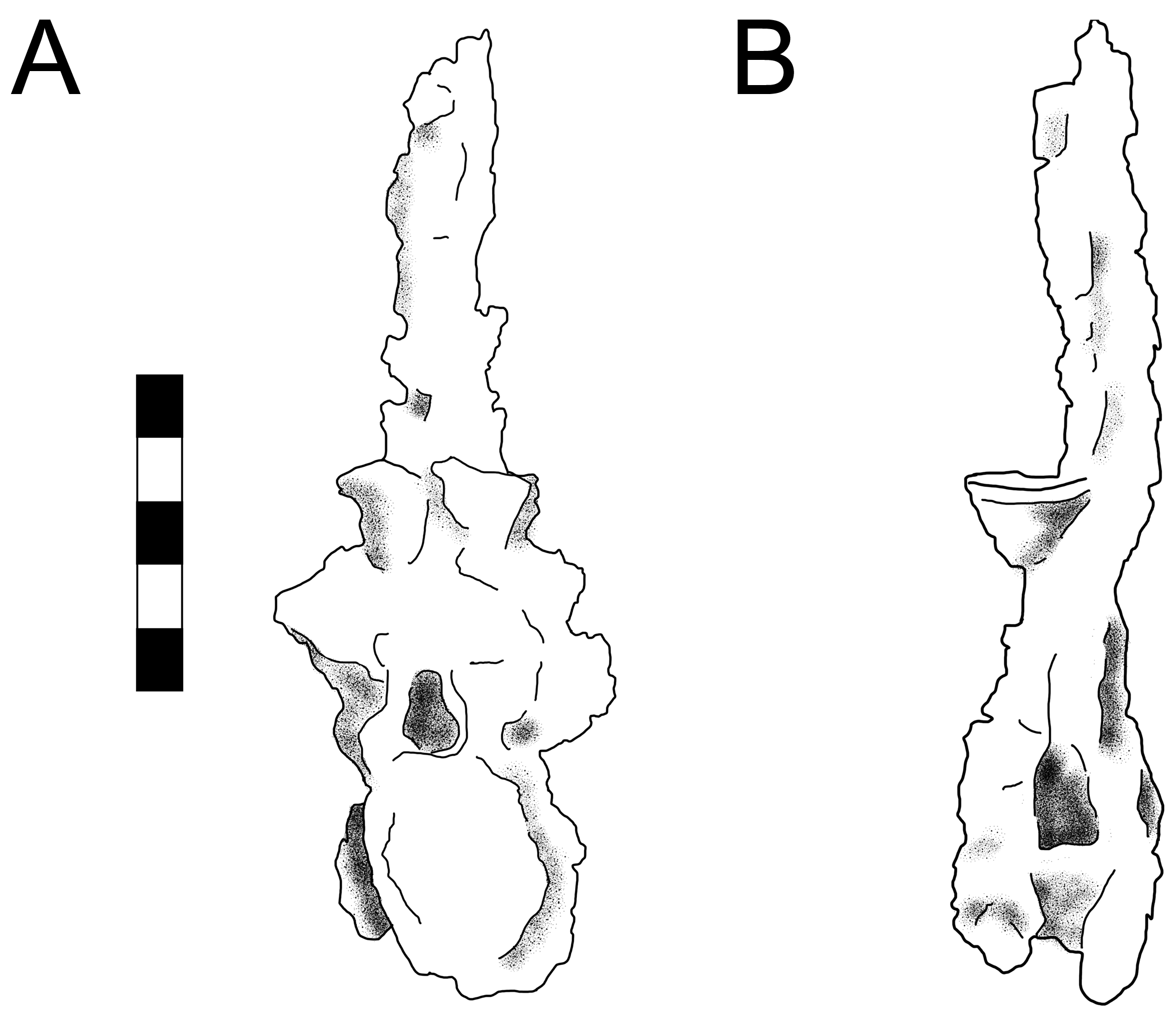

Supplement: S9 Fig — Scale = 100 mm. (TIF) [file pone.0129449.s009.tif]

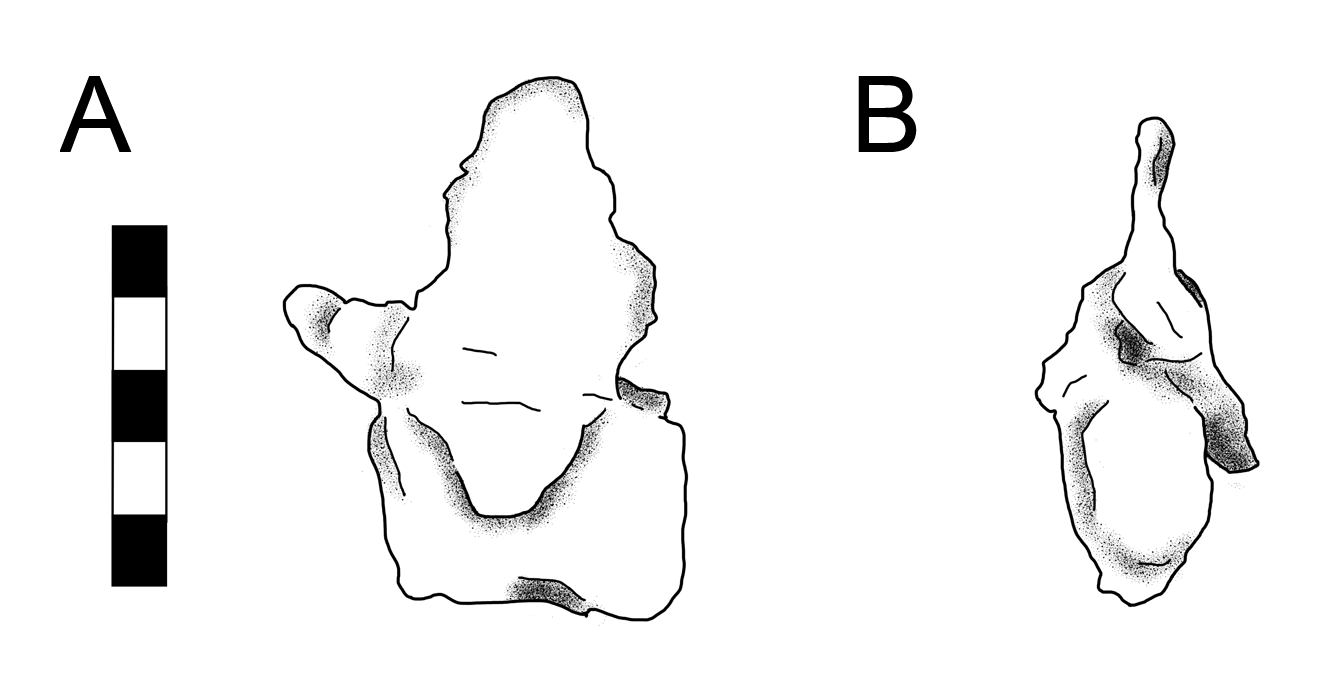

Supplement: S10 Fig — Scale = 100 mm. (TIF) [file pone.0129449.s010.tif]
